# A 17 GHz molecular rectifier

**DOI:** 10.1038/ncomms12850

**Published:** 2016-10-03

**Authors:** J. Trasobares, D. Vuillaume, D. Théron, N. Clément

**Affiliations:** 1 Institute of Electronics, Microelectronics and Nanotechnology, CNRS and University Lille 1, Physics Department, Avenue Poincaré, CS60069, 59652 Villeneuve d'Ascq, France; 2 NTT Basic Research Laboratories, NTT Corporation, 3-1, Morinosato Wakamiya, Atsugi-shi, Kanagawa 243-0198, Japan

## Abstract

Molecular electronics originally proposed that small molecules sandwiched between electrodes would accomplish electronic functions and enable ultimate scaling to be reached. However, so far, functional molecular devices have only been demonstrated at low frequency. Here, we demonstrate molecular diodes operating up to 17.8 GHz. Direct current and radio frequency (RF) properties were simultaneously measured on a large array of molecular junctions composed of gold nanocrystal electrodes, ferrocenyl undecanethiol molecules and the tip of an interferometric scanning microwave microscope. The present nanometre-scale molecular diodes offer a current density increase by several orders of magnitude compared with that of micrometre-scale molecular diodes, allowing RF operation. The measured *S*_11_ parameters show a diode rectification ratio of 12 dB which is linked to the rectification behaviour of the direct current conductance. From the RF measurements, we extrapolate a cut-off frequency of 520 GHz. A comparison with the silicon RF-Schottky diodes, architecture suggests that the RF-molecular diodes are extremely attractive for scaling and high-frequency operation.

## Abstract (web summary)

Molecular electronics holds promise to overcome scaling limits of conventional technologies, but is currently limited to low frequency operation. Here, Trasobares *et al*. show radio frequencies of up to 17.8 GHz in a molecular diode based on ferrocenyl undecanethiol self-assembled monolayers on gold nanodots.

## Graphical abstract

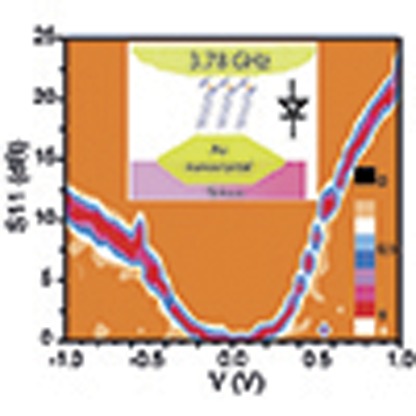

In the field of electronics, there is still a tremendous need for increasing devices, speed—more precisely the cut-off frequency *f*_T_ keeping the same direct current (d.c.) power consumption— in particular towards the THz frequency range. This frequency domain called the THz gap and corresponding to frequencies between the microwaves and far-infrared, is currently attracting a lot of attention. Electronic devices such as Schottky diodes, resonant tunnelling diodes or THz single-photon detectors continue to move into the THz range^1^,^2^.

In that context, molecular electronics, that is, small molecules connected between electrodes^3^, could play an important role with theoretically predicted transit times down to the fs^4^. Also, the field of molecular electronics is still very active from a basic research perspective^5^,^6^,^7^,^8^,^9^,^10^,^11^,^12^,^13^,^14^,^15^,^16^,^17^,^18^,^19^,^20^,^21^,^22^,^23^,^24^,^25^,^26^, allowing a rapid progress in devices performance and reliability. The experimental demonstration of a molecular diode as an example of a functional molecular electronic device has generated substantial interest since Aviram and Ratner proposed the pioneering idea of a molecular rectifier^7^. Experimental realizations have been made in both self-assembled monolayers (SAMs)^8^,^9^,^10^,^11^,^12^,^13^,^14^,^15^,^16^,^17^,^18^,^19^,^20^ or single-molecule^21^,^22^,^23^,^24^,^25^. Recently, significative progress was achieved based on a better understanding of the intermolecules or/and molecule/electrodes coupling^13^,^14^,^15^,^16^,^17^,^18^,^19^,^20^,^21^,^22^,^23^,^24^,^25^. However, so far, molecular diodes have only been demonstrated in the low-frequency regime, a serious limitation from a device perspective. A molecular half-wave rectifier was demonstrated, but the selected frequency of operation was 50 Hz^14^. Diodes based on high mobility organic semiconductors^27^ or hybrid materials composed of polymers/inorganic nanoparticles^28^ have recently enabled diodes operation at about 1 GHz by optimizing the mobility or charge injection at electrode contacts, but these systems are very different from that of molecular electronics, for example, electronic transport through small (typically 2–3 nm) molecules between two electrodes. To reach high frequency molecular components, several issues have to be addressed.

First, as pointed out in ref. 24, one main issue in these devices is that the conductance has remained extremely low^8^,^9^,^10^,^11^,^12^,^13^,^14^,^15^, seriously impacting the cut-off frequency of the device which could be approximated, in a simple dipole configuration, by *G/(*2*πC)*, with *G* and *C* the device dynamic conductance and capacitance, respectively. This limit was recently improved for single-molecule devices by chemical engineering of the molecule/electrode contact using a gold-carbon covalent bond (instead of the usual gold-sulfur-carbon link)^24^, but only d.c. measurements were shown. Also, it is well accepted that conductances per surface area (or per molecule) are orders of magnitude larger in nanometric or single-molecule junctions than in micrometric junctions^29^,^30^,^31^,^32^. This feature is often attributed to surface roughness and the presence of defects. An optimum number of molecules in parallel per junction has to be found, a single molecule being anyway limited by the quantum of conductance *G*_0_=77.5 μS. A second issue, barely adressed in molecular electronics, is the capacitive circuitry^33^,^34^,^35^,^36^ for frequency bandwidth optimization. Whereas the low dielectric constant (∼2–3)^37^ of organic monolayers should be an advantage, fringe capacitances may be dominant when reducing the devices dimension to single or few molecules. Other issues are instrumental: molecular electronics requires statistical studies for convincing demonstrations and it is not simple to implement hundreds of test devices with a top electrode compatible with high-frequency measurements. Also, high frequency measurement systems are mainly designed to measure devices near the 50 Ω regime, while molecular electronic devices have generally a ‘high-impedance'. Lots of work has been done on RF instrumentation at the nanoscale to overcome this limitation from both academic and industrial side^38^,^39^,^40^,^41^,^42^,^43^,^44^,^45^. More specifically to molecular electronics, a RF break-junction setup compatible with single-molecule device has been reported for an atomic contact device, but without presence of molecules^46^. AC-STM^47^,^48^,^49^,^50^,^51^,^52^,^53^, that combines a scanning tunnelling microscope (STM) with an alternative current RF field superimposed on the tip, has been successfully used to measure fundamental properties of molecules such as the polarizabilities of alkyl chains and π-conjugated oligomers^51^, the resonant oscillations (at 20 K) of a molecular chain physisorbed on a surface^52^, or the spin resonance of single terbium bis-phthalocyane molecule (at 5 K)^53^, but no functional device such as a molecular diode has been demonstrated at high frequency and at room temperature.

Here, we demonstrate RF molecular diodes operated up to 17 GHz with an estimated cut-off frequency of 520 GHz. The combination of a large array of single-crystal gold nanoelectrodes (few tens of nanometre in size corresponding to about 150 molecules in the molecular junction) and an interferometric scanning microwave microscope (iSMM^43^) were the key features to perform a statistical study on hundreds of molecular diodes with high dynamic conductances, *G*=*δI/δV* up to 0.36 mS and 110 aF range fringe capacitances. The origin of the large measured conductance is discussed, as well as the limiting speed parameters and the perspectives for getting new insights into molecular electronics transport from RF measurements. Finally, based on experimental data and simple models, we compare the RF-silicon Schottky and the RF-molecular diode architectures. We evidence the interesting perspectives of the molecular electronics approach for scaling and high-frequency operation.

## Results

### Molecular diode characterization

**Figure 1:**
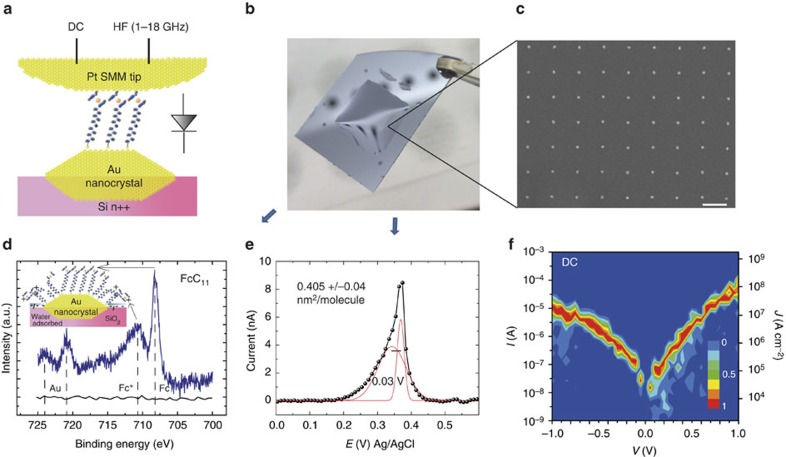
Description/characterization of the RF molecular rectifier. (**a**) Schematic representation of the molecular junction composed of a gold nanocrystal, Ferrocenyl undecanethiol (FcC_11_) molecules enabling rectification properties, and a Pt tip. Nanocrystals form an ohmic contact to a highly doped silicon substrate. The Pt tip is biased (through a bias-T) to both d.c. and HF (1–18 GHz) excitation simultaneously. (**b**) Picture of a 1 cm × 1 cm array of gold nanocrystals used for X-ray photoemission spectroscopy (XPS) or Cyclic Voltammetry measurements. The picture is taken just after dipping the sample into HF (for removal of the SiO_2_ covering dots), the gold nanoarray area being identified through an hydrophilic/hydrophobic contrast. (**c**) Gold nanodot array imaged by Scanning Electron Microscope (SEM). Scale bar, 200 nm. (**d**) XPS measurements for SAMs of FcC_11_ grafted on gold nanocrystals (∼1 billion dots fabricated by high-speed lithography) showing the presence of a Fe doublet related with ferrocene at 707.8 eV and 720.7 eV (Fe 2*p*_3/2_ and Fe 2*p*_1/2_, respectively), anda Fe doublet related to ferricenium at at 710.6 eV at 723.9 eV (2*p*_3/2_ and 2*p*_1/2_, respectively). The XPS signal for the bare Au nanoarray is also shown as a reference. Inset: schematic representation of the SAM with Ferricenium molecules located at dots borders due to the presence of a negatively charged silica. (**e**) Cyclic voltammetry measurements supports the presence of ferrocenyl molecules on the nanodots with a double peak at *E*=0.34 V and 0.37 V versus Ag/AgCl as a reference electrode in agreement with previous studies^15^. (**f**) 2D histogram (normalized to 1) showing the *I–V* (and *J–V*) curve from one hundred junctions on 20 nm gold nanoparticles. The voltage step is 0.1 V, and the 2D histogram is obtained by the contour plot function (Originlab). The applied load was 18 nN (see Supplementary Note 4 for a detailed discussion on the tip load).

**Figure 2:**
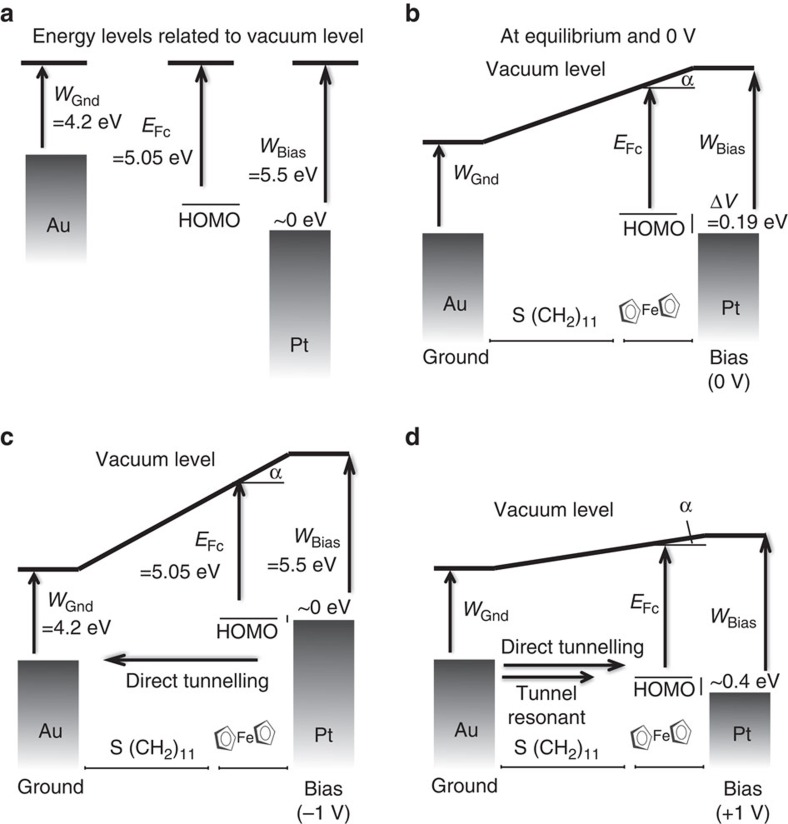
Sketch of the energetic level of the molecular device. (**a**) Sketch of the energetic level of the molecular device when negative (−1 V) is applied on the Pt tip (see details in Supplementary Information). (**b**) Same as **a** with 0 V applied on the tip. (**c**) Same as **a** with −1 V applied on the tip. (**d**) Same as **a** with +1 V applied on the tip. We considered a coupling parameter of 0.8 to the Fc from the gold atom (80% of the potential drop occurs in the alkyl chain) to calculate the energy shift of the HOMO levels in the junction (see Supplementary Fig. 6 for details).

Figure 1a shows the studied molecular diode junction composed of a gold nanoelectrode^54^,^55^,^56^,^57^, an archetype molecule for molecular diodes^13^,^14^,^15^,^16^ (ferrocenyl undecanethiol: FcC_11_SH), and a Pt tip. The tip curvature radius is large compared with the gold nanocrystal that defines the area of the molecular device (Supplementary Fig. 1), leading to a nearly ideal parallel-plate structure with few nm diameter^54^,^55^,^57^. In this study, the probe (Pt tip) is connected through a Bias-T to an interferometer for RF reflectometry measurements (iSMM^43^), and to an amperemeter (Resiscope) to get simultaneously the d.c. (Supplementary Fig. 2). Chemical analysis is performed on a large (1 cm × 1 cm) array with billions of the gold nanocrystals thiolated with FcC_11_SH (Fig. 1b), and they confirm the grafting of molecules on the nanocrystals. First, the X-ray photoelectron spectroscopy (XPS) spectrum (Fig. 1d) illustrates an excellent agreement with earlier studies. It shows a Fe doublet located at 707.8 eV and 720.7 eV (ref. 58), and a ferricenium peak at 710.6 eV (ref. 59) and 723.9 eV. While not always observed, for example, in large area self-assembled Fc monolayers on metal surfaces^58^, the presence of the ferricenium peak (Supplementary Fig. 3 for details) could be related to the silicon dioxide between dots in the presence of moisture. It may affect the stable charge state of Ferrocene molecules at gold nanocrystal sides (Fig. 2d, inset), dots borders representing ∼80% of the surface area (Supplementary Fig. 4). Second, electrochemical characterization by cyclic voltammetry (Fig. 1e) shows one broadened peak—more specifically two peaks—appearing at *E*=0.34 and 0.37 V (versus Ag/AgCl), in the range of expectation for Fc molecules^13^. Peak broadening or the presence of two peaks is usually related to molecular interactions in a densely packed SAM^13^,^15^,^60^ The average number of molecules per dot (∼860 considering the total dot surface—top plateau and sides—for these 20 nm-diameter, 3 nm-thick truncated cones^54^) corresponds to an average area per molecule of 0.405±0.04 nm^2^. It is assessed from the total charge measured by cyclic voltammograms at different sweep rates, and with consideration of an uncertainty on the averaged nanocrystal area (Supplementary Figs 4,5 and Supplementary Table 1). This averaged value is 10% larger (for example, molecule density slightly smaller) than for an ideal, fully packed, SAM with 0.37 nm^2^ per molecule^60^,^61^,^62^, but in the range of expectations for a dense SAM (Supplementary Note 1). Considering the sole top surface of the truncated cone (Supplementary Fig. 4), we can estimate to ∼150 the number of molecules contacted between the iSMM tip and the gold nanocrystal (molecules on dot sides do not contribute to the electron transport). Figure 1f shows a d.c. current–voltage, *I–V,* histogram obtained over 100 junctions from scans at different tip bias (bias applied on the tip)—see details in Supplementary Information.

The d.c. rectification ratio (*R*_d.c._=*|I(V=*1 V*)/I(V=−*1 V*)*|∼5) is in the range of usually reported values for many other types of molecule diodes^11^,^12^,^13^,^14^,^15^,^16^,^17^,^18^,^19^,^20^,^21^,^22^,^23^,^24^, albeit not the largest one reported for the same molecule^15^. The maximum dynamic conductance, *G*=*δI/δV*, calculated from the data in Fig. 1, is 360 μS at 1 V. It corresponds to a normalized conductance per molecule of 2.4 μS if we suppose that all 150 molecules are contacted, and if we neglect molecule–molecule interactions which are known to increase the total conductance of the junction compared with the sum of its parts^63^,^64^. Although large, it remains more than one order of magnitude below that of the quantum conductance (77.5 μS). With this in mind, we propose a simple energy band diagram^9^ of the molecular device in Fig. 2. The energy level for the HOMO is estimated from cyclic voltammetry to be −5.05±0.05 eV relative to vacuum (Supplementary Fig. 6 and Supplementary Note 2). In this situation, a higher conductance is expected for positive bias on the Pt electrode when the HOMO levels of the Fc group are pulled down to lie in the energy window defined by the Fermi energy levels of the electrodes (resonant tunnelling). Note that the direction of rectification is opposite to that of other published results with same/similar molecule^16^,^17^,^18^,^19^,^20^. This feature can be mainly attributed to the large metal work function of the Pt top electrode (Supplementary Table 2 and Supplementary Note 3) and to the work function difference between the two electrodes (Au and Pt), which favour a rectification direction in the opposite direction of the one attributed to the energetics of the Fc molecule in the junction.

The dynamic conductance (up to 0.36 mS at 1 V) is suitable for high frequency operation as discussed later in the paper. The capacitance will be discussed together with RF measurements.

### RF-molecular diodes

**Figure 3:**
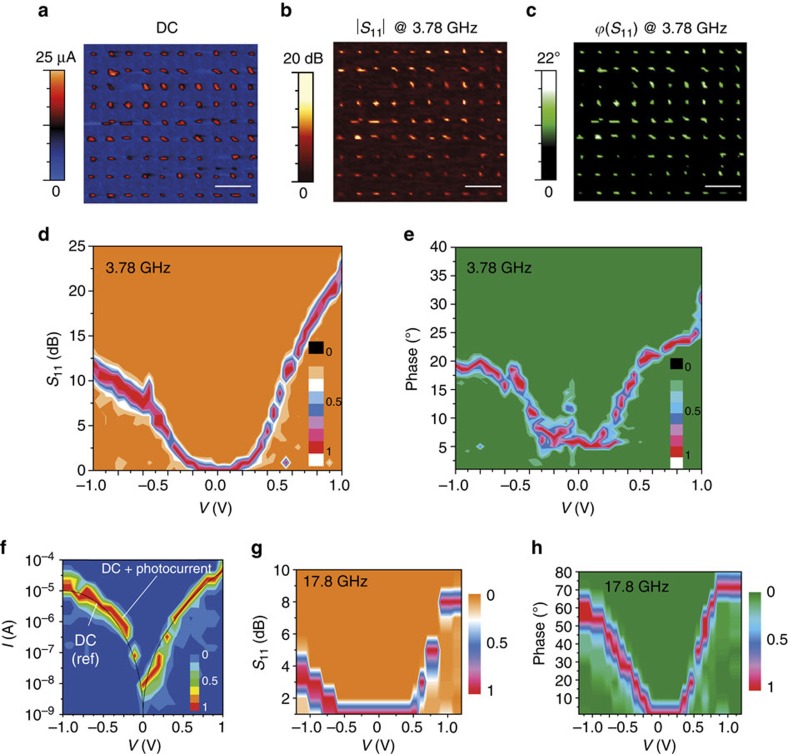
Demonstration of a molecular rectifier at 3.78 and 17.8 GHz. (**a**–**c**) iSMM images at 0.8 V d.c. and 3.78 GHz measured simultaneously by the amperometer (Resiscope) for the d.c. (**a**) and vectorial network analyser (VNA) for amplitude (**b**) and phase (**c**) *S*_11_ parameters. Scale bars, 500 nm. (**d**,**e**) 2D |*S*_11_| histogram (normalized to one) versus tip bias (*V*) and *ϕ*(*S*_11_)–*V* 2D histograms (normalized to one) generated from one hundred molecular rectifier junctions. The voltage step was 0.05 V and the contour plot generated automatically (Originlab). The applied load was 18 nN. (**f**) 2D d.c. *I–V* histogram from one hundred of ferrocenyl undecanethiol gold nanojunctions with a 17.8 GHz RF input signal. The d.c. reference current (solid line) when no RF input signal was added is shown for comparison. It was obtained from the average *I–V* from a 2D histogram, without RF power. The voltage step was 0.1 V and the contour plot generated automatically (Originlab). (**g**,**h**) 2D |*S*_11_| versus voltage curve from one hundred of ferrocenyl undecanethiol gold nanojunctions at 17.8 GHz and related *ϕ*(*S*_11_) versus *V* curve. The voltage step was 0.1 V and the contour plot generated automatically (Originlab).

**Figure 4:**
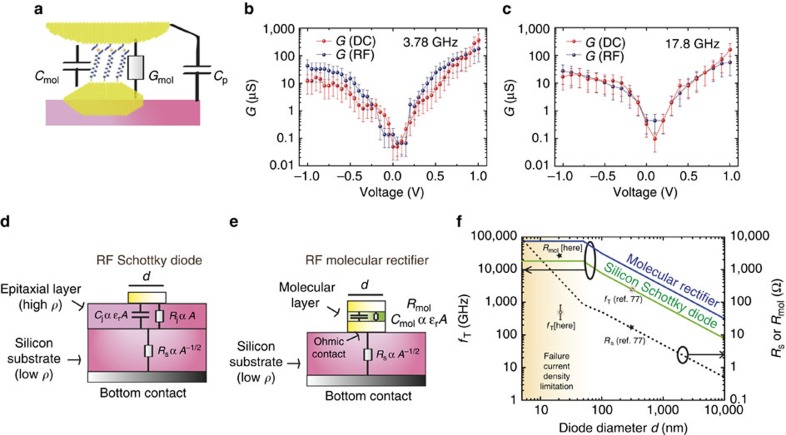
Perspectives for RF-molecular rectifiers. (**a**) Equivalent circuit representation of the device. Molecules can be decomposed as a conductance in parallel with a capacitance (*G*_mol_ and *C*_mol_). Also, a fringe capacitance (*C*_p_) between the iSMM tip and the sample has to be considered. (**b**) Conductance *G*_mol_ estimated from both the d.c. measurement (*δI*/*δV*) —red curve—(technically obtained after multi-exponential fit with 200 points of the d.c. *I*–*V* curve: 21 points), and from *S*_11_ parameters (equation1)—blue curve—(see Supplementary Information, section 8 for fitting details). The error bar in log scale is considered to be the same as that of full width half maximum in current histograms. (**c**) Similar curves as in **b** at 17.8 GHz. The error bar in log scale is considered to be the same as that of full width half maximum in current histograms. (**d**) Schematic cross section of the RF Schottky diode architecture. The high resistivity epitaxial layer is thin (few nm) so as to to tune *R*_j_ up to *R*_s_, but not too thin to avoid a large *C*_j_. The substrate is highly doped (resistivity *ρ*_s_=0.001 Ω cm). Its resistance scales as *A*^1/2^ where *A* is the junction area. (**e**) Schematic cross section of the proposed RF molecular rectifier. The molecular layer plays the role of the diode with a small dielectric constant *ρ*_*r*_. Similar to Schottky diodes, the molecular diode is connected to a highly doped silicon substrate. (**f**) Graph illustrating the theoretical (ideal) *f*_T_ and resistance (*R*_*s*_ or *R*_mol_) for both the RF-molecular rectifier and the RF-Schottky diode architectures shown in **d**,**e**. The dash curve corresponds to *R*_*s*_*=ρ*_*s*_/2*d* with *ρ*_*s*_=1 mΩ·cm (ref. 78). *C*_*j*_*/A=*6.2 μF cm^−2^ from refs 77, 78 and *C*_mol_/*A*=0.9–1.4 μF cm^−2^ based on a dielectric constant of 2–3 for the monolayer and a monolayer thickness of 1.9 nm (the length of the molecule). A current density failure limitation of 3 × 10^8^ A cm^−2^ from refs 79, 80, induces a saturation of *f*_T_ in the graph. Measured *R*_mol_ at +1 V and estimated *f*_T_ are also indicated. The error bar is related to the conductance dispersion from 2D *I–V* histograms.

Figure 3a–c shows the iSMM images of d.c., RF reflection coefficient *S*_11_ amplitude (*|S*_11_*|*) and phase (*ϕS*_11_) collected simultaneously at 0.8 V and 3.78 GHz. *S*_11_ is the ratio of reflected RF signal power over the incident RF signal power. *|S*_11_*|−V* and *ϕS*_11_*–V* two-dimensional (2D) histograms are shown in Fig. 3d,e. A molecular diode behaviour is clearly observed with a rectification ratio in *|S*_11_*|* at ±1 V, *R*_RF_=*|S*_11_*|*_*V=*1 V_−*|S*_11_*|*_*V=−*1 V_ of 12 dB, whereas for a symmetric molecular junction (alkyl chains of similar length), no rectification was observed (Supplementary Figs 7,8). The molecular diode device is still operating at a frequency of 17.8 GHz, the upper limit of our experimental setup (see Fig. 3f–h for the *I–V*, *|S*_11_*|*–*V* and *ϕ*(*S*_11_)–*V* at 17.8 GHz), although we notice a reduced rectification ratio in |*S*_11_| at ±1 V of 4 dB. We do not notice any increase in the d.c. induced by the RF signal (photocurrent). Fig. 3f shows the measured d.c. *I–V* with/without RF applied on the tip (black solid line), confirming that no photocurrent is measured, even at 17.8 GHz. This result is not surprising at room temperature as the RF excitation corresponds to an energy of 73.6 μeV, much smaller than the thermal energy (25 meV). Also, the estimated absorbed power (<1 μW) is typically lower than Joules power (up to few μW). Eventually, we could have expected a RF-modulation of interfacial dipoles^51^, observable either on the current (through the so-called molecular gating^57^,^65^,^66^) or on *S*_11_ via a capacitance change due to a polarization effect^35^,^51^,^67^. These effects are not observed due to the weak dipoles and the presence of a fringe capacitance, which hides the effects. The rectifying behaviour on *S*_11_ can be related to the d.c. properties. Considering the RC equivalent circuit shown in Fig. 4a (the dynamic conductance *G* must be derived from *δI/δV* of the d.c. current–voltage curve or from the RF measurements as detailed below, the capacitance is *C=C*_mol_*+C*_p_ with *C*_mol_ the capacitance of the molecular junction and *C*_p_ the fringe capacitance between the tip and substrate), the RF reflection coefficient *S*_11_ of the device under test is related to the impedance of the measured device, *Y=G+jCω* by refs 43, 45:


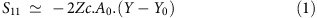


where the parameter *A*_0_ takes into account losses, gain and shifts due to cables, passive and active elements and *Y*_0_ is the admittance for which fully destructive interference occurs (*S*_11_=0), and *Z*c is the characteristic impedance of 50 Ω. To extract the RF conductance, *G*_RF_, from the *S*_11_ measurements (Fig. 3), we need a calibration protocol (Supplementary Methods 1 and Supplementary Fig. 9) to determine the parameter *A*_0_, *Y*_0_*=G*_0_*+jC*_0_*ω*, and the capacitance *C*. From this calibration protocol, we get *A*_0_=(61 dB;−153°) at 3.78 GHz and (52 dB; −90°) at 17.8 GHz, *G*_0_≈0, *C*_0_=300 aF, and *C*=110 aF. Using these values in equation 1, *G*_RF_*–V* curves (Fig. 4b,c) are extracted from *S*_11_ data (Fig. 3) at 3.78 and 17.8 GHz, and compared with *G*_d.c._*–V* curves. We observe a reasonable agreement both at 3.78 GHz and 17.8 GHz. This means that this molecular diode works without significant perturbation up to 17 GHz. Although our experimental setup system is limited to 18 GHz, we can estimate the cut-off frequency *G*_max_*/(*2*πC)=*520 GHz, where *G*_max_ is the maximum dynamic conductance (0.36 mS in Fig. 4b at 1 V) and *C=C*_mol_*+C*_p_∼110 aF as discussed above. Note that *C∼C*_p_ as *C*_mol_ is expected to be of only few aF from geometric dimensions and a dielectric constant of 2–3 for the organic monolayer^37^.

## Discussion

The present nanometre-scale molecular diodes offer a current density increased by more than nine orders of magnitude compared with that of micrometre-scale molecular diodes with the same molecule^15^,^16^, allowing RF operation (see Supplementary Table 2 for a direct comparison of current densities for various architectures^16^,^17^,^18^,^19^,^20^). As mentioned in the introduction, this difference can be mainly explained by the fraction of molecules connected due to a lower roughness in nanometric junctions^29^,^30^,^31^,^32^,^33^. In our system, gold nanocrystals have an atomically flat top surface^57^. Also it is possible that the *π*-orbitals of the ferrocene molecule overlap with the Pt spill-over electron density, resulting in a large contact conductance, even without chemical contact^6^,^9^,^68^,^69^. If considering an upper conductance limit of *G*_0_ for the dynamic conductance per molecule, then at least 5% of the molecules contribute to the electronic transport (in other words, probably a large fraction of the molecules are connected). Nonetheless, we also believe that the molecular packing plays a role as the alkyl chains, that govern the coupling strength between the Fc molecules and the bottom electrode^9^, take a small space compared with that of Fc heads^15^,^16^,^60^. Cyclic voltammetry experiments (Fig. 1e and Supplementary Note 1) suggested that the present monolayers on the gold nanocrystals are 10% less dense than for a fully packed monolayer. Weaker van der Waals forces between alkyl chains should reduce the distance between ferrocene molecules and gold nanocrystals^70^ (for example, increase the tunnelling rate), reduce the tunnelling decay ratio^16^, and weaken the effect of tip load^57^ which we observed experimentally (Supplementary Figs 10–12 and Supplementary Note 4 for a discussion on the effect of tip load^71^ and molecule). Finally, we cannot exclude an impact from the metal atom oxidation state on the conductance^72^ (for example, here the presence of Ferricenium molecules seen on XPS spectra). However, if located on nanocrystals sides (Fig. 1d, inset), ferricenium molecules should barely affect the electronic properties. Moreover, Lee and coworkers^16^ suggested that electron transport through ferricenium is slightly lower (a factor≈1.5) than through ferrocene, thus the possible presence of few ferricenium ions on the top of the nanodot is not drastic.

In the present experiments, the change of frequency from 3.78 GHz to 17.8 GHz already points out the appearance of the capacitive contribution *|C−C*_0_*|ω* as a plateau in *|S*_11_*|* at low bias. Theoretical predictions suggest that an additional inductive contribution^4^ (inertia related to carriers effective mass) or oscillation in *S*_11_ due ferrocene spinning^73^ should be observed in the THz range. Recently, Tan *et al*.^74^ have shown that tunnelling charge transfer plasmon modes can operate through molecules at hundreds of THz, suggesting that there might not be physical limits to operate molecular rectifiers in the THz gap. However, in the hypothesis of incoherent tunnelling, the limiting speed for capture/emission of a charge by a redox molecule^13^ or more generally from a trapping site under large electric field^75^ still remains to be explored. The present studied frequency range should already be ideal for routine measurement of the shot noise from *S*_11_ parameter^76^, another degree of freedom for getting insights in the operation mechanisms of molecular diodes or other molecular devices as it is directly related to the transmission function in the Landauer formalism.

We now compare the RF-silicon Schottky and RF-molecular diodes architectures (Fig. 4d,e). Schottky diodes are a key component in RF mixers, and are used in wifi or mobiles phones. The cut-off frequency *f*_T_ is typically the figure of merit, not only for the maximum frequency operation, but also for optimized power consumption in practical applications^77^,^78^. To optimize the conductance, RF-Schottky diodes are biased in the non-linear regime (induced by a depletion layer at the metal/semiconductor interface) just below the linear regime governed by the spreading resistance in the substrate (*R*_S_). A thin undoped epitaxial layer (few nms) enables to keep the highly doped substrate as close as possible to the interface while not getting a too large junction capacitance *C*_J_ (a typical value^77^ for *C*_J_*/A* is 6.2 μF cm^−2^). *f*_T_ is usually considered as 1/*(*2*πR*_S_*C*_J_)^77^,^78^. Whereas *C*_J_ scales as the junction area *A*, *R*_S_ scales as *A*^−1/2^ because the substrate thickness is large compared with that of the junction diameter *d*^78^. This has led to smaller and smaller RF-Schottky diodes with *f*_T_ scaling as *A*^−1/2^ (green line Fig. 4f). A *f*_T_ of few THz was demonstrated for a 250 nm diameter diode^77^, close to the theoretical prediction (Fig. 4f). However, further scaling these diodes should induce unsustainable large current densities (the typical upper range of failure current limitation is ∼3 × 10^8^ A cm^−2^)^79^,^80^, unless a small oxide layer at the contact interface increases the resistance^81^. A maximum current density imposes *R*_*S*_ to scale with *A*, leading to a saturation of *f*_T_ at *d* below 50 nm (Fig. 4f). In the RF-molecular diode architecture (Fig. 4f), organic molecules replace the undoped semiconductor epitaxial layer. The theoretical cut-off frequency limit is given by 1/*(*2*πR*_S_·*C*_mol_), resulting in a gain of *C*_J_*/C*_mol_∼5.6–8.8 when compared with Schottky diodes (typical *C*_J_ is considered^77^) thanks to a small dielectric constant of 2–3 for organic monolayers^37^ (blue curve in Fig. 4f). The failure issue at high current density remains valid in the molecular rectifier architecture. However, in the present experiment, as *G* is lower than *1/R*_S_ in the ±1 V biasing range (current governed by molecules conductance), such limit is not reached with these 20 nm-diameter gold nanocrystal electrodes (although not so far). The comparison of the experimental values at 1 V for *R*_mol_*=1/G* and *f*_T_ with theoretical optima suggests that the actual limiting factor is not the molecule conductance, but rather fringe capacitances that degrade *f*_T_ by about two orders of magnitude. Still, improvement of the device conductance (optimization of the conductance per molecule^24^ and rectification ratio (for example, a larger rectification ratio has been reported with the same molecule using other electrodes^15^) by tuning the metal work function or molecular organization could enable reducing the optimum operation voltage while keeping the same *f*_*T*_. Finally, we stress that for practical applications, nano silicon Schottky diodes are assembled into arrays so as to benefit from the high performances related to small dimensions while keeping *R*_*s*_ to few Ohms^77^ (close to the 50 Ω impedance). By analogy, standalone optimal RF-molecular diodes could be obtained in the future based on the present array of molecular nanodiodes covered by a top contact. Such top contact electrode could be, for example, graphene ribbons thanks to their RF compatibility^82^, small contact resistance^83^, and rigidity so as to decrease fringe capacitances between dots.

To conclude, we demonstrate a high-frequency molecular rectifier composed of self-assembled small molecules. It brings new hopes and perspectives for a chemistry-based electronics. There is a tradeoff between the conductance, the function (here rectification) and capacitances. The present configuration was optimized to demonstrate a 17 GHz molecular rectifier with a 520 GHz cut-off frequency: The ∼150 molecules sandwiched between a gold nanocrystal and a Pt tip electrode enables to get a ∼0.36 mS dynamic conductance while keeping a small capacitance in the 100 aF range. The sole reduction of the fringe capacitance to 50 aF by using an ultra-sharp tip^45^ or by surface functionalization between nanocrystals would enable to operate these diodes in the THz range where new functionalities are theoretically predicted, for example, a huge increase of the dynamic conductance of the molecular devices^84^,^85^.

## Methods

### Gold nanodot electrode fabrication

The fabrication of the gold nanocrystal arrays has been described elsewhere^54^,^86^. For e-beam lithography, we used an EBPG 5000 Plus (Vistec Lithography). Silicon (100) substrates of 0.001 Ω cm resistivity. They were exposed to Ultraviolet-ozone and the native oxide removed by HF at 1% before resist (PMMA 950 K, diluted 3:5 with anisole, 45 nm thick) spin-coating.

The e-beam acceleration voltage was 100 keV. The dose per dot corresponds to 3–4 fC (ref. 86). Just prior evaporation, the native oxide was removed with dilute HF solution to allow good electrical contact with the substrate. Similarly, we stress that it is important not to use any adhesion layer such as Ti as the diffusion of gold in the silicon substrate is required to get the Au nanocrystal structure^58^ used.

After thermal annealing at 260 °C during 2 h under N_2_ atmosphere, we get Single crystal Au nanodots (Supplementary Fig. 1). During this process a thin layer of SiO_2_ covers the dots^54^. It is etched by HF (1%, 1 mn) before SAM grafting.

### Self-assembled monolayers

For the FcC_11_SH monolayer, we used 11-ferrocenyl-1-undecanethiol of 95% purity from Aldrich. The gold nanocrystals were exposed to a solution of 20% dichloromethane and 80% ethanol (VLSI grade from Carlo Erba) during typically 24 h in a glovebox in the darkness. The treated substrates were rinsed in ethanol and further cleaned with gentle ultrasonication (20% power, 80 kHz) in chloroform (99% from Carlo Erba) during 1 min. The gentle ultrasonication was required to avoid pollution of the tip from adsorbed molecules on silica. In the future, ultrasonication may not be necessary if a monolayer of silane molecules is grafted between dots to prevent non-specific adsorption. Similarly, recent studies performed by Jian *et al*.^87^ suggest that a fully packed SAM could be obtained after an additional purification step to avoid for example the presence of disulphides. We have not done such extra purification step in the present study. It could be an interesting perspective to evaluate its impact on the performance of RF molecular diodes.

### XPS measurements

XPS measurements have been performed using a monochromatic Al (Kα) X-ray source (1486.6 eV) and an analyser pass energy of 12 eV. The spectrometer was from Physical Electronics (5,600).

The analyser acceptance angle, the detection angle and the analysed area were set to 14°, 45° and 400 μm, respectively.

### iSMM and resiscope set-up

The interferometric scanning microwave microscope (iSMM), recently developed at IEMN^43^, is an adjustable interferometer analogous to a Mach-Zehnder configuration. It consists on a coaxial power divider and two coaxial hybrid couplers associated to an active variable attenuator (Supplementary Fig. 2). The VNA source delivers the incident signal, *a*_1_, that is split into two parts. One part of the signal, used as a reference, is adjusted in magnitude by the variable attenuator in order to adjust the interference; the second part feeds the AFM tip through a coupler and is then reflected back by the device under test (DUT). Both signals are combined inside the second coupler and then the signal, *a*_3_, is amplified and analysed the VNA receiver.

This iSMM is connected with an amperometer by a bias-T that allows electrical characterization of the d.c. simultaneously (Supplementary Fig. 2). As an independent measurement, the ResiScope (log amplifier) measures the sample resistance through the High Performance Amplifier (HPA).

The iSMM measurements were done with Agilent 5,600 AFM combined with an Agilent PNA series network analyser. The cantilevers from Rocky Mountain Nanotechnology are Pt solid wires glued to an alumina chip to enable compatibility with high-frequency measurements. The tip used (RMN 12Pt300A) were 300 μm-long, 60 μm-wide and a shank length of 80 μm with a tip curvature radius of about 20 nm. In practice, we found that the curvature radius for these Pt wire tips was about 80 nm (ref 45), so we had to increase the interdot distance to 200 nm. The spring constant is given to be about 0.8 N m^−1^ enabled to control force and set it to 18 nN (see Supplementary Note 4 for a discussion on the tip load). Because the dots' dimensions were larger in that study when compared with refs 55, 57, the influence of the force was reduced (smaller force per surface unit) in the nN range. Images were acquired with a sweep frequency of 0.1 lines per second (go and back) and 512 pixels per line. For an image of 15 μm^2^ it corresponds to a scan rate of 3.12 μm s^−1^. Given an interspacing between nano-junctions of 250 nm, we can measure 3,600 junctions in 1 h 22 min at a given d.c. bias. The voltage applied on the substrate by the Resiscope software, but for convenience and comparison with other published data, all the voltage values are reported in the paper figures as applied on the tip.

### Cyclic voltammetry

Supplementary Fig. 4a shows the electrochemical cell used in this study. The 0.5 ml container is filled with NaClO_4_ (0.1 M in water) as the electrolyte (Supplementary Fig. 13). The cell is connected with the Solartron ModuLab potentiostat by the three typical electrodes. Typical Ag/AgCl and Pt wire electrodes were used as the reference and counter electrodes, respectively. The working electrode is the gold nanocrystal array on the silicon substrate.

Before the experimental measurements, the electrochemical cell was cleaned with ethanol and DI water. ‘Test' sweeps between −0.1 and 0.6 V with a highly doped silicon substrate (without dots) are measured to confirm that there is no peak due to contamination. Cyclic voltammetry (stable under several cycles: Supplementary Fig. 5) proves the presence of ferrocene-thiol electroactive molecules and allows their quantification.

### Image treatment

Images were treated with Origin C programme with two functions. The first function applies a threshold to remove the background noise. Then, second function obtains the maximum per dot by checking the nearest neighbours. Due to temperature fluctuation in the interferometer, or to the mechanical relaxation in the cables, both active and passive elements on the interferometer may undergo a particular drift. The interference set at the beginning of the experiment may move as we see in the Supplementary Fig. 14a and it causes changes on our measures as noticed in Supplementary Fig. 14b (change of the background colour). There are two contributions to this drift: One is related to the increment of the reflected signal when the interference moves from the selected frequency. It is solved by subtracting the ground level measured on the native SiO_2_. The second contribution comes from the fact that the reflected signal decays exponentially when the interference moves away the selected frequency. The procedure followed to overcome this limitation during the measurements of the *S*_11_ parameters versus voltage has been to reduce the image size to about 100 nanojunctions and normalize the data to those obtained on the flat zone. This procedure has been validated for inorganic nanocapacitors using an on chip calkit^45^. Therefore, the complete curves with 41 images at different bias can be done in only 3 h (Supplementary Fig. 15), which significantly reduced drift issues.

### Data availability

All relevant data are available from the authors on request.

## Additional information

**How to cite this article:** Trasobares, J. *et al*. A 17 GHz molecular rectifier. *Nat. Commun.* 7:12850 doi: 10.1038/ncomms12850 (2016).

## Supplementary Material

Supplementary Information Supplementary Figures 1-15, Supplementary Tables 1-2, Supplementary Notes 1-4, Supplementary Methods and Supplementary References

Peer Review File
