# Supplementary material for: A 17 GHz molecular rectifier
Source: Nat Commun. 2016 Oct 3;7:12850. doi: 10.1038/ncomms12850 (PMC5059435; doi:10.1038/ncomms12850)
Supplement: Supplementary Information — Supplementary Figures 1-15, Supplementary Tables 1-2, Supplementary Notes 1-4, Supplementary Methods and Supplementary References [file ncomms12850-s1.pdf]

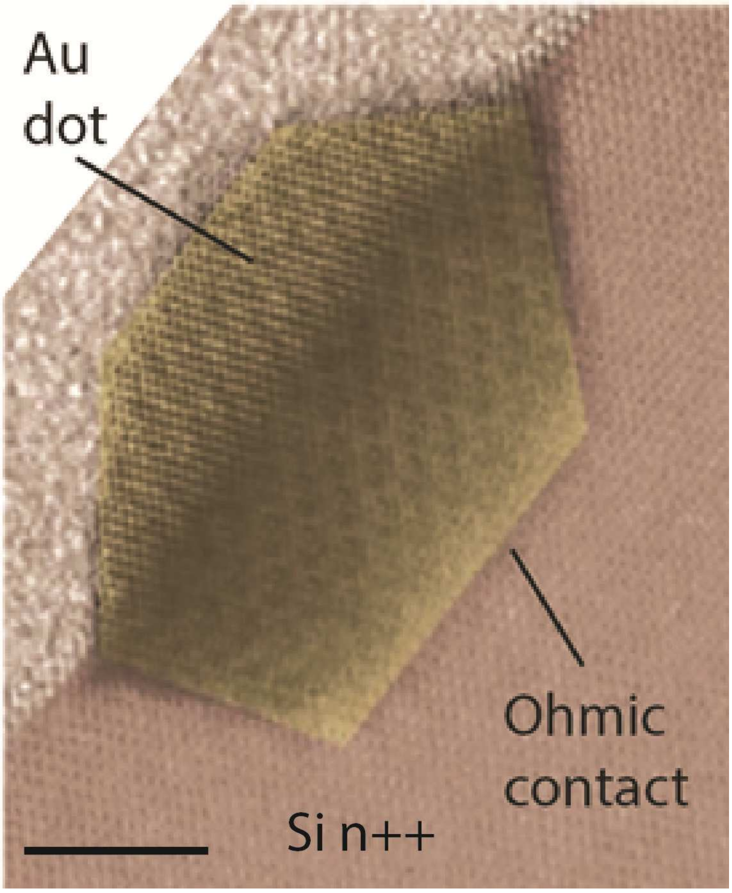

Supplementary Figure 1. Gold nanodot electrode fabrication Coloured TEM images of an Au nanodot (adapted from Ref.1). Scale bar is 5 nm.

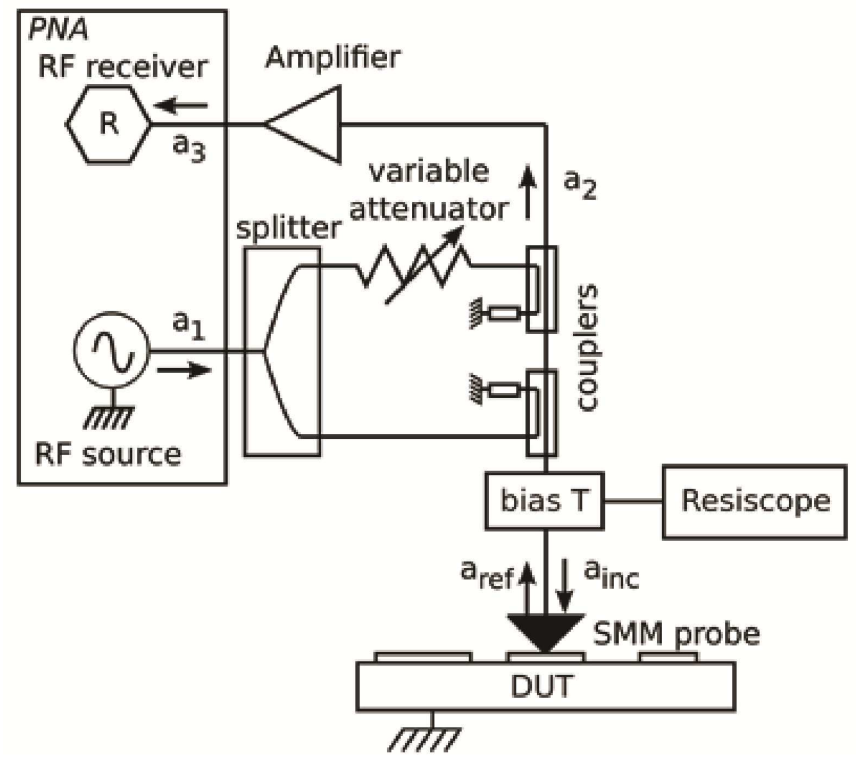

Supplementary Figure 2. Experimental setup composed of an interferometer[2] and log current amplifier (resiscope[3])

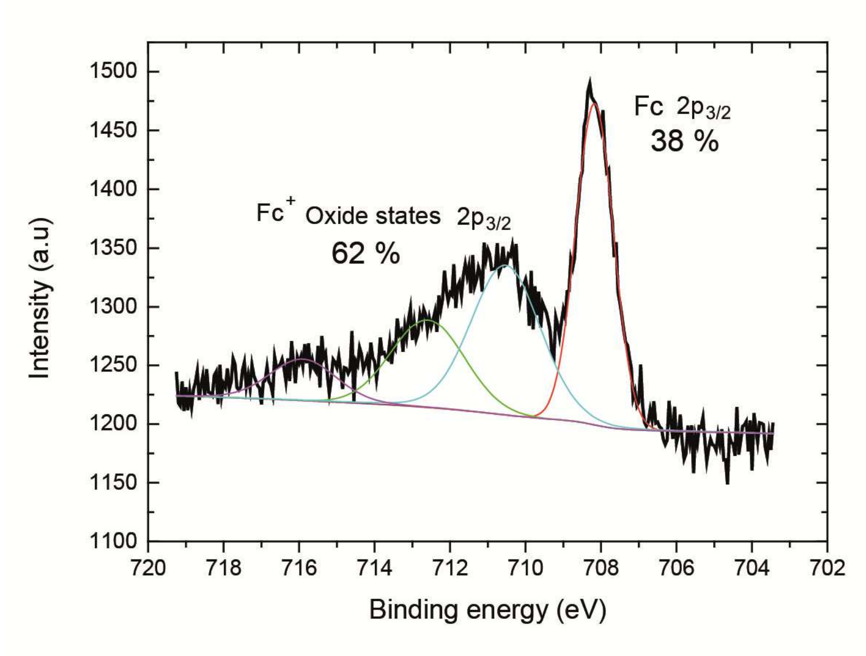

Supplementary Figure 3. XPS spectrum (zoom on Fig.1d) used to evaluate the relative fractions of Ferrocene (38%) and Ferricenium=62% from respective areas of each Gaussian fit. The estimation for the area on top (21%) and sides (79%) could match with ferricenium molecules mainly located on dots sides. When compared to ref[4], the FWHM is halved for both peaks, but the ratio of FWHM for both peaks is similar (e.g. broad peak for ferricenium). Background was substracted by Shirley algorithm[5].

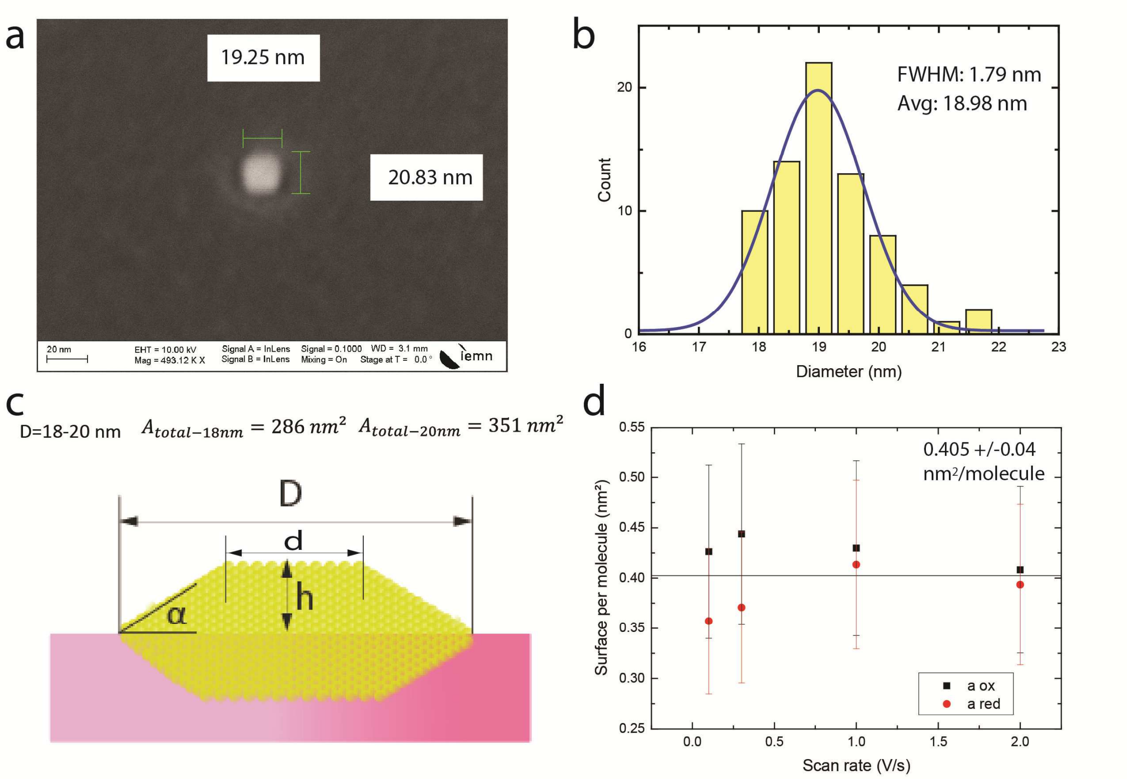

Supplementary Figure 4 a Example of SEM image for a gold nanocrystal. **b** Based on the estimation of nanocrystals diameters from 80 images as **a**, we obtained the statistical repartition of 19 nm +/- 1 nm. **c** Schematic cross section representation of a nanocrystal from Fig.S1 used to estimate the total area of a nanocrystal. D is the total diameter, d is the diameter on top of the dot, α was considered to be 30° and h=3 nm. The area was considered with the following formula: $\pi/4*(D-2*h/\tan\alpha)^2+H*(D-h/\tan\alpha)*h/\sin\alpha$, the first term corresponding to the area on top and the second term to the area on sides. **d** Extracted surface per molecule for each scan rate (Supplementary Figure 5). The error bar is related to the uncertainty on dot diameter estimated in **b**. The averaged area per molecule is 0.405 +/- 0.04 nm^2/molecule.

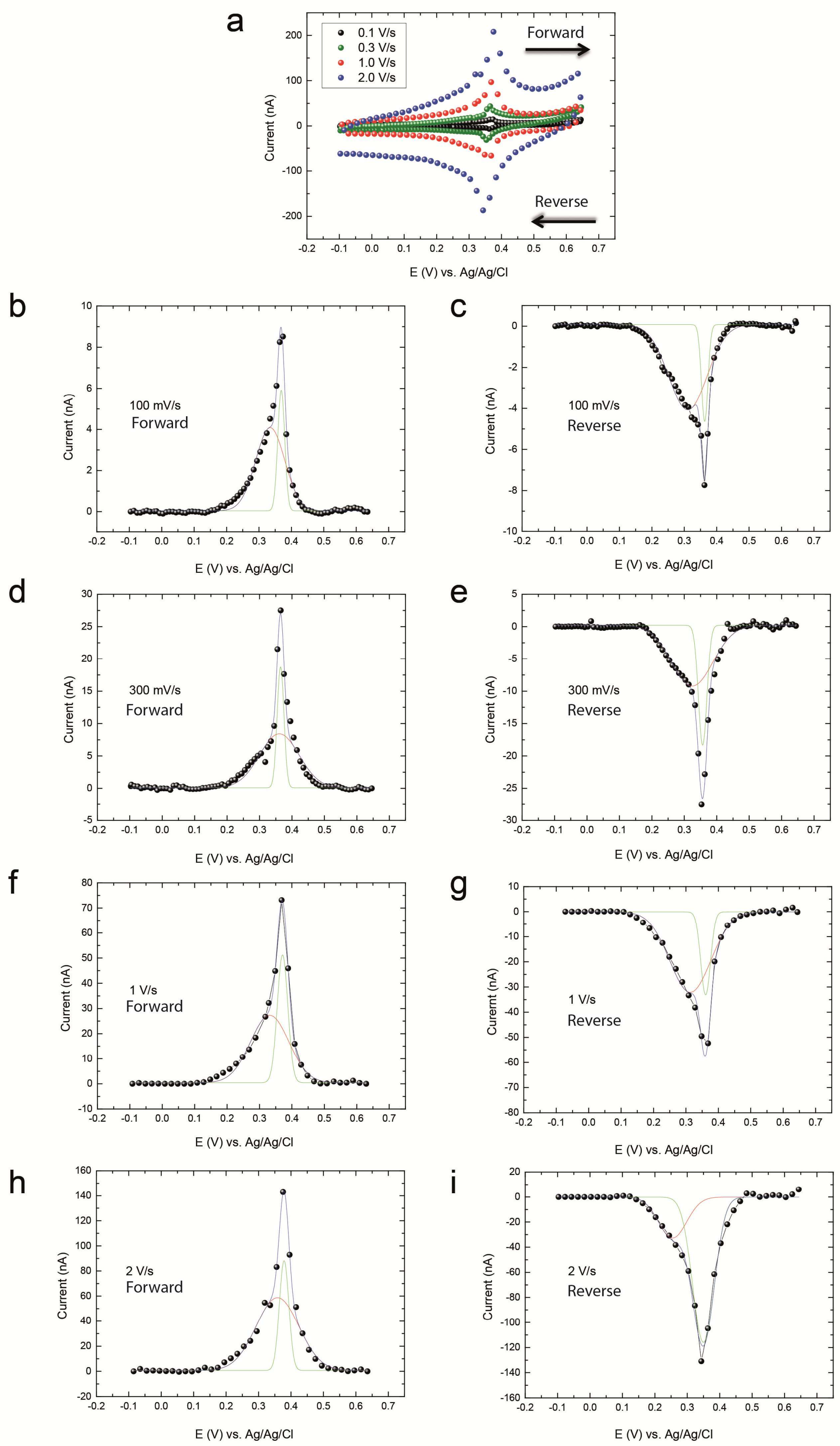

Supplementary Figure 5 a Raw datas for the cyclic voltammogram corresponding to Fig.1e, and voltammograms at different sweep rates (forward and reverse). **b-j** Cyclic voltammograms after removal of the base line (capacitive contribution) at different sweep rates (left:forward; right:reverse).

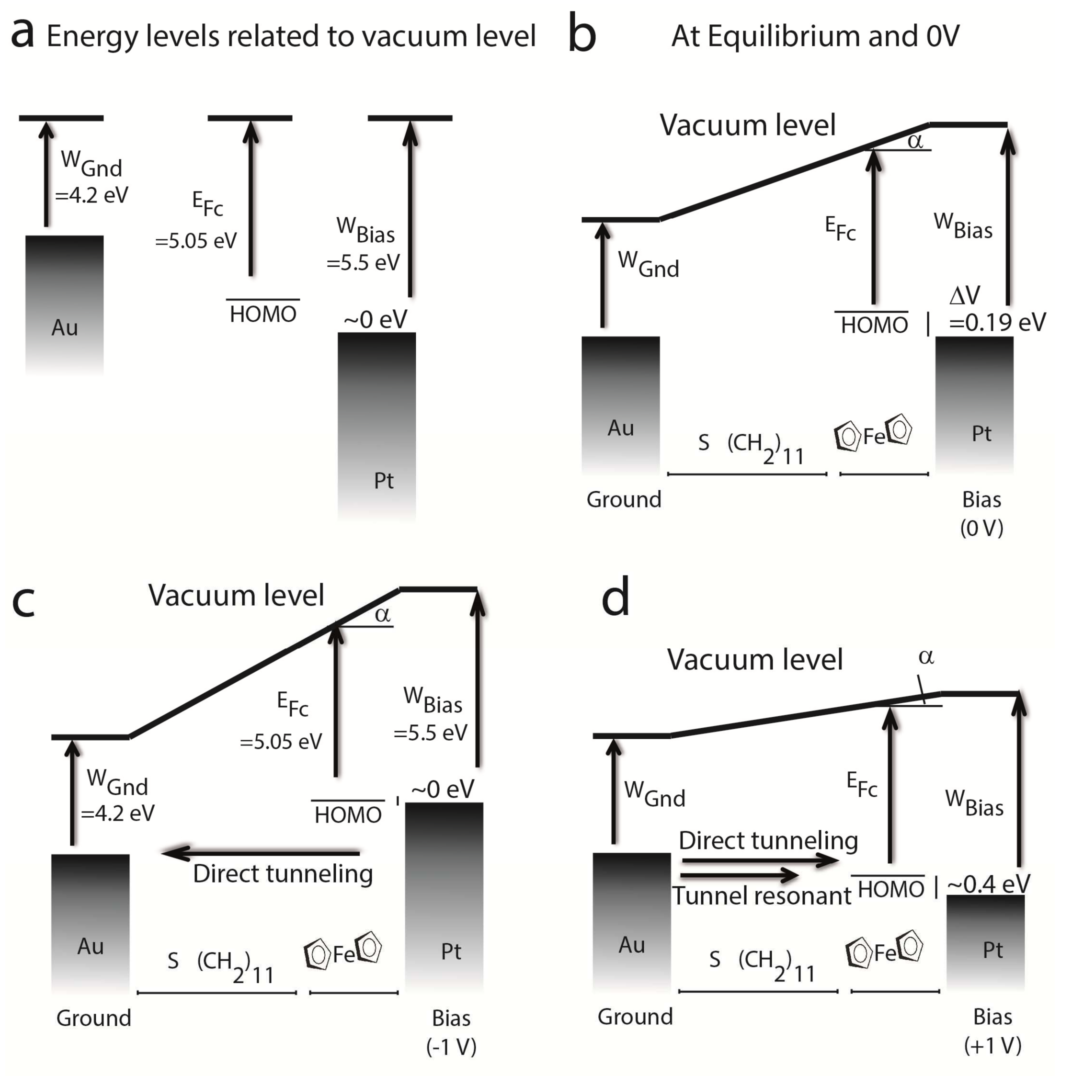

Supplementary Figure 6 Construction of the energy band diagram for the molecular rectifier a Energy levels related to vacuum for the electrodes (work functions) and for the molecules (E_{HOMO}, cf Supplementary Table 1 and Supplementary Notes 2). **b** When electrodes contact the molecule, at thermodynamic equilibrium and V=0V, Fermi levels of both electrodes are aligned. ΔE_{wf} represents the difference of metal work function. $\alpha\Delta E_{wf}$ represents the difference of energy between E_{HOMO} and the gold electrode, that has to be taken into account due to the difference in electrodes metal work functions. Parameter α=0.8 was considered as a typical value between 0.7[6] and 0.9[7]. In ref.6, a GaIn electrode with a Ga_2O_3 oxide layer was used, and in 7, a Pt electrode was used. As parameter α can be defined as $V_r^2/(V_r^2+V_l^2)$ where V_l and V_r represent the energies of coupling to the left and right electrodes respectively, it is likely that the coupling between the right electrode and the Fc molecules is slightly reduced in the case of GaIn due to the presence of Ga_2O_3. By appling a bias on the Pt tip (-1V and +1V), the corresponding energy band diagrams are shown in **c** and **d**.

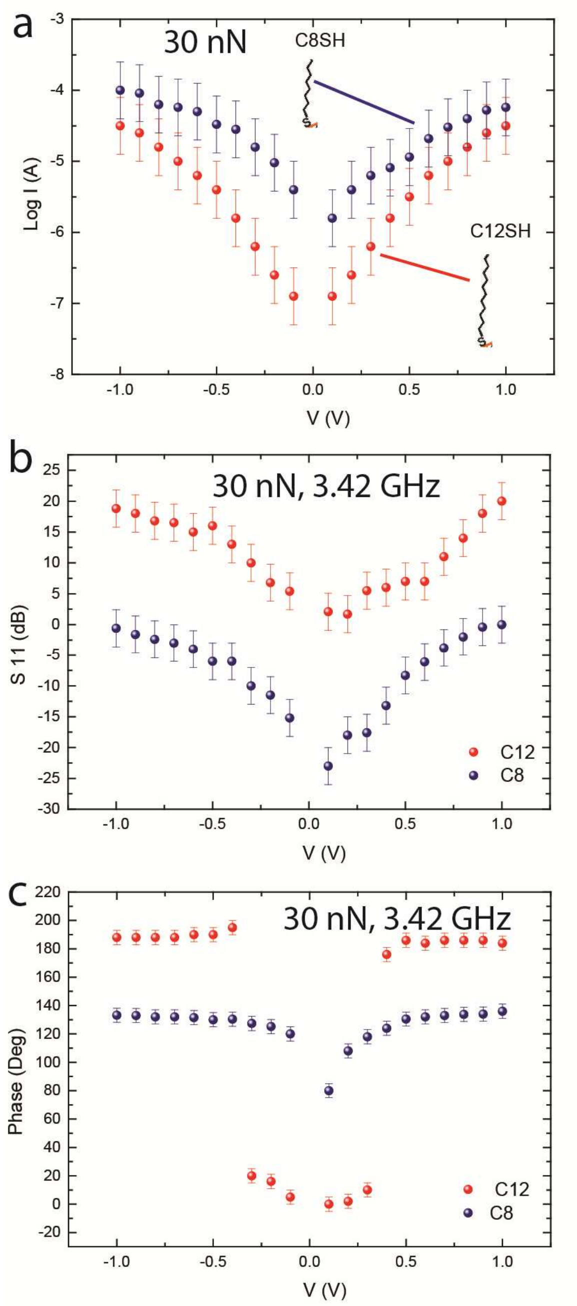

Supplementary Figure 7 a DC current (Log scale) for SAMs composed of C_8 and C_{12} molecules. **b** Related $|S_{11}|$. **c** Related S_{11} phase. In all curves, the dot corresponds to the maximum in the log normal distribution (from 100 dots) and the error bar is related to the standard deviation. The applied force was 30 nN. It is larger than the force applied on the FcC_{11} because no variation of S_{11}-V could be observed at 18 nN for C_{12} molecules (pure capacitive regime).

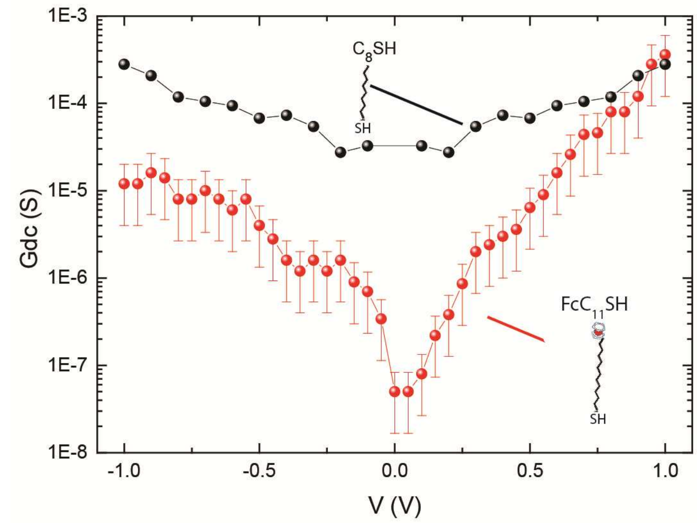

Supplementary Figure 8 Dynamic conductance obtained from I-V curves for the $FcC_{11}SH$ (at 18 nN) and C_8SH (at 30 nN) molecules

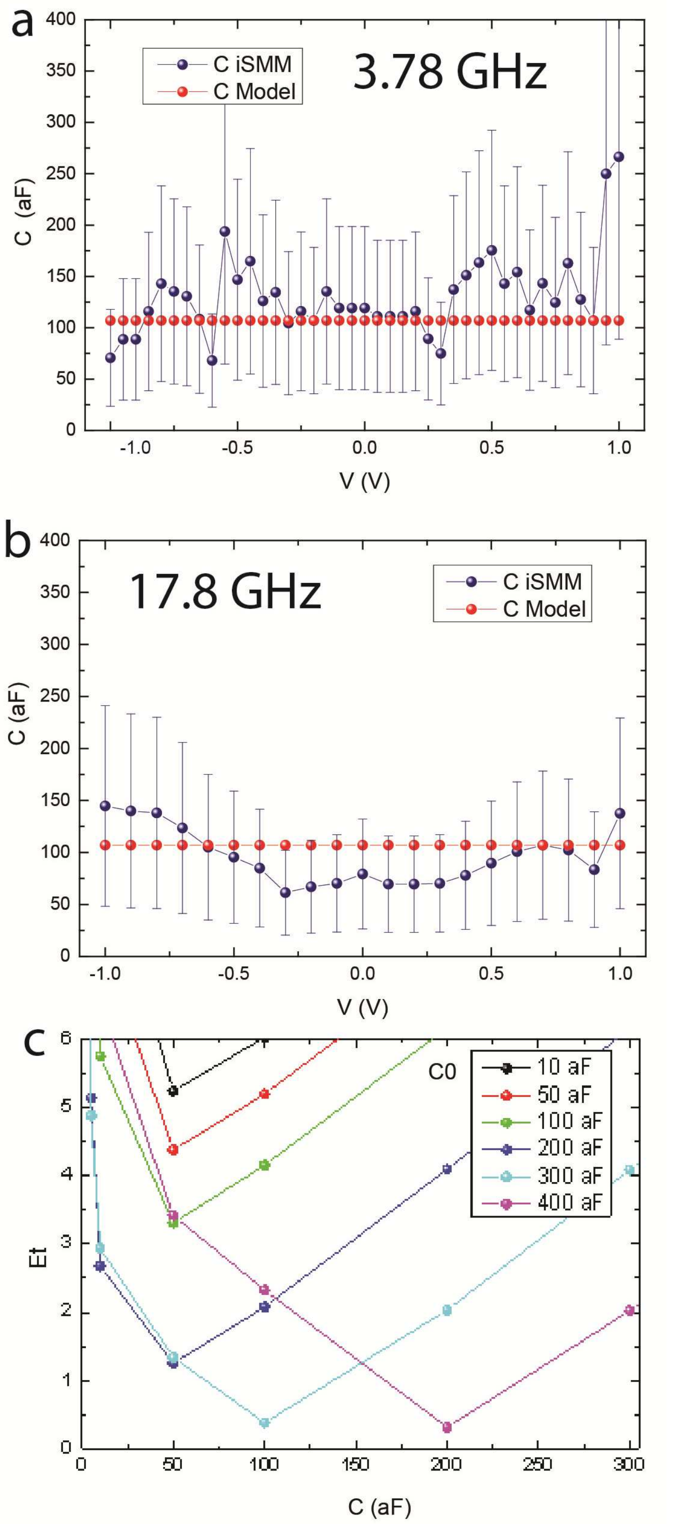

Supplementary Figure 9 a Estimated C_{ISMM} from the 2 parameters model (61dB -153°) at 3.78 GHz. **b** Estimated C_{ISMM} from the 2 parameters model (52.24dB -89.96°) at 17. 8 GHz. **c** Relative error in the fittings of conductance and capacitance using S_{11} with respect the conductance measured by resiscope and the capacitance used in the model. Several values of C_0 are displayed.

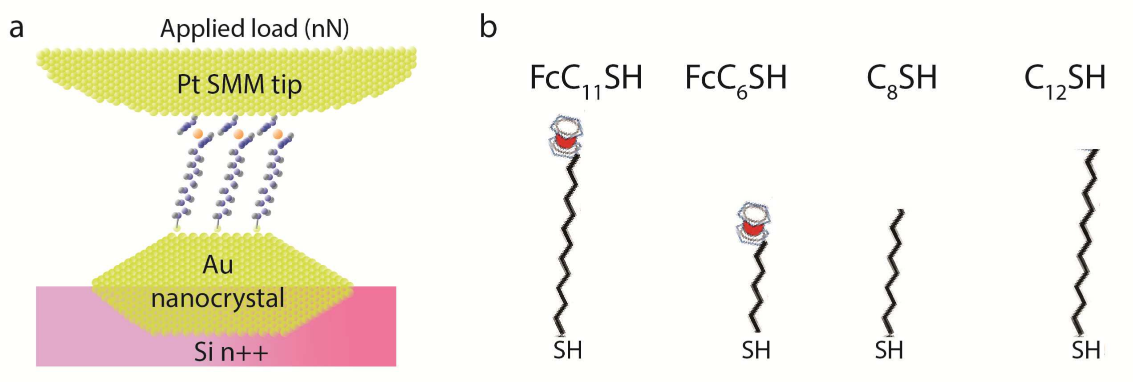

Supplementary Figure 10 a Schematic representation of the tip/molecules/Au nanocrystal structure showing that a load is applied on the tip. This parameter is tunable and its impact is discussed in that section. **b** Schematic representation of the four molecules tested in that study to evaluate the impact of molecule design on the performance of high frequency molecular electronics.

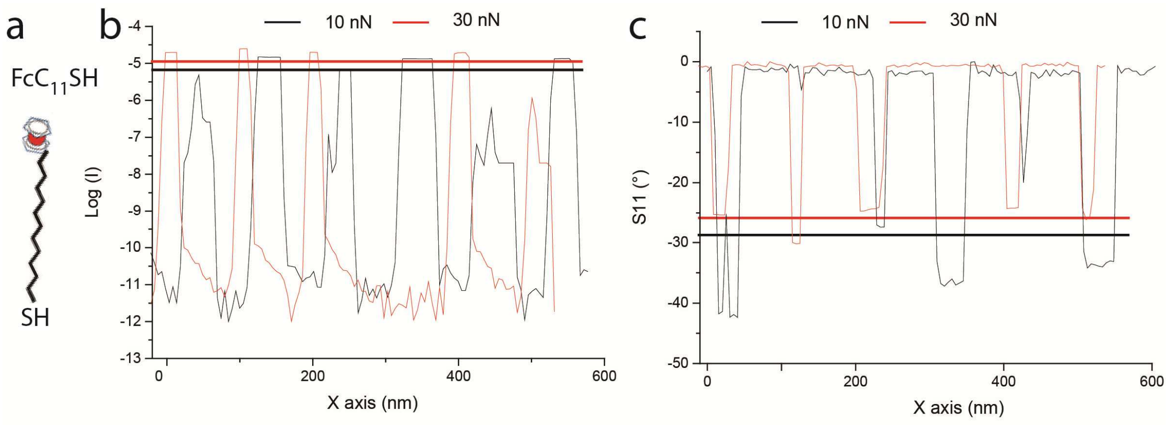

Supplementary Figure 11 a Schematic representation of the $FcC_{11}SH$ molecule used in this study. **b** Raw datas of the measured current (log scale in A) on 6 dots at 0.8V for 2 different forces of 10 nN et 30 nN. **c** Raw datas of the measured S_{11} Phase at 3.8 GHz, 0.8V on 6 dots for 2 different forces of 10 nN et 30 nN.

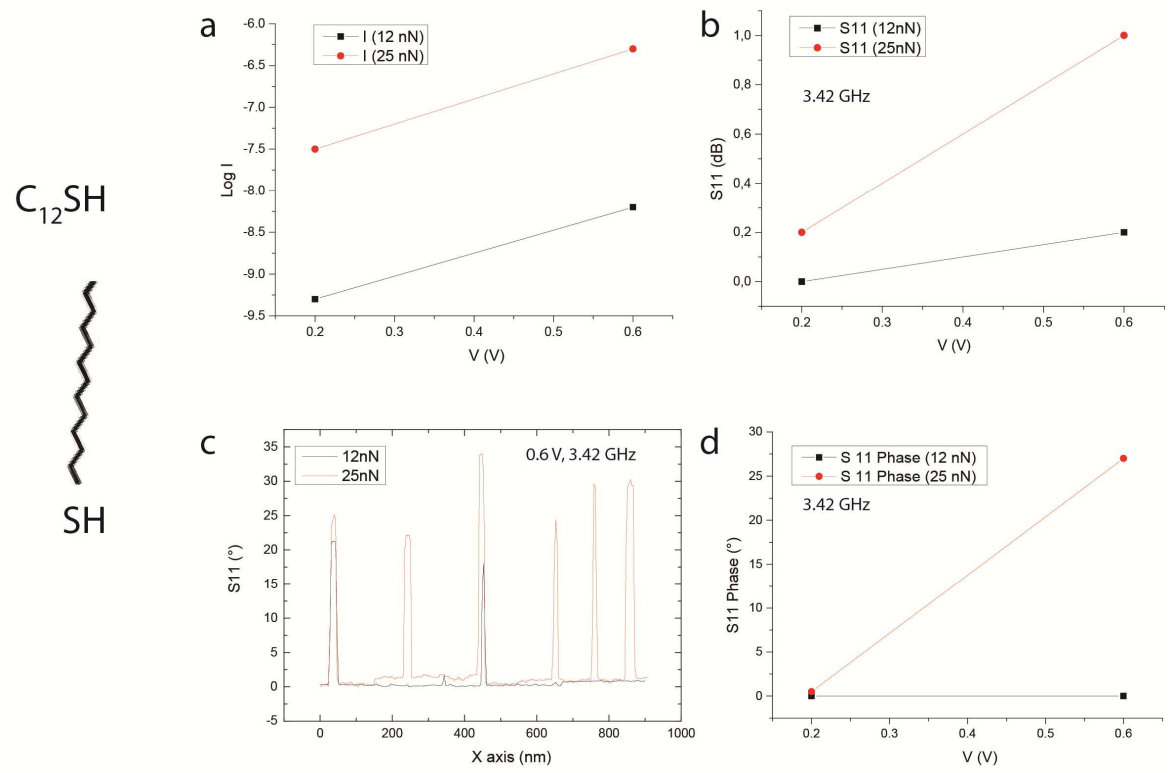

Supplementary Figure 12 Left: Schematic representation of a $C_{12}SH$ molecule. **a** Evolution of the measured current for $C_{12}SH$ molecules on gold nanocrystals at two different biases and two different tip loads. **b** Evolution of the measured $|S_{11}|$ for $C_{12}SH$ molecules on gold nanocrystals at two different biases and two different tip loads (frequency is 3.42 GHz). **c** Raw datas of S_{11} phase for 6 molecular junctions at two different loads (bias is 0.6 V and frequency is 3.42 GHz). The signal is very weak at 12 nN. **d** Evolution of the measured $|S_{11}|$ for $C_{12}SH$ molecules on gold nanocrystals at two different biases and two different tip loads (frequency is 3.42 GHz).

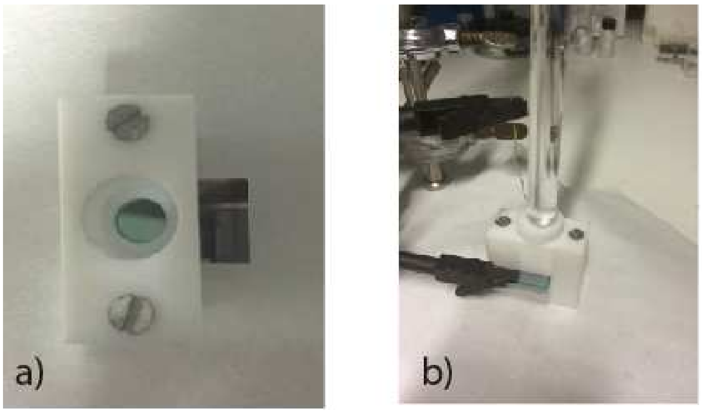

Supplementary Figure 13. a Electrochemical cell with a sample. **b** set up during the operation

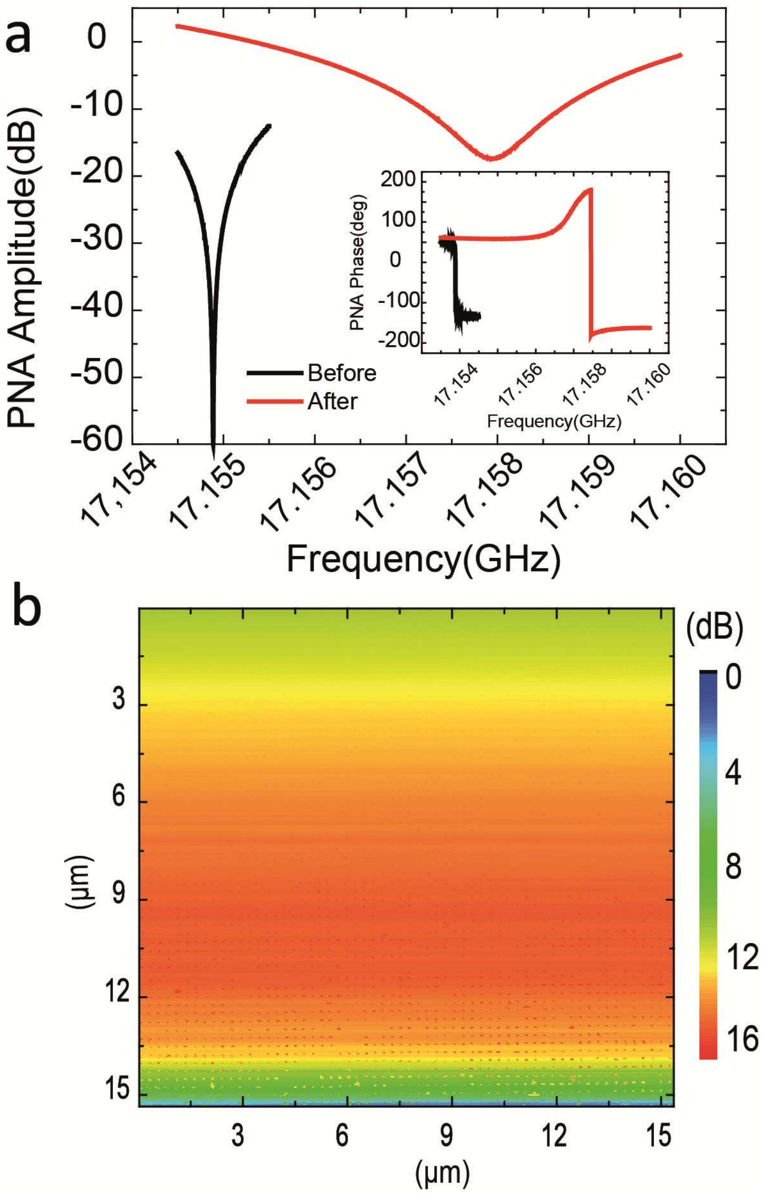

Supplementary Figure 14. a 17,157929 GHz spectrum before and after 1h 22 min. **b** S_{11} amplitude image over 3600 nanojunctions.

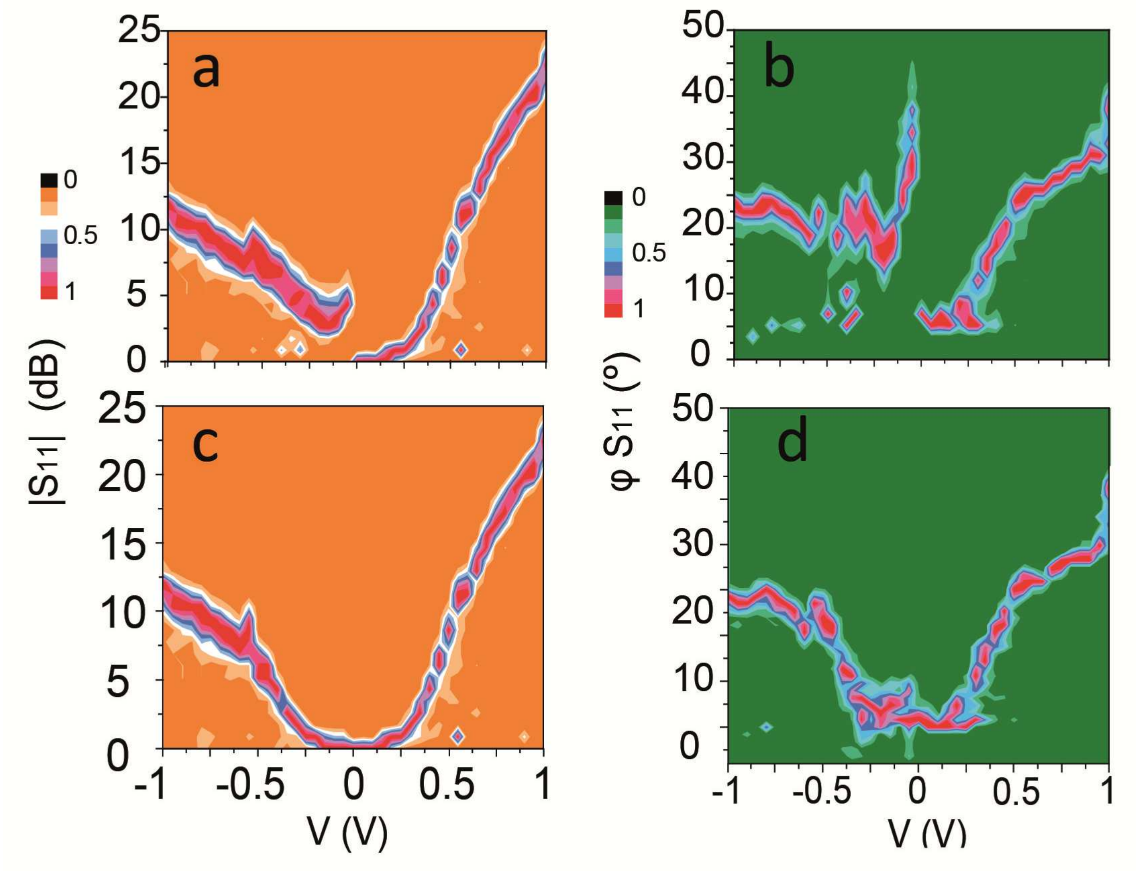

Supplementary Figure 15. a S_{11} amplitude row data at 3.78 GHz, **b** phase row data, **c** S_{11} amplitude corrected and **d** phase corrected.

Supplementary Table 1: Fitting parameters for cyclic voltammetry (100 mV/s)

**Model**	**Gauss**		
**Equation**	**y=y0 + (A/(w*sqrt(PI/2)))*exp(-2*((x-xc)/w)^2)**		
Reduced Chi-Sqr	3,73E-20		
Adj. R-Square	0,98846		
		Value	Standard Error
Peak1	y0	$2{,}33\text{x}10^{-11}$	$2{,}69\text{x}10^{-11}$
	xc	0,3365	0,00158
	w	0,08718	0,00239
	A	$4{,}44\text{x}10^{-10}$	$1{,}49\text{x}10^{-11}$
	sigma	0,04359	0,00119
	FWHM	0,10264	0,00281
	Height	$4{,}06\text{x}10\text{-}^{9}$	$1{,}20\text{x}10^{-10}$
Peak 2	y0	$2{,}33\text{x}10^{-11}$	$2{,}69\ \text{x}10^{-11}$
	xc	0,36883	$3{,}87\ \text{x}10^{-04}$
	w	0,02085	$9{,}40\ \text{x}10^{-04}$
	A	$1{,}52\text{x}10^{-10}$	$8{,}65\text{x}10^{-12}$
	sigma	0,01042	$4{,}70\text{x}10^{-04}$
	FWHM	0,02455	0,00111
	Height	$5{,}81\text{x}10^{-09}$	$2{,}08\text{x}10^{-10}$

Paper	Bottom Electrode /Wgnd (eV)	Top Electrode /Wbias (eV)	Junction area (cm^2)	Efc (eV)	α	J_{max} @(−1Vor 1V) (A/cm^2)	|J(+1)/ J(−1)|	Rectification direction (sign of Vbias)	ΔV (eV)
Ref.16	Ag/4.5	EGaIn/4.3	$3.14x10^{-6}$	5.05	0.8	$5.00x10^{-4}$	151	−	−0.71
Ref.17	Ag/4.3	EGaIn/4.3	$3.14x10^{-6}$	5.05/4.8*	0.8	$1x10^{-3}/5x10^{-3}$	610/1100	−	−0.75/−0.5
Ref.18	Au/4.2	PEDOT-PSS/5.05	$2x10^{-6}$	5.05	0.8	$4x10^{-7}$	1.5	−	−0.17
	Au/5.1**							=	0.01
Ref.19	Au/4.2	Au NP/4.2	?	5.05	0.5	?	2***	−	−0.85
	Au/4.8	/4.8							−0.25
Ref.20	Au/4.2	STM tip/?	$3x10^{-15}$****	5.05	0.8	$1.6x10^{+3}$	2	+	
					0.65				
Ref.21	Au/4.2	STM tip/5.1	$3x10^{-15}$****			$1x10^{+5}$	1.5	−	−0.13
This Work	Au/4.2	Pt/5.5	$3.14x10^{-12}$	5.05	0.8	$2x10^{+7}$	5	+	0.19
	Au/4.8							+	0.31

* Two types of configurations for Fc
** Value considered in their paper
*** We considered their Fig.4c, e.g. a configuration as close as possible from other works
**** We considered only one molecule measured with the STM tip

Supplementary Table 2 Comparison of properties of various molecular diodes using a Fc thiol monolayer

Supplementary Note 1 Cyclic voltammogram analysis.

Supplementary Figure 5a shows the raw data for the cyclic voltammogram measured at a scan rate of 0.1 V/s on an array of 5.1×10^7 nanodots (20 nm diameter) functionalized with $FcC_{11}SH$ molecules, and Supplementary Figure 5b shows the same data after correction from the capacitive contribution (the base line is removed using the baseline function from Origin software). The capacitive contribution is mainly due to the presence of a thin layer of silicon dioxide between dots. Lee et al.[8] reported an anodic peak deconvolution method using Gaussian and Lorentzian functions to probe the heterogeneity of the SAMs. In our CVs, we didn't notice a clear difference with the use of a Lorentzian function due to the sharp second peak, so peak deconvolution was performed with two Gaussian functions. Results from fits obtained with two Gaussian curves are shown in Supplementary Table 1. Supplementary Figures 5c-j show cyclic voltammograms (forward and backward) at four different sweep rates (100 mV, 300 mV, 1V and 2V). These 8 figures are used to extract an averaged density of molecules per dot of 0.405 +/- 0.04 nm^2/molecule (Supplementary Figure 4). The error bar is mainly related to the uncertainty on the area per dot.

For SAMs of SC_nFc on Au, the theoretical value of 0.37nm^2/molecule (or a density of 4.5×10^{-10} mol/cm^2) is usually considered assuming a hexagonal packing with Fc treated as spheres with a diameter of 6.7Å[9]. This value has been measured for the fully packed monolayers[10]. In this study, the averaged density of molecules on the gold nanocrystals is ~10% lower than for a fully packed monolayer, but in the range of values found for a dense monolayer. According to ref.10, the 10% lower density is observed at reduced van der Waals interactions, for example for a monolayer of SC_8Fc. In ref.10, they observed a new redox wave at low oxidation potentials for highly

disordered SAMs when disulfides were present in the thiol precursors and a very broad redox waves when rough electrodes were used. Although we cannot exclude the presence of disulfides in the thiol precursor (we have not performed an additional purification step), their density may be small as such additional peak is not clearly observed in our cyclic voltammograms. Also, we did not observed broad voltammograms which may be explained by the small dispersion on gold nanocrystals structure.

From the CV curves, we can estimate the surface coverage Θ, i.e. the number of molecules per dot and the area occupied per molecule using the following equation :

$$\Theta = Q_{tot}/nFA \qquad \text{Supplementary Equation 1}$$

where Q_{tot} is the total charge involved during the redox process, n is the number of electrons transferred during the reaction (here 1), F the Faraday's constant (96 485 C/mol) and A the surface area. Q_{tot} is given by the integral under the CV curve taken into account the scan sweep, v=0.1 V/s, we get Q_{tot}=6x10^{-9}C from Fig. S5-b. The total area is the area of the nanodot multiplied by the number of dots. Considering a truncated cone of 20 nm diameter basis, 30 degree angle and 3 nm thick (Supplementary Figure 4c) gives an area of 72 nm^2 on the top part and 278 nm^2 on the sides, we obtain a surface of 350 nm^2 per dot. With 5.1x10^7 nanodots on a 500 µm x500µm (nanodot pitch 70 nm), A=1.785x10^{-4} cm^2.The estimated area per molecule of ~0.405 nm^2, corresponds to a number of FcC_{11} molecules between the iSMM tip and the electrode of ~150 molecules.

Supplementary Note 2 Estimation of E_{HOMO} and Energy band diagram

The energy of the HOMO, E_{HOMO}, relative to the vacuum is estimated from the voltammogram wave using:

$$E_{HOMO}=-e(E_{1/2}+E_{Ag/AgCl-NHE})+E_{abs,NHE} \quad \text{Supplementary Equation 2}$$

with e the electron charge, $E_{abs,NHE}$ the absolute potential energy of the normal hydrogen electrode (NHE) at -4.5 eV[11], $E_{(Ag/AgCl-NHE)}$ is the potential of the Ag/AgCl versus the NHE (0.197 V). $E_{1/2}$ is the half-wave potential measured on the voltammograms. For a redox species immobilized on a surface, the anodic and cathodic potential are ~ equal (Supplementary Figures 5c,d), and $E_{1/2}$ is given by the peak position. Cyclic voltammograms are fitted considering two peaks at 0.37 V and 0.34V vs Ag/AgCl (Supplementary Table 1), so we consider only the level with larger E_{HOMO}. We obtain $E_{HOMO}\simeq$-5.05 eV as the related energy level in the molecular junction. Supplementary Figure 6 shows the proposed energy diagram of the molecular junction. We used theoretical values for the work functions of the Pt electrodes (≈5.5 eV[12,13]) and Au electrode (4.2 eV) following Ultraviolet Photoelectron Spectroscopy (UPS) results from ref.10 (Au with FcC_{11} SAM). This schematic diagram is intended to explain the rectification observed for a positive bias applied on the Pt tip.

Supplementary Note 3 Direction/strength of Rectification and current densities[14-19]

In Supplementary Table 2, we compare the difference of energy ΔV between the Ferrocene HOMO level and the energy level of the biased top electrode in a molecular junction configuration at 0V (Supplementary Figure 6) for seven different studies that used a FerrocenylAlkylthiol molecule, including the present work. The sign of ΔV provides a relevant indication on the direction of rectification (e.g. $\Delta V<0$ favors a diode "on" at negative bias, the usual case) and could be expressed analytically as:

$$\Delta V=(W_{bias}-E_{Fc})-(1-\alpha)(W_{bias}-W_{gnd}) \qquad \text{Supplementary Equation 3}$$

, where W_{bias} and W_{gnd} are the metal work functions of the biased and grounded electrodes respectively, α is the parameter of coupling between the Ferrocene groups and the biased electrode (in the Landauer formula[7], it is defined as $V_b^2/(V_g^2+V_b^2)$ where V_b and V_g represent the energies of coupling to the biased and grounded electrodes, respectively), and E_{Fc} is the energy position of the Ferrocene HOMO level related to vacuum. For simple comparison and discussion, we first considered the same coupling parameter α=0.8 for all studies (Supplementary Figure 6). Interestingly, the Supplementary Equation 3 leads to $\Delta V>0$ only in the case of the Pt biased electrode with large work function (Supplementary Table 2) used in this study. In the case of a top electrode with a relatively large work function such as PEDOT-PSS, $\Delta V\sim 0$ (a very small rectification at negative bias was observed). In other words, the difference in rectification direction in the present work compared to others could be mainly explained by the large work function of the Pt top electrode using an energy band diagram with a single energy level. The supplementary Equation 3 stresses the drastic importance of W_{bias} if α gets close to 1. Nontheless, as clearly demonstrated in ref.20, the monolayer

organization plays a key role in the “off” state, with large leakage (lower rectification) when the monolayers are not perfectly packed. We also expect that this plays a key role in addition to the metal work function.

Note that for RF molecular rectifier applications such as mixers, the figure of merit is the conductance in the “on” state as the non linearity close to this biasing point is exploited.

The current density has been extensively discussed in the main text. We can note that the current density for a single molecule junction (such as a STM tip top electrode) is still 2 orders of magnitude lower than that in the present study, which can be explained by the weak coupling between the STM tip and the Ferrocene molecules.

Supplementary Note 4. Effect of the Tip load and the Molecule

We have also investigated the effect of the tip load and the molecule types (Supplementary Figures 10-12). In the case of the nanocrystal structure, there is no increase of junction area with the tip load unlike for experiments performed on full substrate[21], but tip load are typically in the same range. For $FcC_{11}SH$ molecules, we have noticed a weak effect of the tip load on the measured current and S_{11} parameters (Supplementary Figure 11), unlike for $C_{12}SH$ molecules (Supplementary Figure 12) where almost 2 orders of magnitude difference in current was observed when the load was changed from 12 nN to 25 nN. As mentioned on the main text, the weak effect of the load for $FcC_{11}SH$ molecules could be a signature of weaker van der Waals interactions (reduced monolayer thickness). We have previously shown that short molecules such as C_8SH depend weakly on tip load, which is simply explained by the Hooke formula (small thickness change)[22], and deformation of electrodes.

In the case of strong van der Waals interactions or for thick monolayers, the effect of tip load provides another degree of freedom to tune f_t. We illustrate this for $C_{12}SH$ molecules (Supplementary Figure 12). At low tip load (12 nN), we observe almost no effect of the tip bias on the S_{11} phase as expected for a pure capacitor: f_t is below 3.4 GHz on the whole voltage bias. Increasing the load to 25 nN enables to observe a difference in the phase and amplitude as expected for a tunnel junction with f_t>3.42 GHz at least for $|V|$>0.5 V. Considering a tip load of 30 nN to keep f_t high enough for both $C_{12}SH$ and C_8SH molecules, we confirmed that no clear rectification was observed neither in the current nor on $|S_{11}|$ (Supplementary Figure 7). Note that the current levels are in the same order of magnitude for the C_8SH and the $FcC_{11}SH$ molecule in the "on" state, but the slope is larger for $FcC_{11}SH$ molecule (Supplementary

Figure 8). Therefore, $FcC_{11}SH$ molecule is more appropriate for high performance RF diodes (e.g. mixer application: use of the nonlinearity at the biasing point in the "on" state).

We have also tried to study FcC_6SH, but we systematically reached unsustainable current densities for the gold nanocrystals.

Supplementary Methods:

S_{11} model and calibration

Under the hypothesis of a perfect interferometer, no parasitic reflection or mismatch among the components is taken into account. In the case of Z_{DUT} (impedance of the device under test) much higher than Z_c (characteristic impedance), the ratio S_{11} between the wave transmitted after the interferometer to the receiver and the wave coming from the RF source can be approximately considered as proportional to the admittance of the device according to[23]:

$$S_{11} \approx A\Gamma + B \qquad \text{Supplementary Equation 4}$$

where A and B are two complex parameters. $\Gamma = \frac{Z-Z_C}{Z+Z_C}$ is the DUT reflection coefficient and Z is the DUT impedance. For small DUT admittances $Y = \frac{1}{Z} \ll \frac{1}{Z_C}$, Γ can be rewritten as $\Gamma \approx 1 - 2Z_C Y$ and the Supplementary Equation 4 becomes:

$$S_{11} \approx -2Z_C A(\,Y - Y_0) \qquad \text{Supplementary Equation 5}$$

where $Y_0 = \frac{A+B}{2Z_C A}$. The complex factor "*A*" takes into account losses, gain and shifts due to cables, passive and active elements. Y_0 is the admittance for which fully destructive interference occurs (S_{11}=0).

In order to fit the measured S_{11}, we considered an equivalent electrical circuit composed of a resistance and capacitances (intrinsic and extrinsic) in parallel.

The measured admittance Y will therefore be modeled by equation:

$$Y = G + C\omega j \qquad \text{Supplementary Equation 6}$$

where ω is the angular frequency ($2\pi f$).

G is the (dynamic) conductance that can be derived in a first approach from the derivative of the (DC) *I-V* curve due to the absence of photocurrent, C is given by $C_{mol}+C_p$.

In order to use the Supplementary Equation 5, we need to identify *A* and Y_0. To do that, we need to perform two measurements of S_{11} and use the corresponding values of *G* and *C* as a model. First, the interference is set for $S_{11}=0$ and is measured when the tip is on the SiO_2 surface (between the dots). Then the two points are chosen for given DC voltages among the measurements on the molecules to obtain the best fit. *G* will be obtained from the DC conductance and we assume that *C* does not change with the voltage. *C* is estimated as the value that gives the best fitting when comparing the conductance measured at DC and the conductance extracted from S_{11}. The error E_t is calculated as the sum of the fitting error in capacitance and conductance obtained by equations (Supplementary Figure 9) where C_M and G_R are respectively the modeled capacitance and the conductance measured by the resiscope and C_{iSMM} and G_{iSMM} are the capacitance and conductance extracted from S_{11}:

$$E_C = \frac{C_M - C_{iSMM}}{C_M} \qquad \text{Supplementary Equation 7}$$

$$E_G = \frac{G_R - G_{iSMM}}{G_M} \qquad \text{Supplementary Equation 8}$$

$$E_t = E_C + E_G \qquad \text{Supplementary Equation 9}$$

We see that when we go from, for example, C_0= 350 aF to C_0= 470 aF the minimum in the relative error of the conductance move from 42% to 20%. We can see how all our

variables have a relationship. C_0=300aF is close to the calibrated value measured on the same wafers and same SMM tips and it is a reasonable value looking at the literature[24]. In Supplementary Figure 9 we can see that the corresponding values of nano molecular junctions that minimizes the error is around 107 aF. This value is still far from the supposed value of few aF corresponding to the molecules alone.

Supplementary References:

[1] Clément, N.; Patriarche, G.; Smaali, K.; Vaurette, F.; Nishiguchi, K.; Troadec, D.; Fujiwara, A.; and Vuillaume, D.; Large Array of Sub-10-nm Single-Grain Au Nanodots for use in Nanotechnology, *Small* **7**, 2607-2613 (2011)

[2] Dargent, T,; Haddadi, K.; Lasri, T.; Clement, N.; Ducatteau, D.; Legrand, B.; Tanbakuchi, H.; and Theron, D.; An interferometric scanning microwave microscope and calibration method for sub-fF microwave measurements, *Rev.Sci.Instrum.* **84**, 123705-123712 (2013)

[3] Resiscope user guide: http://www.scientec.fr/PDF/surface/agilent/ResiscopeII-ScienTec.pdf

[4] Umana, M.; Rolinson, D.R.; Nowak, R.; Daum, P.; Murray, R.W.; X-Ray Photoelectron spectroscopy of metal, metal oxide, and carbon electrode surfaces chemically modified with ferrocene and ferricenium, *Surf.Sci.* **101**, 295-309 (1980)

[5] Shirley, D.A.; High-Resolution X-Ray Photoemission Spectrum of the Valence Bands of Gold *Phys. Rev. B* **5**, 4709-4714 (1972)

[6] Nijhuis, C.A. et al.; Molecular Rectification in Metal−SAM−Metal Oxide−Metal Junctions *J.Am.Chem.Soc.* **131**, 17814-17821 (2009)

[7] Trasobares, J. et al. *Estimation of π-π* intermolecules interaction energy from conductance histograms*.; In preparation.* In that paper, the parameter α is obtained from the ratio of the coupling parameters to the Fc molecule. The configuration is similar in the present paper, with a Pt electrode instead of a PtIr tip.

[8] Lee, L.Y.S. et al. Ferrocenylalkylthiolates as a Probe of Heterogeneity in Binary Self-Assembled Monolayers on Gold, *Langmuir* **22**, 4438-4444 (2006)

[9] Chidsey, C.E.D; Bertozzi, C.R.; Putvinski, T.M.; Mujsce, A.M.; Coadsorption of ferrocene-terminated and unsubstituted alkanethiols on gold: electroactive self-assembled monolayers, *J.Am.Chem.Soc.* **112**, 4301-4306 (1990)

[10] Nerngchamnong, N.; et al.; Nonideal Electrochemical behavior of Ferrocenyl-Alkanethiolate SAMs maps the microenvironment of the Redox unit *J.Phys.Chem.C.* **119**, 21978-21985 (2015)

[11] Bard, A.J., Faulker, L.R.; Electrochemical methods: Fundamentals and applications, Wiley, New-York, 2001

[12] Michaelson, H.B. et al.; The work function of the elements and its periodicity, *J.Appl.Phys.* **48**, 4729-4733 (1977)

[13] Wang, X.F. et al.; The canonical work function-strain relationship of the platinum metal. A first-principles approach to metal-gate transistor optimization, *Appl.Phys.Lett.* **102**, 223504-223507 (2013)

[14] Nerngchammong, N. et al.; The Role of van der Waals forces in the performance of Molecular Diodes *Nat. Nanotech.* **8**, 113-118 (2013)

[15] Yuan, L.. et al.; A Molecular Diode with a Statistically Robust rectification Ratio of Three orders of magnitude *Nano Lett.* **5**, 5506-5511 (2015)

[16] Jeong, H. et al. Redox-Induced Asymmetric Electrical characteristics of Ferrocene-Alkanethiolate molecular devices on rigid and flexible substrates *Adv.Func.Matter.* **24**, 2472-2480 (2014)

[17] Mentovich, E.D. et al. *J.Phys.Chem.C.* **117**, 8468-8474 (2013)

[18] Muller-Meskamp, L. et al. Field-Emission Resonances at Ti/α,ω- Mercaptoalkyl Ferrocene/Au Interfaces studied by STM *Small* **5**, 495-502 (2009)

[19] He, J. et al., On the Mechanism of Negative Differential Resistance in Ferrocenylundecanethiol Self-Assembled Monolayers, *J.Am.Chem.Soc.* **127**, 11932-11938 (2005)

[20] Jiang, L. et al. Controlling Leakage currents: The role of the binding group and purity of the precursors for self-assembled monolayers in the performance of molecular diodes, *J.Am.Chem.Soc.* **136**, 1982-1990 (2014)

[21] Engelkes, V.B.; Beebe, J.M.; and Frisbie, C.D.; Analysis of the causes of variance in resistance measurement on Metal-Molecule-Metal junctions formed by conducting-probe Atomic Force Microscopy, *J.Phys.Chem.B* **109**, 16801-16810 (2005)

[22] Smaali, K.; Desbief,S.; Foti, G.; Frederiksen,T.; Sanchez-Portal, D.; Arnau,A.; Nys,J.P.; Leclère,J.P.; Vuillaume,D.; and Clément, N.; On the mechanical and electronic properties of thiolated gold nanocrystals, *Nanoscale* **7**, 1809-1819 (2015)

[23] Michalas, L. et al. Modeling and de-embedding the interferometric scanning microwave microscopy by means of dopant profile calibration, *Appl. Phys. Lett.*, **107**, 223102-223105 (2015).
[24] Gomila, G. et al., Finite-size effects and analytical modeling of electrostatic force microscopy applied to dielectric films., *Nanotechnology*, **25**, 255702-255710 (2014)
