# Supplementary material for: A 17 GHz molecular rectifier
Source: Nat Commun. 2016 Oct 3;7:12850. doi: 10.1038/ncomms12850 (PMC5059435; doi:10.1038/ncomms12850)
Supplement: Peer Review File [file ncomms12850-s2.pdf]

Reviewers' comments:

Reviewer #1 (Remarks to the Author):

The manuscript describes a previously unknown mechanism of Hsp70 regulation by post-translational lysine acetylation. It is proposed that stress induced Hsp70 is acetylated on Lys77 upon stress, which increases its chaperone activity and binding to Hsp90 and cofactor Hop. Later on the acetylation is lost and more binding to the cofactors Hsp40 and the E3 ubiquitin ligase CHIP is observed. Hsp70 acetylation is by ARD1, and deacetylation by HDAC4. The Lys77 site is Arg77 in Hsc70 which therefore does not have such regulation. The non-modifiable Hsp70 mutants K77R and acetylation mimic K77Q recapitulate the cofactor interactions. K77R but also K77Q is less protective than WT Hsp70, and it is proposed that an acetylation regulated switch from folding to degradation is important for Hsp70 in the response to stress.

The mechanism, if correct, is a novel finding and will be of broad general interest. As the authors point out, it would provide a fast response to stress independent of the transcriptional response. While a number of post-translational modifications of Hsp70 have been reported, many of their functional effects have been uncertain.

There are, however, a number of serious technical concerns that have to be addressed before publication can be recommended. The most important (point 1) is the use of cells and zebrafish which are mostly dead or dying. There are also questions about some of the in vitro and functional experiments. Because a link between the acetylation site and cofactor binding site is unknown, a high standard of data is required.

1. The stress conditions used, typically 1 mM peroxide for up to 24 h, is harsh enough that there are doubts about physiologic relevance of the late phase loss of acetylation. The model (Fig. 7) indicates time after stress suggesting recovery, but in reality the experiments address the time/harshness of stress with no recovery phase. In Fig. 6, between 40% and 70% of SH-SY5Y cells are dead after treatment. It is harder to make firm conclusions about the biology of cells when disruption is this extreme. Indeed, the harshness of the treatment was reduced to 0.3 mM peroxide to detect apoptosis markers. At higher peroxide levels it is possible that cell death is more due to broad mechanical disruption by irreversible oxidative damage.

If the acetylation-deacetylation mechanism contributes to cellular function over a range of stress conditions, it should also be observable in cells which are recovering from shorter stresses. There is evidence in supplemental Fig. 1c, where cells are treated with 1 mM peroxide for 1 h then allowed to recover for 24 h, and the acetylation change is detected. Key experiments should be re-done using similar stress and recovery experiments.

2. The stress conditions used were all related to oxidation. Is the acetylation also observed under other conditions that cause the transcriptional heat shock response such as heat or Hsp90 inhibition? Related to this, a heat shock response is not observed in the HEK293T cells with peroxide, and another stress that causes the response could be used to show acetylation of endogenous Hsp70.

3. In Fig. 2c, the acetylation state of Hsp70 in the HEK293T cells with knockdown of must also be shown. It is only shown for SH-SY5Y in Fig. 2b.

4. The lysine-directed activity of ARD1 has been in question because of its identification as N-terminal acetyltransferase, most recently in Magin et al. J Biol Chem. 2016 291(10):5270-7. The paper reported non-enzymatic chemical acetylation of lysines by acetyl-CoA in the absence of ARD1, that was less efficient than ARD1-dependent N-terminal acetylation. The experiments in Fig. 3a indicate that true lysine acetylation is observed. To make this even stronger, the negative control reaction lacking ARD1 should be repeated for full-length Hsp70 in the time course, Fig. 3c.

5. In Fig. 4c, the unstimulated ATPase activity of Hsp70 is reported in relative terms. However, in cells Hsp70 is constantly interacting with Hsp40/J-domain proteins and nucleotide exchange factors which stimulate its ATPase rate. To better represent the physiological state, rates in the presence of these cofactors (eg. Hsp40 and Hsp105) should be tested.

6. The protein concentrations in the in vitro experiments are not given (ATPase assay, acetylation, Hop and CHIP binding, ATP-agarose binding). This is very important for the experiment showing competition between Hop and CHIP, Fig. 5b, to evaluate the relative strength of binding. Because the antibodies are different, the blots do not report on the absolute amounts of each protein in the inputs.

7. The luciferase refolding in Fig. 5e is in question. The procedure is very different from the reference cited, Ran et al. Cell Stress Chaperones. 9, 229-242 (2004). In that paper, luciferase expressed in cells was denatured by a 45{degree sign}C heat stress followed by 3 h of refolding at 37{degree sign}C. This is similar to methods used by other groups (eg. Nollen et al. PNAS 2001 98(21):12038-4). Peroxide is not used for these experiments. One reason is that heat denaturation is reversible except for aggregation, but protein damage by oxidation is mostly irreversible. It seems surprising that there is any significant regain of activity, from this viewpoint. The authors should use the established heat stress methods, or include more data to establish the peroxide method. For example, how much luciferase activity is refolded compared to before denaturation, what the are levels of soluble luciferase before and during refolding, is refolding inhibited by Hsp70 knockdown or inhibition, what are refolding levels without Hsp70 overexpression.

8. Fig. 5f claims to represent protein degradation but it actually shows total protein ubiquitination after proteasome inhibition. This is related but is not a direct measure of degradation. Images of the blots should be shown. For Fig. 5g, aggregation at the early phase should also be shown. Fig. 5e and f are lacking controls without overexpressed Hsp70.

9. The identification of HDAC4 as the relevant deacetylase in Supplemental Fig. 5 should be supported by knockdown.

10. It is suggested that as the acetylation is lost, CHIP binding increases. But in the late phase, much more CHIP is bound than before stress, when Hsp70 is also not acetylated (Fig. 1). The lack of acetylation alone does not account for the increase in CHIP binding. Part of the result may be due to abnormal behavior of proteins cells which are dying. In any case, the authors should suggest some explanation. In cells, phosphorylation of T636 changes binding from CHIP to Hop (Muller et al. Oncogene (2012), 1-10), and this should be tested to rule out such a mechanism.

11. I recognize there is no easy way to address how the acetylation site can affect the C-terminal binding site for CHIP and HOP. Still, for a journal of this caliber, a better discussion of the possibilities is expected. On page 17 acetylation "could alter co-factor binding to the SBD", they bind to the flexible extension of the CTD, not SBD, using the terminology of the manuscript. The interactions of the NBD to SBD and CTD require ATP, and the in vitro binding of Hop and CHIP in Fig. 5b are in the absence of ATP, when the NBD does not contact the SBD/CTD.

12. Similarly to point 1., in supplement Fig 7, 30% to 60% of zebrafish are dead after the 1 h treatment with 10 mM peroxide, which seems very harsh. Experiments with milder conditions where closer to 100% of the animals survive in the control would be better.

Reviewer #2 (Remarks to the Author):

This manuscript reports significant results that describe the dynamics of Hsp70 chaperone-mediated stress response. Moreover, the results present an elegant mechanism that differentiates the behavior of Hsp70 and the very closely related Hsc70 in stress response, sheds light on stress response in transformed/cancer cells in which Hsp70 is present at highly elevated levels in the absence of added external stressors, and provides a mechanism for rapid modulation of stress response that is not based on HSF1-mediated chaperone transcription. In brief, the authors demonstrates that acetylation by ARD1 on a specific residue of Hsp70 (K77) regulates the association with Hop, a pro-folding cochaperone, vs. CHIP, a degradative cochaperone. The

kinetics of acetylation and deacetylation following stress allow the chaperone to shape the cell's stress response, resulting in a rapid pro-folding biased response, followed by slower return to pro-degradation.

The manuscript is very clearly written (and indeed, was actually a pleasure to read). Premises/hypotheses are clearly and thoroughly tested and the range of investigation is quite extensive, including experiments in a zebrafish model of stress response. The authors' conclusions about the mechanisms and potential regulatory importance of Hsp70 acetylation by ARD1 are well justified. This is a novel, important and well-conducted study that will be of substantial interest to investigators in the chaperone / protein-quality control field that reveals a compelling new mechanism for cochaperone-switching and for the functional changes that accompany cochaperone-switching.

I have no major objections or critical comments. It would be appropriate for the authors to address one point:

1. It was previously reported that phosphorylation of the Hsp70/Hsc70 tail, including (possibly) by stress-responsive kinases, could also modulate association with Hop vs. CHIP (Muller et al., Oncogene 32(25), 2013; Assimon et al., Biochemistry 54(48), 2015) and that phosphorylation of Hop could inhibit its association with Hsp70 (Röhl et al., EMBO Rep. 16(2), 2015). Could the authors comment on these results and integrate them into their overall model?

A few very minor points:

1. Page 3, "the extensive NBD surface interacts with the SBD" should probably be "an extensive NBD surface interacts with the SBD"
2. Page 6 "Because the chaperone function of Hsp70 is decided by its co-chaperone" - this seems an odd way to phrase what the authors are trying to convey.
3. Page 11, lower section - "we next determined whether the constituency of the the Hsp70 co-chaperone complex" - the authors may want to use "composition" rather than "constituency" here.
4. Page 18, near top - "therefore, denatured proteins can be excessively regulated by the protein degradation pathway" - it is not clear what the authors mean by "excessively" here - perhaps they mean "more extensively" or "more strongly"?
5. Fig. 1A, right panel - should the lowest strips in the IP and Input panels be labeled "Hsc70" rather than "Hsp70"?
6. Fig. 1A/B - histograms - the values plotted are normalized relative to an arbitrary value of 1 at time = 0 hours for both Hsp70 and Hsc70. It would, however, be interesting to compare the intensities (propensity to bind) relative to each other between Hsp70 and Hsc70. Do Hsc70 and Hsp70 actually bind Hop (or CHIP) similarly at time zero, or do they intrinsically have

somewhat differing affinities for these cochaperones? From IPs in panels A, it appears superficially that Hsc70 binds more strongly to CHIP and more weakly to Hop even at time zero but it would be interesting to quantitate this.
7. Fig. 1A/B histograms - Also, I am curious as to why the zero time points don't show error bars in this plot and panel C - did everything get normalized to a single time zero value, or were the time zero values of the individual trials each considered to be "1" for that trial?
8. Fig. 1C, right panel - are the measures of significance relative to the immediately preceding data point? It's not clear.
9. Fig. 4D; The nature of the ATP binding experiment is unclear from the figure and the legend only, though it is explained in the methods section. It would be helpful if the authors could briefly mention in the figure legend to the fact that Hsp70/Hsc70- are being pulled down with ATP-agarose beads in this experiment.
10. Fig. 5E-G. Similarly, the nature of the protein refolding, degradation and aggregation assays in panels E-G are unclear unless the reader looks at the methods section. A brief mention of the nature of each assay in the figure legend would be very helpful.

** Statistics in the manuscript are mostly appropriately used, in my estimation. The number of trials in most of the western blot assays are low (typically n=3) but no more so than typical for these types of assays; they are appropriately evaluated using two-tailed Student's t-tests. I raise a slight concern about the zebrafish locomotor activity measurements shown in Figure S7, panels E/F where a normal distribution may not apply due to the zero value cutoff, so a different significance test may be more appropriate.

Reviewer #3 (Remarks to the Author):

Article Summary:
In this study the authors identify and characterize an acetylation switch on Hsp70 that dictates client refolding (mediated by Hop and Hsp90) versus ubiquitin-dependent degradation (mediated by Hsp40 and CHIP). This acetylation switch involves ARD1-dependent acetylation of Hsp70 on K77, which is evolutionarily conserved. The acetylation-dependent switch has important implications for ATPase activity, client degradation and cell survival. The authors demonstrate their results in both human cell lines and the zebrafish model system.

This is a solid body of work. The experiments are well controlled and thorough.

I have only minor comments:

1) The CHIP signal in some blots appears different than others - sometimes there is an upper

band and sometimes not and its unclear why this is. For example, compare Fig 1 A left (single band) and right (doublet, unclear which is CHIP), and 5A (doublet, presumably only upper band is CHIP). The authors should explain this discrepancy and should always indicate background bands with an asterisk.

2) The authors should indicate the percentage input and IP loaded for ARD1 and HDAC4-Hsp70 interactions. This is important because it will give an idea of the fraction of Hsp70 that is in complex with these enzymes. This could have important implications for the model of how Hsp70 acetylation is regulated. For example, is most Hsp70 complexed with ARD1? If so, is the interaction modulated over time or by stress? My intention is not necessarily to have the authors test this, but it is an important point for discussion, which is facilitated by knowing the percent of each protein population in the complex.

3) Figure 4D and the meaning of Hsp70-ATP needs to be better explained. In general, the figure legends could be a bit more descriptive.

Reviewer #4 (Remarks to the Author):

The mass spectrometric identification of the claimed acetylation site looks very solid. I carefully looked at the tandem mass spectrum of the K77 acetylated HSP70 peptide in Figure 3D and manually annotated the spectra to validate the identification. All the major backbone (b- and y-type) fragment ions are present and as expected from the peptide sequence the most abundant fragment ions are b4 & y8, which is caused by the Asp(D)-Pro(P) cleavage - the weakest peptide bond of all. I am therefore confident that the interpretation made by the authors is correct.

However, this identification of HSP70-K77 acetylation is based on in-vitro acetylation and have never been identified in-vivo,- although lots of in-vivo acetylation sites on HSP70 have been described previously from large-scale proteomics experiments (see for example: Choudhary et al, Science 2009 and Lundby et al, Cell Reports 2012). On the other hand, K77 has previously been identified as a ubiquitylation site (ref. Wagner SA, et al. Mol Cell Proteomics 2011), so the K77R and K77Q mutants would also prevent and interfere with this regulatory PTM! This should be considered by the authors.

Reviewer #5 (Remarks to the Author):

In this manuscript, the authors investigated the role and interplay of Hsp70 and ARD1 on protein folding/degradation and cell survival under stress conditions.

As I am not really an expert in this specific field, I did not review in details most of this manuscript. Nonetheless, I am impressed by the quantity of experiments and techniques used.

In regard to the in vivo part, I have however several concerns as I found this part poorly described and not really clear.

1. For example, they said in page 15 that "Hsp70 K77R mutation reduced the zebrafish survival ratio (Supplementary Fig. 7a)". This sentence does not make sense as it is presented. One can understand that injection of Hsp70 RNA presenting K77R mutation would reduce zebrafish survival in any conditions (stress or no stress). They do not present non-injected embryos survival rate, thus they cannot make this claim. I suppose they actually wanted to say that Hsp70 K77 mutation reduced the protective effect of HSP70. If it is the case they should still present survival ratio of non-injected embryos to evaluate the protective effect of HSP70 RNA injection. On top of that, no statistics are presented for those experiments. In theory, they should repeat several times this work and present some sort of statistical analysis. Without that, the authors cannot make any conclusions or claims. This is purely descriptive.

2. All of the in vivo experiments described in this manuscript are based on RNA injections.

a. It is not clear if they produced themselves the plasmids pEGFP-C3-Hsp70 and pCS2+-ARD1 presented in the method section; I guess so. They should clarify that and, if they did produce these plasmids, they should present the cloning steps. Moreover, which material did they use to produce the mutated RNA forms? I may miss something here.

b. They did not confirm if the injected RNA was properly translated. It would be important to see some sort of confirmation of the presence of the corresponding protein. They developed specific antibodies in this study, so it might be a quick validation procedure (They need to test the antibody on non-injected fish too). Moreover, they analysed the effect of such injections in animals up to 5dpf. Injected RNA is most likely fully degraded at 5dpf. So it would be interesting to see if the protein is still present or when it starts to decline. It is interesting to see if such injection have a long lasting protective effect as they seem to show.

3. On the same page, authors said "Functionally, the K77R mutation markedly decreased zebrafish locomotor activity compared with that of wild-type Hsp70 (Supplementary Fig. 7e), and expression of dominant-negative mutant ARD1 eliminated the distinguishable Hsp70 effects (Supplementary Fig 7f)." This is not clear and they should review their statement. What the distinguishable Hsp70 effects ? They did not show any non-injected fish, so what is the Hsp70 effect compared to ?

4. Figures S7A and S7B. How old are those embryos/larvae and when the treatment was done? I cannot find these important informations.

5. They used different chemical treatments to induce stress/death in zebrafish. They should present references to previous descriptive experiments.

6. Figure S7E and S7F. Authors should state in the legend how old are those fish (it is presented in the method section but it is not convenient for the reader). These experiments were performed in dark or light condition?

7. Figure S7A legend. Authors said "the survival ratios of zebrafish were measured by western blotting." I guess they forgot to review this sentence...

8. Figure S7(C and D). Authors said "were immunostained (left) and counted (light) (c)." Does light should be right?

Responses appear below the reviewers' comments

Reviewer #1:

The manuscript describes a previously unknown mechanism of Hsp70 regulation by post-translational lysine acetylation. It is proposed that stress induced Hsp70 is acetylated on Lys77 upon stress, which increases its chaperone activity and binding to Hsp90 and cofactor Hop. Later on the acetylation is lost and more binding to the cofactors Hsp40 and the E3 ubiquitin ligase CHIP is observed. Hsp70 acetylation is by ARD1, and deacetylation by HDAC4. The Lys77 site is Arg77 in Hsc70 which therefore does not have such regulation. The non-modifiable Hsp70 mutants K77R and acetylation mimic K77Q recapitulate the cofactor interactions. K77R but also K77Q is less protective than WT Hsp70, and it is proposed that an acetylation regulated switch from folding to degradation is important for Hsp70 in the response to stress.

The mechanism, if correct, is a novel finding and will be of broad general interest. As the authors point out, it would provide a fast response to stress independent of the transcriptional response. While a number of post-translational modifications of Hsp70 have been reported, many of their functional effects have been uncertain.

There are, however, a number of serious technical concerns that have to be addressed before publication can be recommended. The most important (point 1) is the use of cells and zebrafish which are mostly dead or dying. There are also questions about some of the in vitro and functional experiments. Because a link between the acetylation site and cofactor binding site is unknown, a high standard of data is required.

1. The stress conditions used, typically 1 mM peroxide for up to 24 h, is harsh enough that there are doubts about physiologic relevance of the late phase loss of acetylation. The model (Fig. 7) indicates time after stress suggesting recovery, but in reality the experiments address the time/harshness of stress with no recovery phase. In Fig. 6, between 40% and 70% of SH-SY5Y cells are dead after treatment. It is harder to make firm conclusions about the biology of cells when disruption is this extreme. Indeed, the harshness of the treatment was reduced to 0.3 mM peroxide to detect apoptosis markers. At higher

peroxide levels it is possible that cell death is more due to broad mechanical disruption by irreversible oxidative damage.

If the acetylation-deacetylation mechanism contributes to cellular function over a range of stress conditions, it should also be observable in cells which are recovering from shorter stresses. There is evidence in supplemental Fig. 1c, where cells are treated with 1 mM peroxide for 1 h then allowed to recover for 24 h, and the acetylation change is detected. Key experiments should be re-done using similar stress and recovery experiments.

We appreciate the reviewer's valuable comments on our manuscript. Regarding the concentration used for hydrogen peroxide treatment, we previously observed that Hsp70 acetylation and its association with Hop were gradually increased by hydrogen peroxide in a dose-dependent manner (Revision Letter Fig. 1). Based on this preliminary experiment, we treated the cells with 1 mM hydrogen peroxide because this condition most strongly induced Hsp70 acetylation and Hop binding to Hsp70.

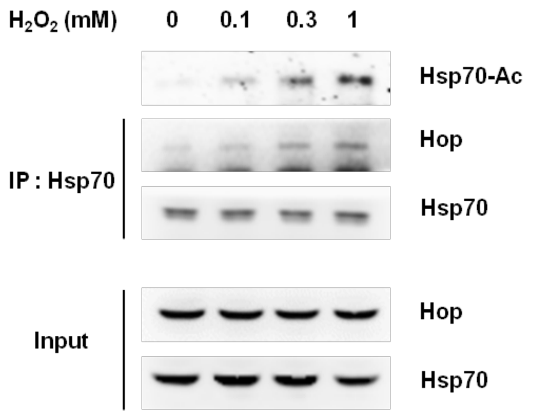

Revision Letter Figure 1. Hydrogen peroxide induces Hsp70 acetylation and association of Hsp70 with Hop in a dose-dependent manner. After treating HEK293T cells with H_2O_2 for 1 h, acetylation of endogenous Hsp70 was analyzed by immunoprecipitation using an anti-Lys-Ac antibody. Endogenous Hsp70 was precipitated using an anti-Hsp70 antibody, and its association with Hop was determined by western blotting.

However, as the reviewer mentioned, we were also concerned that the harsh stress condition could disrupt the broad cellular mechanisms in the late phase after cellular stress. Thus, we also exposed the cells to a shorter duration of stress rather than constant stress and observed the same pattern in the change of Hsp70 acetylation level and its co-chaperone bindings (Supplementary Fig. 1c).

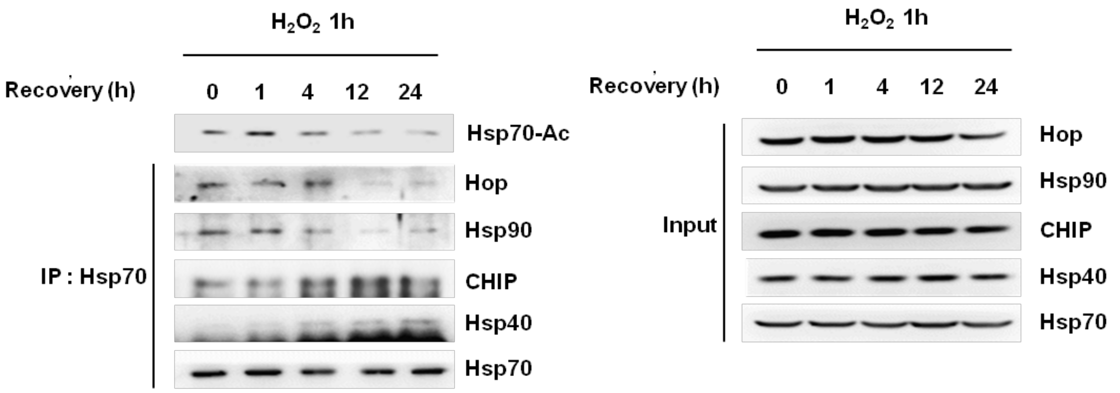

Supplementary Figure 1c. After brief expose of cellular stress, Hsp70 gradually alters its acetylation level and co-chaperone binding partners. HEK293T cells were treated with 1 mM H_2O_2 for 1 h. During the recovery time, the temporal changes in acetylation and co-chaperone binding of endogenous Hsp70 were assessed by immunoprecipitation and western blotting.

In addition, as suggested by the reviewer, we also repeated other key experiments under similar conditions. For this, HEK293T cells were treated with H_2O_2 for 1 h and then washed with phosphate-buffered saline to remove H_2O_2. During the recovery time after shorter stress, we first examined the switching of co-chaperone binding using wild-type and K77R and K77Q mutant Hsp70. As shown in Revision Letter Fig. 2, wild-type Hsp70 switched its binding partner from Hop in the early recovery phase (2 h) to CHIP in the late recovery phase (20 h). However, K77R and K77Q mutant Hsp70 could not change its binding partner under the same conditions. Consistent with their acetylation states, the K77R mutant and K77Q mutant bound to CHIP and Hop, respectively. These data indicate that depending on the acetylation status of K77 residue, Hsp70 switches its function from protein refolding to protein degradation during the recovery phase after cellular stress.

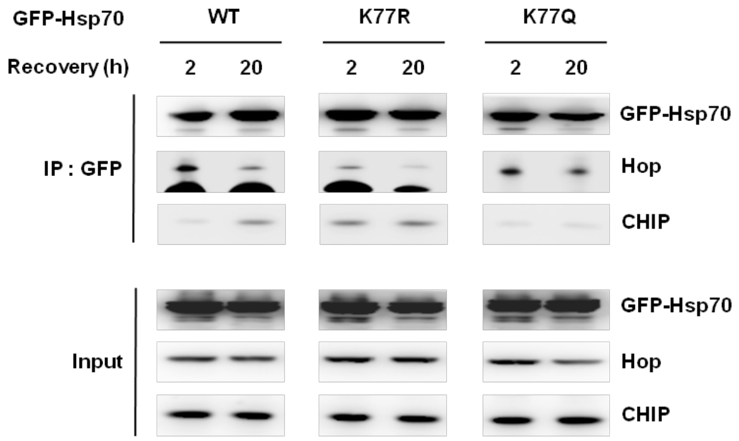

Revision Letter Figure 2. During the recovery time after stress, Hsp70 switches its co-chaperone binding depending on the acetylation/deacetylation state of K77 residue. HEK293T cells expressing GFP-Hsp70 WT, K77R and K77Q were treated with 1 mM H_2O_2 for 1 h, followed by washing with phosphate-buffered saline. After 2 h and 20 h, co-chaperone binding of GFP-Hsp70 was determined by immunoprecipitation using an anti-GFP antibody.

Next, to determine the role of temporal switching of Hsp70 function in the maintenance of protein homeostasis against cellular stress, we evaluated protein refolding during the early recovery phase (2 h) after shorter stress. As shown in Revision Letter Fig. 3, compared to wild-type Hsp70, the K77R mutant showed defective protein refolding in the early recovery phase, while the K77Q mutant exhibited no change. These data indicate that acetylation of Hsp70 is required for protein refolding in the early recovery phase after shorter stress.

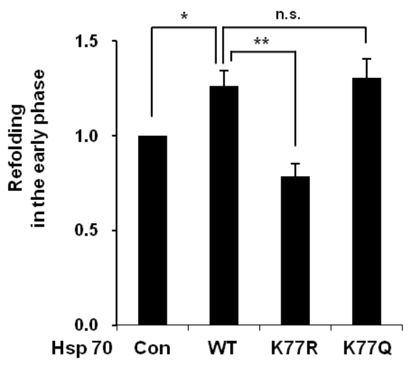

Revision Letter Figure 3. Acetylated Hsp70 induces protein refolding in the early phase after stress. K77R mutation of Hsp70 impaired protein refolding in the early recovery phase after stress. HEK293T cells were transfected with pRSV-luciferase and the indicated Hsp70 plasmids. After luciferase unfolding was induced by treatment with 0.3 mM H_2O_2 for 30 min, the cells were recovered for 2 h and the firefly luciferase activity was measured. Error bars indicate S.D. (n = 3). $^{*}P < 0.01$; $^{**}P < 0.005$, *t* test. n.s.; not significant.

We also evaluated protein degradation in the later recovery phase (20 h) after shorter stress. We analyzed ubiquitination-dependent protein degradation using western blotting. As shown in Revision Letter Fig. 4, the total amount of ubiquitinated proteins was decreased by K77Q mutation, but not by K77R mutation of Hsp70. These results indicate that deacetylated Hsp70 promotes protein degradation in the later recovery phase after shorter stress.

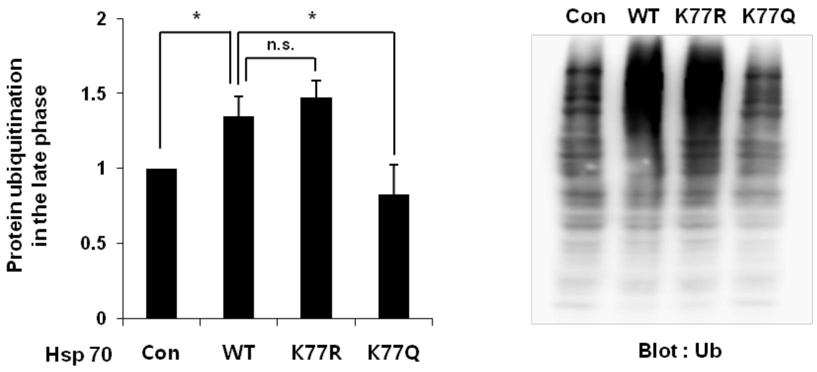

Revision Letter Figure 4. Deacetylated Hsp70 controls protein ubiquitination in the late phase after stress. K77Q mutation of Hsp70 impaired protein degradation in the late recovery phase after stress (20 h). HEK293T cells were treated with 1 mM H_2O_2 for 1 h, and then washed with phosphate-buffered saline. After 20 h, total protein ubiquitination was analyzed to estimate the amount of protein degradation. Protein ubiquitination was measured by western blotting using an anti-ubiquitin antibody (right) and quantified (left).
Error bars indicate S.D. (n = 3). **P* < 0.05, *t* test. n.s.; not significant.

Finally, to examine the effect of functional switching of Hsp70 from protein refolding to degradation on final protein quality after stress, we measured the accumulation of protein aggregation at 24 h after shorter stress. As shown in Revision Letter Fig. 5, protein aggregation was increased by both the K77R and K77Q mutations in Hsp70, indicating that acetylation/deacetylation-induced temporal switching of Hsp70 function from protein refolding to protein degradation during the recovery time after stress is essential for the control of protein quality against cellular stress.

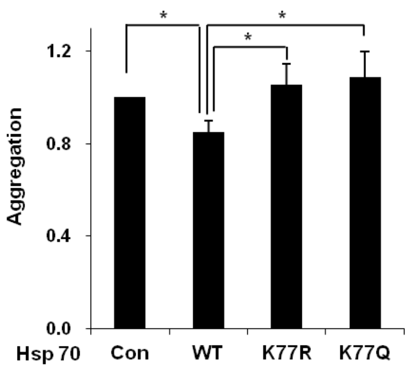

Revision Letter Figure 5. The switch between Hsp70 acetylation/deacetylation is required to maintain protein homeostasis after stress. Stress-induced protein aggregation was enhanced by both the K77R and K77Q mutations in Hsp70. HEK293T cells were treated with 1 mM H_2O_2 for 1 h, and then washed with phosphate-buffered saline to remove H_2O_2. After 24 h, the accumulation of protein aggregation was analyzed using protein aggregation detection dye.
Error bars indicate S.D. (n = 3). *$P < 0.05$, *t* test. n.s.; not significant.

In conclusion, the pattern of the results in all experiments performed under recovery conditions after shorter stress was consistent with previous results. Therefore, these data confirm the contribution of the K77 acetylation/deacetylation mechanism of Hsp70 to cellular function over a range of stress conditions.

2. The stress conditions used were all related to oxidation. Is the acetylation also observed under other conditions that cause the transcriptional heat shock response such as heat or Hsp90 inhibition? Related to this, a heat shock response is not observed in the HEK293T cells with peroxide, and another stress that causes the response could be used to show acetylation of endogenous Hsp70.

As the reviewer suggested, we previously investigated the change in acetylation level of Hsp70 in HEK293T cells after heat shock. As shown in Revision Letter Fig. 6, after heat shock, Hsp70 acetylation was increased and then Hsp70 was gradually deacetylated. However, the heat shock response of Hsp70 was somewhat different from that after other types of stress. The heat shock response of Hsp70 was slower than the responses to other types of stress. In particular, heat shock-induced Hsp70 acetylation began increasing after 4 h and reached a peak at 24 h (Revision Letter Fig. 6), whereas other stress, such as hydrogen peroxide, etoposide and MPP+, rapidly increased Hsp70 acetylation in the earlier phase after stress (1–4 h) (Fig. 1 and Supplementary Fig. 1). Moreover, unlike other cellular stress, heat shock increased the expression levels of Hsp40 and CHIP (Revision Letter Fig. 6); thus, heat shock treatment appears to not be appropriate for the quantification of co-chaperone binding changes after stress. Nevertheless, heat shock-induced acetylation of Hsp70 was also correlated with its binding to Hop and Hsp90 (Revision Letter Fig. 6), confirming that the acetylation/deacetylation state of Hsp70 modulates its co-chaperone binding after cellular stress.

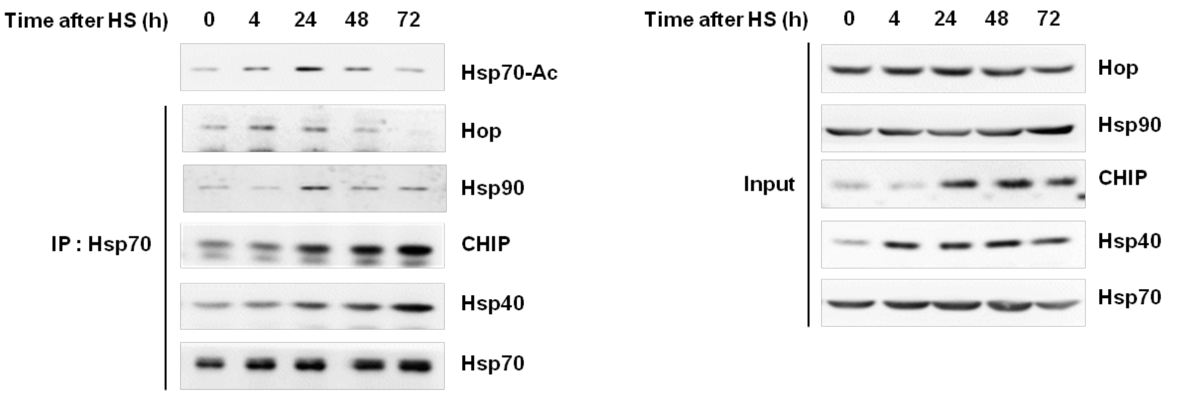

Revision Letter Figure 6. Heat shock induces Hsp70 acetylation and modulates co-chaperon bindings to Hsp70. HEK293T cells were treated with 42 °C heat shock for 30 min. During the recovery time, the acetylation level of endogenous Hsp70 was determined by immunoprecipitation using an anti-Lys-Ac antibody. For co-chaperone binding, endogenous Hsp70 was immunoprecipitated using an anti-Hsp70 antibody, and then its binding partners were analyzed by western blotting. HS; heat shock.

Regarding the reviewer's question about the absence of a heat shock response under our conditions, the constant expression of Hsp70 and co-chaperones was required for quantitative comparison of their binding. For this purpose, we used HEK293T cells, which have been reported to maintain a consistently high level of Hsp70 regardless of the presence of stresses were used[1, 2]. For the same reason, we could not use heat shock stress that cause a transcriptional change of co-chaperones, such as CHIP and Hsp40 (Revision Letter Fig. 6).

However, we agree with the reviewer's comment that the stress conditions used in our study are related to oxidation. Thus, we treated the cells with other types of cellular stress, NaCl and EtOH. When the cells were treated with NaCl and EtOH, the acetylation of Hsp70 was increased in the early phase after stress (2 h), after which Hsp70 was deacetylated again in the late phase after stress (20 h) (Revision Letter Fig. 7a and b). Consistent with the Hsp70 acetylation/deacetylation state, Hsp70 showed preferential binding to Hop in the early phase (2 h) and CHIP in the late phase (20 h) after stress. These results indicate that acetylation/deacetylation of Hsp70 is not due to cellular oxidative conditions and is a conserved mechanisms shared by various types of cellular stress.

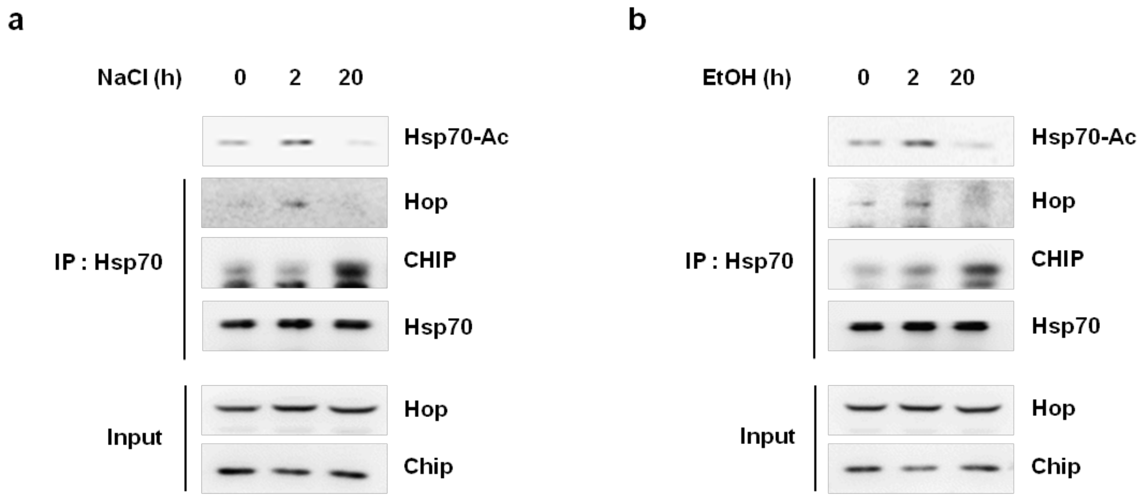

Revision Letter Figure 7. Stress-induced Hsp70 acetylation is related to binding of its co-chaperones. HEK293T cells were treated with 100 mM NaCl (a) and 150 mM EtOH (b) for the indicated time. Acetylation of endogenous Hsp70 was accessed by immunoprecipitation using an anti-Lys-Ac antibody. For co-chaperone binding, endogenous Hsp70 was immunoprecipitated using an anti-Hsp70 antibody and its binding to Hop and CHIP was analyzed by western blotting.

3. In Fig. 2c, the acetylation state of Hsp70 in the HEK293T cells with knockdown of ARD1 must also be shown. It is only shown for SH-SY5Y in Fig. 2b.

As suggested by the reviewer, we addressed the acetylation level of Hsp70 in HEK293T cells by knocking down ARD1. Similar to the results for SH-SY5Y (Fig. 2b), hydrogen peroxide-induced Hsp70 acetylation was significantly diminished in ARD1-deficient HEK293T cells (Fig. 2c). Accordingly, increased binding of Hop and Hsp90 to Hsp70 after stress induction was also reduced in ARD1-deficient cells. These results have been incorporated into Fig. 2c in the revised manuscript.

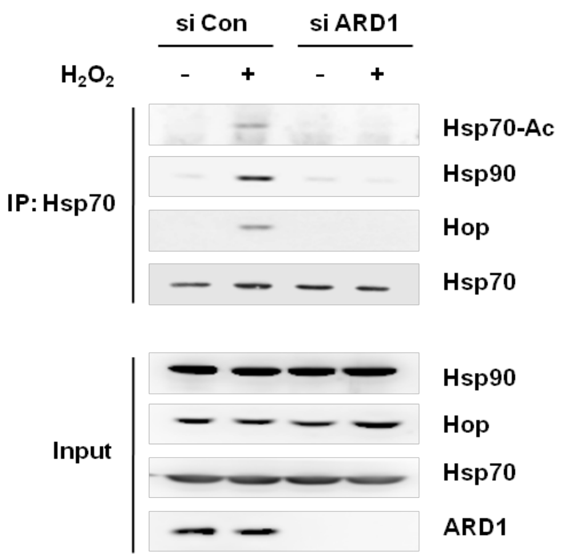

Figure 2c. ARD1 regulates the acetylation and co-chaperone bindings of Hsp70. HEK293T cells were trasnfected with ARD1 siRNA. After cells were treated with 1 mM H_2O_2 for 1 h, endogenous Hsp70 was precipitated using an anti-Hsp70 antibody, and then its acetylation level and co-chaperone binding were analyzed by western blotting.

4. The lysine-directed activity of ARD1 has been in question because of its identification as N-terminal acetyltransferase, most recently in Magin et al. J Biol Chem. 2016 291(10):5270-7. The paper reported non-enzymatic chemical acetylation of lysines by acetyl-CoA in the absence of ARD1, that was less efficient than ARD1-dependent N-terminal acetylation. The experiments in Fig. 3a indicate that true lysine acetylation is observed. To make this even stronger, the negative control reaction lacking ARD1 should be repeated for full-length Hsp70 in the time course, Fig. 3c.

As recommended by the reviewer, we repeated the *in vitro* Hsp70 acetylation assay with a negative control group reacted without the ARD1 recombinant. In the absence of the ARD1 recombinant, acetylation of Hsp70 was not increased *in vitro* (Fig. 3c). These results have been incorporated into Fig. 3c in the revised manuscript.

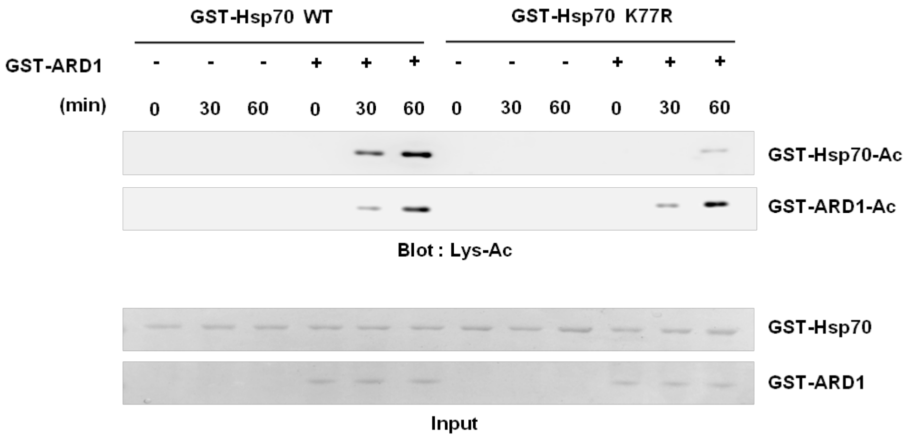

Figure 3c. ARD1 acetylated K77 of Hsp70 *in vitro*. GST-Hsp70 WT and GST-Hsp70 K77R recombinants were subjected to *in vitro* acetylation assays with or without GST-ARD1 recombinant. Acetylation levels of GST-Hsp70 recombinants were determined using an anti-Lys-Ac antibody.

Furthermore, to find further evidence of the lysine acetylation activity of ARD1, we denatured the ARD1 recombinant by heat treatment to disrupt its enzymatic activity, and then performed an acetylation assay. As shown in Revision Letter Fig. 8, the Hsp70 recombinant was not acetylated by the denatured ARD1 recombinant. This result indicates that the increase in Hsp70 acetylation *in vitro* is not a non-enzymatic chemical reaction, but true lysine acetylation mediated by ARD1.

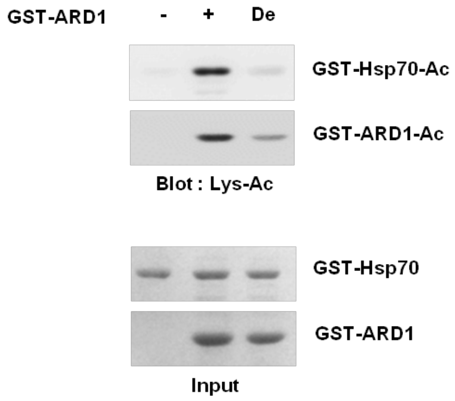

Revision Letter Figure 8. Enzymatic activity of ARD1 is required for Hsp70 acetylation *in vitro*. GST-ARD1 and GST-Hsp70 recombinants were subjected to *in vitro* acetylation. For protein denaturation, the ARD1 recombinant was incubated at 95°C for 10 min before the acetylation assay. De; denatured recombinant.

5. In Fig. 4c, the unstimulated ATPase activity of Hsp70 is reported in relative terms. However, in cells Hsp70 is constantly interacting with Hsp40/J-domain proteins and nucleotide exchange factors which stimulate its ATPase rate. To better represent the physiological state, rates in the presence of these cofactors (eg. Hsp40 and Hsp105) should be tested.

As the reviewer suggested, we analyzed the ATPase activity of Hsp70 in the presence of its co-factor Hsp40/J-domain protein. As shown in Fig. 4c, the ATPase activity of Hsp70 was increased when Hsp70 was acetylated by ARD1 *in vitro*. However, according to its acetylation level, the ATPase activity of the K77R mutant Hsp70 and Hsc70 was not changed by ARD1 (Fig. 4c). We have incorporated this data into Fig.4c in the revised manuscript.

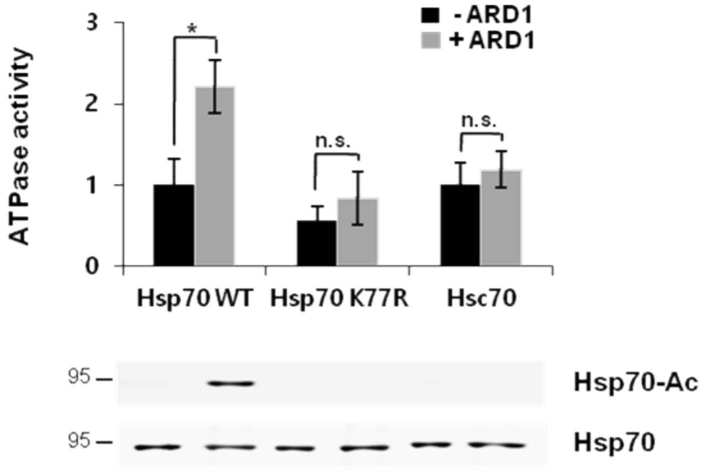

Figure 4c. K77 acetylation upregulates the ATPase cycle of Hsp70. GST-Hsp70 and GST-Hsc70 recombinant proteins were subjected to *in vitro* acetylation assay with or without ARD1. For ATPase activity assay, recombinant proteins were incubated with His-Hsp40 recombinant proteins and ATP for 1 h, then ATP hydrolysis was measured.

Error bars indicate S.D. (n = 3). **P* < 0.05, *t* test. n.s; not significant.

In addition to the *in vitro* experiment, we also evaluated the ATPase activity and ATP-binding affinity of the wild-type and mutant Hsp70 in cells, in which Hsp70 is interacting with co-factors (Supplementary Fig. 6b and c). Consistent with the *in vitro* data, the ATPase activity and ATP-binding affinity of Hsp70 was decreased by the K77R mutation. These results support that K77 acetylation of Hsp70 regulates its chaperone activity in the physiological state.

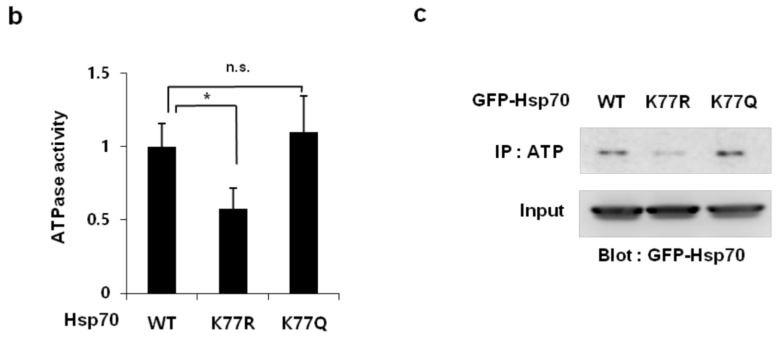

Supplementary Figure 6b and c. K77 acetylation is required for ATPase cycles of Hsp70 in cells. GFP-Hsp70 WT, K77R and K77Q were transfected into HEK293T cells. GFP-Hsp70 WT, K77R and K77Q were precipitated and the ATPase activities were measured (b). ATP binding affinities of GFP-Hsp70 WT, K77R and K77Q were analyzed by immunoprecipitation using ATP-agarose beads (c).

Error bars indicate S.D. (n = 3). *$P < 0.05$, *t* test. n.s.; not significant.

6. The protein concentrations in the in vitro experiments are not given (ATPase assay, acetylation, Hop and CHIP binding, ATP-agarose binding). This is very important for the experiment showing competition between Hop and CHIP, Fig. 5b, to evaluate the relative strength of binding. Because the antibodies are different, the blots do not report on the absolute amounts of each protein in the inputs.

In vitro experiments, we used 0.5–1.0 μg recombinant proteins for acetylation assay. For ATPase activity assay and ATP-agarose binding assay, we used 1 μg and 0.1 μg recombinant proteins, respectively. For competitive binding of Hop and CHIP, similar molar concentration of Hop and CHIP recombinant proteins were subjected into *in vitro* binding assay; 1 μg Hop recombinant proteins and 0.5 μg CHIP recombinant proteins were incubated with 1 μg GST-Hsp70 recombinant proteins. As suggested by the reviewer, we have incorporated this information into the Methods section of the revised manuscript.

7. The luciferase refolding in Fig. 5e is in question. The procedure is very different from the reference cited, Ran et al. Cell Stress Chaperones. 9, 229-242 (2004). In that paper, luciferase expressed in cells was denatured by a 45{degree sign}C heat stress followed by 3 h of refolding at 37{degree sign}C. This is similar to methods used by other groups (eg. Nollen et al. PNAS 2001 98(21):12038-4). Peroxide is not

used for these experiments. One reason is that heat denaturation is reversible except for aggregation, but protein damage by oxidation is mostly irreversible. It seems surprising that there is any significant regain of activity, from this viewpoint. The authors should use the established heat stress methods, or include more data to establish the peroxide method. For example, how much luciferase activity is refolded compared to before denaturation, what the are levels of soluble luciferase before and during refolding, is refolding inhibited by Hsp70 knockdown or inhibition, what are refolding levels without Hsp70 overexpression.

As pointed out by the reviewer, most luciferase refolding assays use the heat denaturation method. Previously, we first performed the assay using heat denaturation. Because heat induced Hsp70 acetylation beginning at 4 h (Revision Letter Fig. 6), we measured the luciferase activity at 4 h after heat shock and found that the K77R mutation of Hsp70 impaired protein refolding (Revision Letter Fig. 9a). Although we obtained significant results for heat shock, 4 h was still not appropriate because heat-induced acetylation was relatively weak at this time point and peaked at approximately 24 h. Furthermore, at 4 h after heat shock, K77Q mutant Hsp70 showed higher refolding activity than wild-type Hsp70, possibly because of insufficient acetylation of wild-type Hsp70. To fully reflect the acetylation-mediated changes in refolding activity, protein refolding should be analyzed at approximately 24 h, but this is too late for the luciferase refolding assay, for which the typical assay time is 0–2 h. Thus, we concluded that this method is not suitable for our experiment and conducted the assay using hydrogen peroxide.

To optimize the experimental conditions, we first used different peroxide concentrations from 0.1 to 1 mM and found that 30 min treatment with 0.3 mM peroxide decreased the activity to ~40%, which recovered to ~80% in 2 h (Revision Letter Fig. 9b). During the assay, protein synthesis was blocked with cyclohexamide and the soluble luciferase level was not significantly changed (Revision Letter Fig. 9c). As the reviewer suggested, to further verify our experimental conditions, we treated the cells with an Hsp70 inhibitor, VER-155008, and observed its inhibitory effect (Revision Letter Fig. 9d). Additionally, overexpression of Hsp70 enhanced protein refolding compared to the control, and the ability of Hsp70 to promote protein refolding was blocked by its K77R mutation (Revision Letter Fig. 9e). Therefore, this method is suitable for our experiment. As the reviewer commented, we performed the analysis with a control and indicated the recovery level relative to the control; these data have been incorporated into Fig. 5e in the revised manuscript.

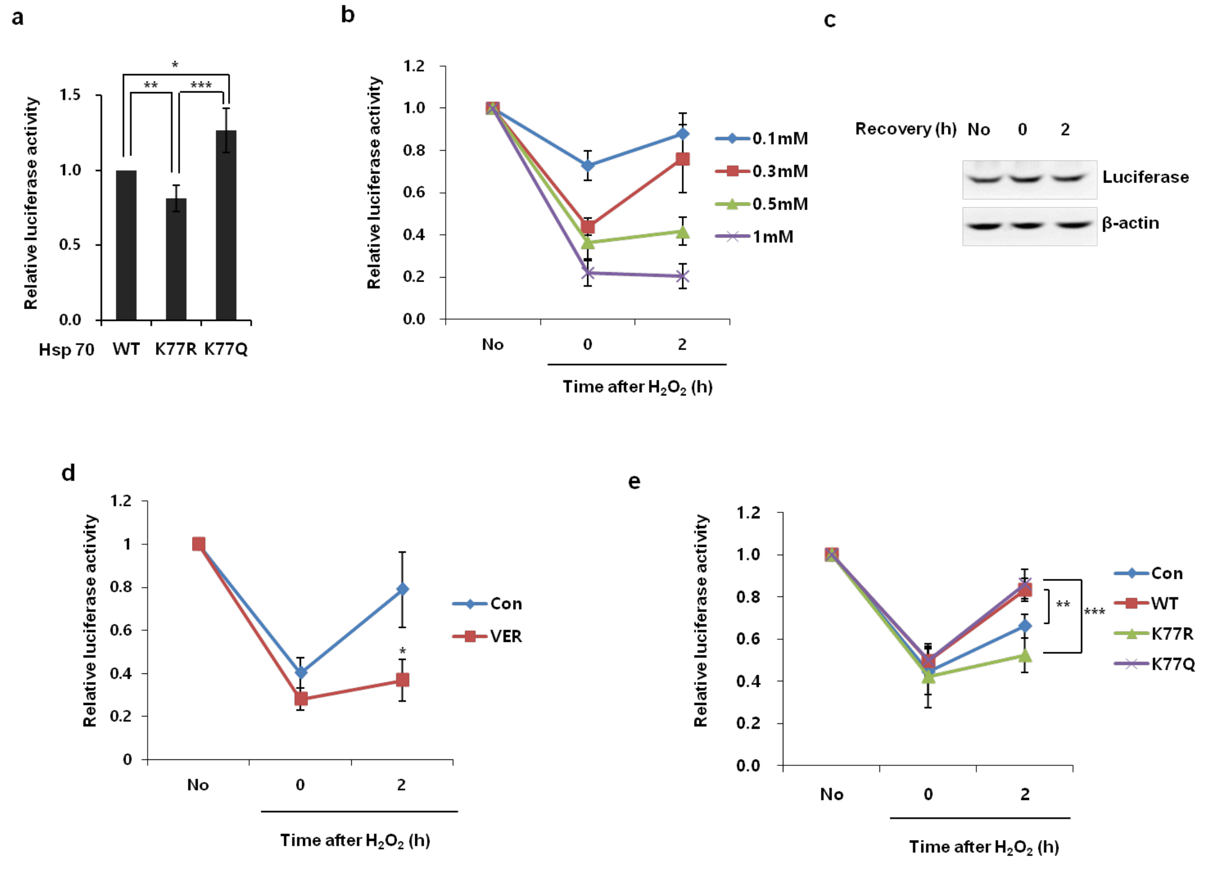

Revision Letter Figure 9. Luciferase assay was performed with heat shock and hydrogen peroxide.

(a) HEK293T cells were transfected with the indicated Hsp70 plasmids. After treatment at 42 °C heat shock for 30 min, the cells were recovered for 4 h, and then protein refolding was measured.

(b) After the HEK293T cells were treated with the indicated concentration of H_2O_2 for 30 min, the cells were recovered for 2 h and then protein refolding was measured.

(c) HEK293T cells were treated with 0.3 mM H_2O_2 for 30 min and then the cells were recovered for 2 h. Cell lysates were extracted and the total amount of soluble luciferase was analyzed by western blotting.

(d) HEK293T cells were pretreated with Hsp70 inhibitor, VER-155008, for 30 min before H_2O_2 stimulation. After treatment with 0.3 mM H_2O_2 for 30 min with or without VER-155008, the cells were recovered for 2 h and protein refolding was measured.

(e) HEK293T cells were transfected with the indicated Hsp70 plasmids. After treatment with 0.3 mM H_2O_2 for 30 min, the cells were recovered for 2 h and protein refolding was measured.

Error bars indicate S.D. (n = 3). **P* < 0.05; ***P* < 0.01; ****P* < 0.005, *t* test. No; untreated, VER; VER-155008.

8. Fig. 5f claims to represent protein degradation but it actually shows total protein ubiquitination after proteasome inhibition. This is related but is not a direct measure of degradation. Images of the blots should be shown. For Fig. 5g, aggregation at the early phase should also be shown. Fig. 5e and f are lacking controls without overexpressed Hsp70.

We agree with the reviewer's comment that total protein ubiquitination is not a direct measure of protein degradation. Therefore, we corrected the y-axis label of graph in Fig. 5f from "protein degradation in the late phase" to "protein ubiquitination in the late phase". We have also explained that we measured total protein ubiquitination to estimate the amount of protein degradation in the figure legend. In addition, as the reviewer suggested, we have incorporated the western blotting image for protein ubiquitination into Fig. 5f. We have also added the control group without overexpressed Hsp70 to Fig. 5e–g.

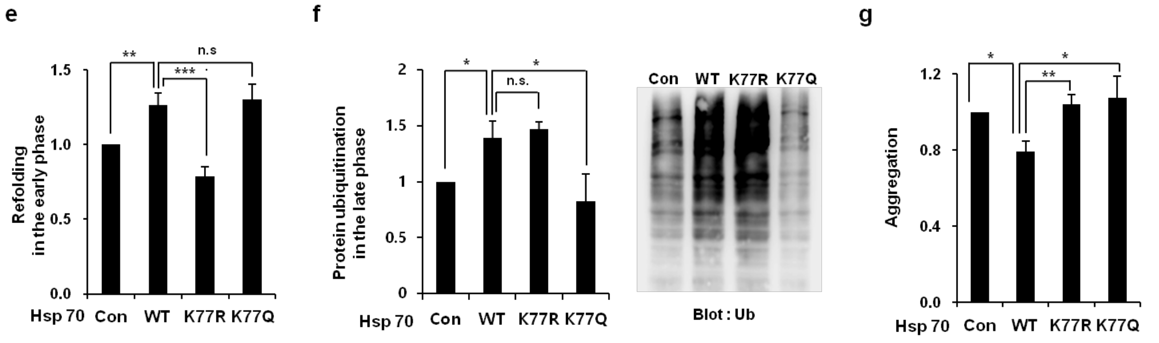

Figure 5e–g. K77 acetylation temporally balances the chaperone functions of Hsp70 between protein refolding and degradation after stress.

(e) Acetylated Hsp70 encourages protein refolding in the early phase after stress. The K77R mutation of Hsp70 impaired protein refolding in the early phase after stress (2 h). HEK293T cells were transfected with pRSV-luciferase and the indicated Hsp70 plasmids. After luciferase unfolding was induced by treatment with 0.3 mM H_2O_2 for 30 min, the cells were recovered for 2 h and firefly luciferase activity was measured.

(f) Deacetylated Hsp70 controls protein ubiquitination in the late phase after stress. K77Q mutation of Hsp70 impaired protein degradation in the late phase after stress (20 h). To estimate the amount of protein degradation, total protein ubiquitination was analyzed. After HEK293T cells were treated with 1 mM H_2O_2 for 20 h, total protein ubiquitination was measured by western blotting using an anti-ubiquitin antibody (right) and quantified (left).

(g) The switch between Hsp70 acetylation/deacetylation is required to maintain protein homeostasis after stress. Stress-induced protein aggregation was enhanced by both the K77R and K77Q mutations of Hsp70. HEK293T cells expressing the indicated Hsp70 plasmids were treated with 1 mM H_2O_2 for 24 h. Using cell extracts, protein aggregation was analyzed by protein aggregation detection dye.

Error bars indicate S.D. (n = 3). *$P < 0.05$; **$P < 0.01$; ***$P < 0.005$, *t* test. n.s.; not significant.

Regarding the reviewer's comment for Fig. 5g, we analyzed protein aggregation during the early phase after stress. In the early phase (2 h), stress-induced accumulation of protein aggregation was weakly increased by the K77R mutation of Hsp70, but not by the K77Q mutation (Revision Letter Fig. 10). In the early phase after stress, acetylated Hsp70 mainly boosted protein refolding, and thus the acetylation mimic K77Q mutation of Hsp70 might induce no significant defect compared to wild-type Hsp70. In this manuscript, we presented the accumulation of protein aggregation at 24 h as an outcome of the failure of protein refolding in the early phase (2 h) or degradation in the late phase (20 h) after stress; therefore, we did not include the data related to protein aggregation in the early phase in our manuscript.

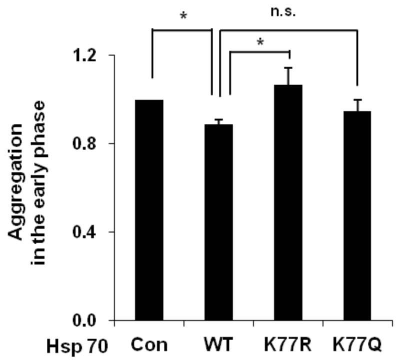

Revision Letter Figure 10. Hsp70 acetylation is required for the maintenance of protein homeostasis in the early phase after stress. Stress-induced protein aggregation was increased by both K77R mutations in Hsp70 in the early phase after stress. After treatment with 1 mM H_2O_2 for 2 h, the accumulation of protein aggregation was analyzed in HEK293T cells.

Error bars indicate S.D. (n = 3). *$P < 0.05$, *t* test. n.s.; not significant.

9. The identification of HDAC4 as the relevant deacetylase in Supplemental Fig. 5 should be supported by knockdown.

As suggested by the reviewer, we knocked down HDAC4 using siRNA and addressed the acetylation level of Hsp70 and its co-chaperone binding in HEK293T cell. As expected, in the late phase after stress induction (20 h), Hsp70 acetylation level was increased and Hsp70 co-chaperone binding was altered accordingly in HDAC4-deficient cells, confirming that HDAC4 is important in Hsp70 deacetylation under stress conditions (Supplemental Fig. 5d). These new results have been incorporated into Supplementary Fig. 5d and described in the revised manuscript **[on page 12_line 250–254].**

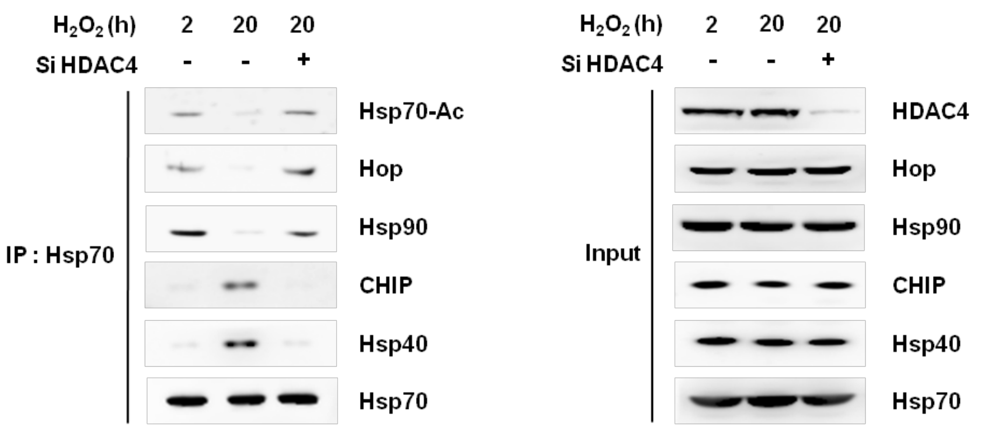

Supplemental Figure 5d. HDAC4 is a relevant deacetylase to K77 acetylation of Hsp70. HEK293T cells transfected with HDAC4 siRNA were treated with 1 mM H_2O_2 for the indicated times. Endogenous Hsp70 was precipitated, and its acetylation and co-chaperone binding was assessed by western blotting.

10. It is suggested that as the acetylation is lost, CHIP binding increases. But in the late phase, much more CHIP is bound than before stress, when Hsp70 is also not acetylated (Fig. 1). The lack of acetylation alone does not account for the increase in CHIP binding. Part of the result may be due to abnormal behavior of proteins cells which are dying. In any case, the authors should suggest some explanation. In cells, phosphorylation of T636 changes binding from CHIP to Hop (Muller et al. Oncogene (2012), 1-10), and this should be tested to rule out such a mechanism.

We have considered this issue from several perspectives. Although we do not have a clear explanation for this, one major factor may be the differing cellular protein qualities before and after stress. Although non-acetylated Hsp70 shows preferential binding to CHIP, non-stressed cells contain relatively fewer misfolded

proteins, for which lower Hsp70 activity is required. The overall level of co-chaperones bound to Hsp70 would be also low under this condition. However, after stress, a large amount of misfolded protein requires higher Hsp70 activity, promoting co-chaperone binding to Hsp70. Therefore, the difference in demand for Hsp70 activity before and after stress will contribute to overall co-chaperone binding, which may cause different CHIP binding under these conditions.

As the reviewer commented, we tested whether phosphorylation of Hsp70 T636, which is reported to regulate Hop/CHIP binding[3], is involved in this process. We over-expressed the non-phosphorylated mimic Hsp70 (Hsp70 T636A) in cells and observed changes in stress-induced co-chaperone binding. Similar to Hsp70 wild-type, CHIP binding to Hsp70 T636A was also increased in the later phase, indicating that phosphorylation does not participate in the control of the later phase of CHIP binding (Revision Letter Fig. 11). Therefore, increased CHIP binding in the later phase compared to at time zero (0 h) may not be related only to acetylation or phosphorylation, but rather a combination of several factors, including post translational modifications, cellular conditions, and other co-factors; thus, further investigation is required.

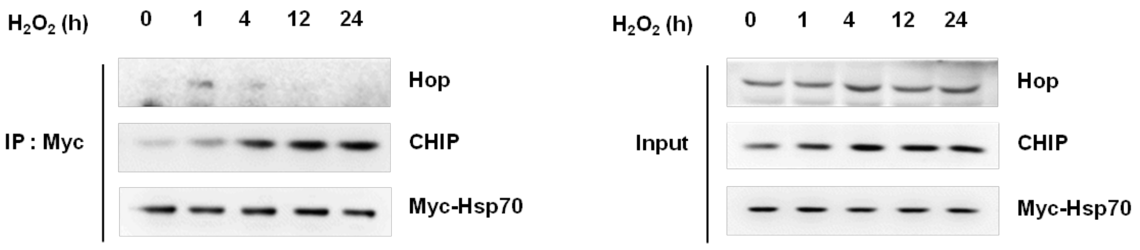

Revision Letter Figure 11. HEK293T cells expressing the Myc-tagged T636A mutant Hsp70 were treated with 1 mM H_2O_2. The Myc-tagged T636 mutant Hsp70 was precipitated using an anti-Myc antibody and its co-chaperone bindings were identified by western blotting

11. I recognize there is no easy way to address how the acetylation site can affect the C-terminal binding site for CHIP and HOP. Still, for a journal of this caliber, a better discussion of the possibilities is expected. On page 17 acetylation "could alter co-factor binding to the SBD", they bind to the flexible extension of the CTD, not SBD, using the terminology of the manuscript. The interactions of the NBD to SBD and CTD require ATP, and the in vitro binding of Hop and CHIP in Fig. 5b are in the absence of ATP, when the NBD does not contact the SBD/CTD.

We appreciate this reviewer's valuable comment. This is a critical point to which we agree and attempted to evaluate. Based on previous studies, the acetylation site K77 is at the interface between NBD and SBD, and thus we predicted that K77 is an important site for the NBD-SBD interaction. However, as the reviewer pointed out, the exchange of co-chaperones occurred even in the absence of ATP when NBD was far from SBD and CTD. We agree with the reviewer's comment that we need to provide more plausible explanations regarding how acetylation influences co-chaperone binding independently of ATP binding.

Without direct interaction, distal domains can be allosterically modulated by co-factors such as nucleotides and ions, disulfide bonds and post-translational modifications[4]. Several studies have shown that phosphorylation-induced allosteric conformational changes modulate protein activity[5, 6, 7]. For example, PKA-mediated phosphorylation of Rap1b induces the conformational change of a domain around the phosphorylation site and the two switch loops in the distal domains involved in effector interaction[6]. Therefore, K77 acetylation in NBD may induce allosteric conformational changes to SBD and CTD, facilitating specific co-chaperone binding. However, detailed structural studies are needed to elucidate the mechanism. As suggested by the reviewer, we changed the previous description in the Discussion section as follows:

"In this study, the Hsp70 acetylation site is located in the NBD, which does not directly interact with Hop and CHIP. Therefore, this modification may affect the C-terminal binding of these proteins through an Hsp70 conformational change. Hsp70 is a representative enzyme regulated by allosteric mechanisms. Without direct interactions, distal domains can be allosterically modulated by co-factors such as nucleotides and ions, disulfide bonds and post-translational modifications[4]. Several studies have shown that phosphorylation-induced allosteric conformational changes modulate protein activity[5, 6, 7]. Thus, K77 acetylation in NBD may induce allosteric conformational changes in SBD and CTD, resulting in changes in co-chaperone binding preference. In addition, K77 is located at a significant position for NBD-SBD interdomain contacts, which adds another interesting possibility that K77 acetylation at the NBD-SBD interface may modulates the Hsp70 conformational change. Although detailed investigations are needed to elucidate the exact mechanisms, our results provide insight into the allosteric mechanism of Hsp70 regulation by post-translational modifications." **[on page 17_line 347–360]**

12. Similarly to point 1., in supplement Fig 7, 30% to 60% of zebrafish are dead after the 1 h treatment with 10 mM peroxide, which seems very harsh. Experiments with milder conditions where closer to 100%

of the animals survive in the control would be better.

As the reviewer commented, we repeated the zebrafish survival experiments under milder conditions. When we reduced the concentration of peroxide from 10 mM to 5 mM, the survival ratio of zebrafish expressing wild-type Hsp70 increased to approximately 85% (Supplementary Fig. 7a and b). We have incorporated these results in the revised manuscript in Supplementary Fig. 7a and b.

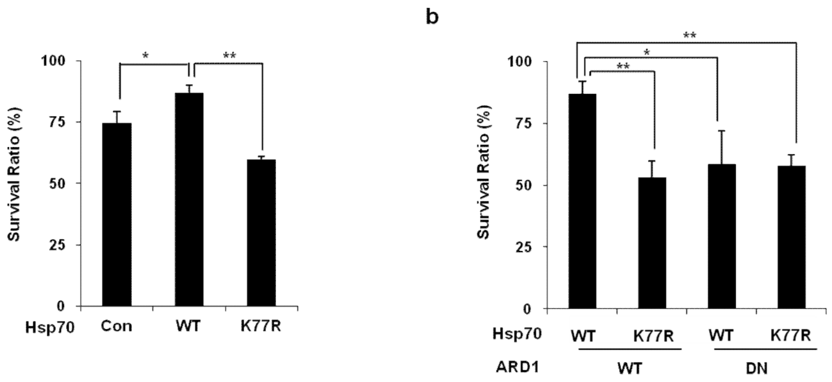

Supplementary Figure 7a and b. ARD1-mediated Hsp70 acetylation protects cells against stress conditions *in vivo.*

(a) Hsp70 acetylation rescues zebrafish lethality. Zebrafish expressing GFP-Hsp70 WT and K77R were incubated with 5 mM H_2O_2 for 1 h at 3 dpf, and the survival ratios of zebrafish were measured on the following day.

(b) ARD1 increases zebrafish survival through Hsp70 acetylation. WT or DN Myc-ARD1 were co-expressed with GFP-Hsp70 in zebrafish. At 3 dpf, embryos were treated with 5 mM H_2O_2 for 1 h, and the survival ratios were measured on the following day.

Error bars indicate S.D. (n = 3). *$P < 0.05$; **$P < 0.005$, *t* test.

Reviewer #2 (Remarks to the Author):

This manuscript reports significant results that describe the dynamics of Hsp70 chaperone-mediated stress response. Moreover, the results present an elegant mechanism that differentiates the behavior of Hsp70 and the very closely related Hsc70 in stress response, sheds light on stress response in transformed/cancer cells in which Hsp70 is present at highly elevated levels in the absence of added external stressors, and provides a mechanism for rapid modulation of stress response that is not based on HSF1-mediated chaperone transcription. In brief, the authors demonstrates that acetylation by ARD1 on a specific residue of Hsp70 (K77) regulates the association with Hop, a pro-folding cochaperone, vs. CHIP, a degradative cochaperone. The kinetics of acetylation and deacetylation following stress allow the chaperone to shape the cell's stress response, resulting in a rapid pro-folding biased response, followed by slower return to pro-degradation.

The manuscript is very clearly written (and indeed, was actually a pleasure to read). Premises/hypotheses are clearly and thoroughly tested and the range of investigation is quite extensive, including experiments in a zebrafish model of stress response. The authors' conclusions about the mechanisms and potential regulatory importance of Hsp70 acetylation by ARD1 are well justified. This is a novel, important and well-conducted study that will be of substantial interest to investigators in the chaperone / protein-quality control field that reveals a compelling new mechanism for cochaperone-switching and for the functional changes that accompany cochaperone-switching.

I have no major objections or critical comments. It would be appropriate for the authors to address one point:

1. It was previously reported that phosphorylation of the Hsp70/Hsc70 tail, including (possibly) by stress-responsive kinases, could also modulate association with Hop vs. CHIP (Muller et al., Oncogene 32(25), 2013; Assimon et al., Biochemistry 54(48), 2015) and that phosphorylation of Hop could inhibit its association with Hsp70 (Röhl et al., EMBO Rep. 16(2), 2015). Could the authors comment on these results and integrate them into their overall model?

We appreciate the reviewer's helpful comments regarding our manuscript. As the reviewer pointed out, it was previously reported that phosphorylation of the C-terminal region Hsp70 modulates its binding to Hop and CHIP and phosphorylation of Hop also affects its affinity to Hsp70 *in vitro*[3, 8, 9]. These pioneering previous studies provided inspiration for the present study and it would be interesting to examine how these modifications react to cellular stress and correlate with K77 acetylation of Hsp70. Although further studies are needed to investigate when these modifications are increased after stress, particularly in the early phase or late phase after stress and whether they can affect the K77 acetylation/deacetylation level of Hsp70 during cellular stress, as the reviewer suggested, we have integrated these points in our model in this manuscript and added some comments to the Discussion section as follows:

"The possibility that protein modification affects co-chaperone binding was also recently suggested in previous reports. Muller *et al.* and Assimon *et al.* showed that phosphorylation of the C-termini of Hsp70 and Hsp90 enhanced their binding to Hop[3, 8]. In addition, Röhl *et al.* reported that phosphorylation of Hop decreased its binding affinity to Hsp70 *in vitro*[9]. It is possible that these modifications are correlated with K77 acetylation of Hsp70 under stress conditions. For example, Hsp70 may be phosphorylated in the early phase after stress and synergize the effect of acetylation of Hsp70 to promote Hop binding. In contrast, phosphorylation of Hop may be stimulated during the late phase after stress to become separated from deacetylated Hsp70. Therefore, further studies are needed to investigate how these post-translational modifications are sequentially stimulated over time after stress induction and how they interact with each other to regulate Hsp70 function." **[on page 16_line 335–page 17_line 346]**

A few very minor points:

1. Page 3, "the extensive NBD surface interacts with the SBD" should probably be "an extensive NBD surface interacts with the SBD"

As the reviewer pointed out, we corrected the sentence in our manuscript as follows:

From "When ATP binds to the NBD, the extensive NBD surface interacts with the SBD, and peptides can

rapidly bind to and be released from the SBD."

To "When ATP binds to the NBD, an extensive NBD surface interacts with the SBD and peptides can rapidly bind to and be released from the SBD. **[on page 3_line 62–63]**"

2. Page 6 "Because the chaperone function of Hsp70 is decided by its co-chaperone" - this seems an odd way to phrase what the authors are trying to convey.

As the reviewer pointed out, to clearly convey our meaning, we have changed the sentence as follows:

From "Because the chaperone function of Hsp70 is decided by its co-chaperone, we first examined the changes of co-chaperone binding with Hsp70 after stress induction to investigate the interplay of Hsp70 function between protein refolding and degradation."

To "To investigate the interplay of Hsp70 function between protein refolding and degradation, we first evaluated the temporal change of Hsp70 function over time after stress. Because the opposing chaperone functions of Hsp70 between protein refolding and degradation are determined by the competitive binding of co-chaperone proteins, such as Hop and CHIP, we analyzed the changes in co-chaperone binding to Hsp70 over time after stress induction." **[on page 6_line 98–102]**

3. Page 11, lower section - "we next determined whether the constituency of the the Hsp70 co-chaperone complex" - the authors may want to use "composition" rather than "constituency" here.

Regarding the reviewer's comment, we replaced "constituency" with "composition" in the sentence as follows:

From "Therefore, we next determined whether the constituency of the Hsp70 co-chaperone complex is regulated by K77 acetylation."

To "Therefore, we next determined whether the composition of the Hsp70 co-chaperone complex is regulated by K77 acetylation." **[on page 11_line 228–229]**

4. Page 18, near top - "therefore, denatured proteins can be excessively regulated by the protein degradation pathway" - it is not clear what the authors mean by "excessively" here - perhaps they mean "more extensively" or "more strongly"?

As the reviewer pointed out, we have replaced "excessively" with "more strongly" as follows:

From "therefore, denatured proteins can be excessively regulated by the protein degradation pathway in the late phase after sufficient Hsp70 is synthesized "

To "therefore, denatured proteins can be more strongly regulated by the protein degradation pathway in the late phase after sufficient Hsp70 is synthesized." **[on page 18_line 382–384]**

5. Fig. 1A, right panel - should the lowest strips in the IP and Input panels be labeled "Hsc70" rather than "Hsp70"?

As the reviewer pointed out, we corrected the labelling of Hsc70 in right panel of Fig. 1a.

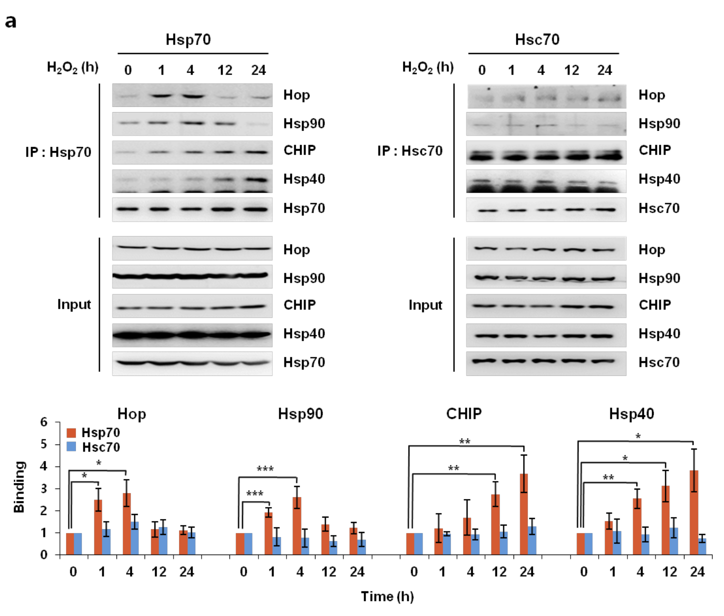

Figure 1a. After stress, Hsp70, but not Hsc70, gradually changes its co-chaperone complexes from protein refolding machinery (1–4 h) to degradation machinery (12–24 h). After treating HEK293T cells with 1 mM H_2O_2, endogenous Hsp70 and Hsc70 were precipitated and their co-chaperone binding partners were identified by western blotting (top). Quantification of relative co-chaperone binding of Hsp70 and Hsc70 (bottom).

6. Fig. 1A/B - histograms - the values plotted are normalized relative to an arbitrary value of 1 at time = 0 hours for both Hsp70 and Hsc70. It would, however, be interesting to compare the intensities (propensity to bind) relative to each other between Hsp70 and Hsc70. Do Hsc70 and Hsp70 actually bind Hop (or CHIP) similarly at time zero, or do they intrinsically have somewhat differing affinities for these cochaperones? From IPs in panels A, it appears superficially that Hsc70 binds more strongly to CHIP and more weakly to Hop even at time zero but it would be interesting to quantitate this.

As the reviewer pointed out, it would be interesting to compare the propensity of Hsp70 and Hsc70 to bind to co-chaperones. However, this comparison is not possible using the data from Fig. 1a in this manuscript. Because we used different antibodies for Hsp70 and Hsc70, which may have unequal efficacy for immunoprecipitation and western blotting, the intensity of the bands of western blotting in Fig. 1a do not represent the absolute amounts of each protein.

Instead, to quantitatively compare the binding affinity of Hsp70 and Hsc70 to its co-chaperones at time zero, we transfected the cells with the Myc-tagged Hsp70 and Myc-Hsc70 plasmids and then performed immunoprecipitation using an anti-Myc antibody. As shown in Revision Letter Fig. 12, Myc-Hsp70 showed preferential binding to Hop and Hsp90 compared to Myc-Hsc70, while Myc-Hsc70 showed preferential binding to CHIP and Hsp40 (Revision Letter Fig. 12). Interestingly, these patterns are very similar to the result shown in Fig. 5a, which shows the difference in co-chaperone binding patterns of wild-type and K77R mutant Hsp70 at time zero (Fig. 5a). In our study, we showed that the K77 residue is conserved in various species in Hsp70, but not in Hsc70; thus, Hsp70, but not Hsc70, was acetylated by ARD1 (Fig. 2d, Fig. 4a). Thus, Hsc70 showed a similar co-chaperone binding pattern to the K77R mutant Hsp70. Therefore, K77 acetylation may affect the differing propensity of Hsp70 vs Hsc70 to bind to co-chaperones in cells.

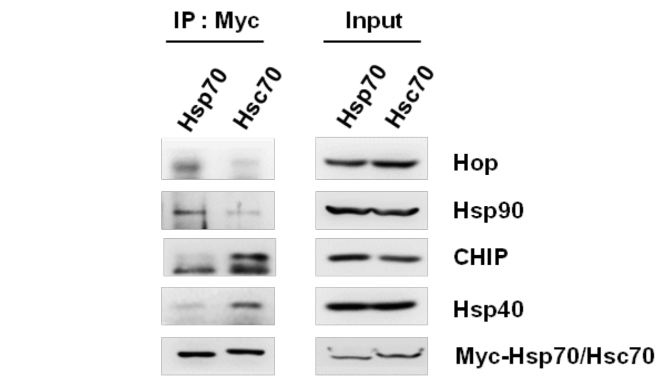

Revision Letter Figure 12. Hsp70 and Hsc70 show different propensities for binding to co-chaperones. Myc-Hsp70 WT and K77R were precipitated using an anti-Myc antibody from HEK293T cells, and their binding to co-chaperones was analyzed by western blotting.

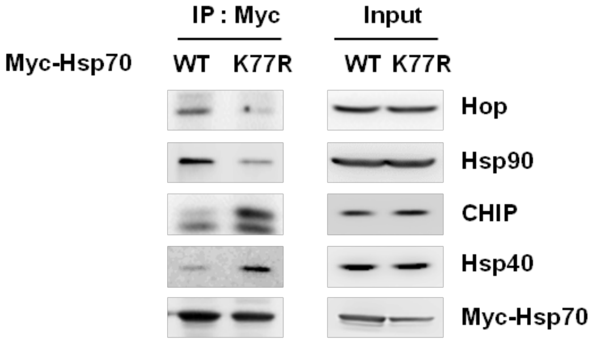

Figure 5a. K77 acetylation controls the co-chaperone binding complexes of Hsp70. Myc-Hsp70 WT and K77R were precipitated from HEK293T cells and the co-precipitated co-chaperones were assessed by western blotting.

7. Fig. 1A/B histograms - Also, I am curious as to why the zero time points don't show error bars in this plot and panel C - did everything get normalized to a single time zero value, or were the time zero values of the individual trials each considered to be "1" for that trial?

We performed three independent trials in different gels at different times; thus, we could not compare the time zero values of each trial. In each trial, we considered time zero value of the individual trial to be "1", and normalized the relative values of the other time points. This method is typically used to quantify the results of independent western blot experiments[10, 11, 12].

8. Fig. 1C, right panel - are the measures of significance relative to the immediately preceding data point? It's not clear.

The significance was tested against the control group (0 h). We have incorporated this information into the Fig. 1c legend.

9. Fig. 4D; The nature of the ATP binding experiment is unclear from the figure and the legend only, though it is explained in the methods section. It would be helpful if the authors could briefly mention in the figure legend to the fact that Hsp70/Hsc70- are being pulled down with ATP-agarose beads in this experiment.

As the reviewer suggested, we included more detailed information regarding the ATP binding assay in the Fig. 4d legend.

10. Fig. 5E-G. Similarly, the nature of the protein refolding, degradation and aggregation assays in panels E-G are unclear unless the reader looks at the methods section. A brief mention of the nature of each assay in the figure legend would be very helpful.

As the reviewer suggested, we briefly described the methods for protein refolding, degradation and aggregation experiments in the Fig. 5e–g legends.

11. Statistics in the manuscript are mostly appropriately used, in my estimation. The number of trials in most of the western blot assays are low (typically n=3) but no more so than typical for these types of assays; they are appropriately evaluated using two-tailed Student's t-tests. I raise a slight concern about the zebrafish locomotor activity measurements shown in Figure S7, panels E/F where a normal distribution may not apply due to the zero value cutoff, so a different significance test may be more appropriate.

We appreciate the reviewer's helpful comment. We changed the statistical analysis a more appropriate method for our experiments. As reviewer pointed out, measured locomotor activity values did not show a normal distribution, and thus we performed Kruskal-Wallis ANOVA with Dunn's pairwise multiple comparison test. We have added this to the Methods section and indicated the recalculated statistical values in the corresponding figure legends (Supplementary Fig. 7e and f).

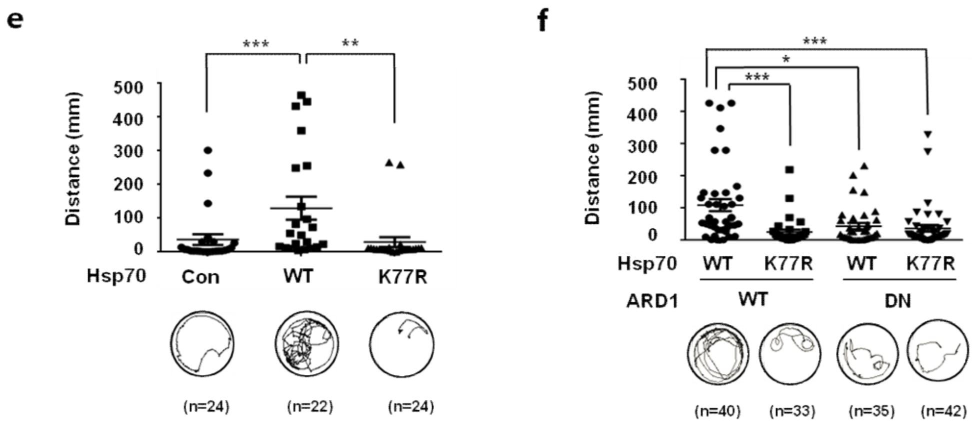

Supplementary Figure 7e and f. ARD1-mediated Hsp70 acetylation imporoves locomotor activity of zebrafish. (e) Hsp70 acetylation enhances the locomotor activity of zebrafish. Zerafish expressing GFP-Hsp70 WT and K77R were treated with MPTP 10 ug/ml at 1–5 dpf, then the locomotor activities of zebrafish were measured at 5 dpf (top). Embryos were adjusted to chamber for 5 min and tracked for 10 min. Representative swimming patterns of MPTP-treated zebrafish (bottom).

(f) ARD1 improves the locomotor activity of zebrafish through Hsp70 acetylation. Zebrafish co-expressing Myc-ARD1 and GFP-Hsp70 were treated with 10 ug/ml MPTP at 1–5 dpf, the locomotor activities of zebrafish were measured at 5 dpf (top). Embryos were adjusted to chamber for 5 min and tracked for 10 min. Representative swimming patterns of MPTP-treated zebrafish (bottom).

Error bars indicate S.E.M. The number (n) of the embryos examined for quantitative analyses is indicated at the bottom. **$P < 0.005$; ***$P < 0.001$; ****$P < 0.0001$, one-way ANOVA.

Reviewer #3 (Remarks to the Author):

Article Summary:

In this study the authors identify and characterize an acetylation switch on Hsp70 that dictates client refolding (mediated by Hop and Hsp90) versus ubiquitin-dependent degradation (mediated by Hsp40 and CHIP). This acetylation switch involves ARD1-dependent acetylation of Hsp70 on K77, which is evolutionarily conserved. The acetylation-dependent switch has important implications for ATPase activity, client degradation and cell survival. The authors demonstrate their results in both human cell lines and the zebrafish model system.

This is a solid body of work. The experiments are well controlled and thorough.

I have only minor comments:

1) The CHIP signal in some blots appears different than others - sometimes there is an upper band and sometimes not and its unclear why this is. For example, compare Fig 1 A left (single band) and right (doublet, unclear which is CHIP), and 5A (doublet, presumably only upper band is CHIP). The authors should explain this discrepancy and should always indicate background bands with an asterisk.

We appreciate the reviewer's helpful comments regarding our manuscript. When we performed immunoprecipitation (IP), the CHIP signal sometimes appeared as double bands, whereas the CHIP signal of the input samples was always clearly detected as a single band. Thus, we compared the band sizes of CHIP between IP samples and input samples and found that the upper band of the IP samples was CHIP and the lower band was non-specific background. Although we do not know the exact reason why the CHIP signal in the IP samples sometimes showed this pattern, this may depend on the different experimental conditions, such as the acryl amide percentage in SDS gel and freshness of antibodies used for immunoprecipitation and blocking solution.

According to the reviewer's comment, we have indicated the background band with an asterisk and described this in the figure legends of Fig. 1a and Fig. 5a in the revised manuscript.

2) The authors should indicate the percentage input and IP loaded for ARD1 and HDAC4-Hsp70 interactions. This is important because it will give an idea of the fraction of Hsp70 that is in complex with these enzymes. This could have important implications for the model of how Hsp70 acetylation is regulated. For example, is most Hsp70 complexed with ARD1? If so, is the interaction modulated over time or by stress? My intention is not necessarily to have the authors test this, but it is an important point for discussion, which is facilitated by knowing the percent of each protein population in the complex.

We appreciate the reviewer's valuable comments and suggestions. In general, we used 1 mg protein for immunoprecipitation (IP) and loaded 20 μg protein for the input sample; this information is indicated in the Methods section of the revised manuscript. As the reviewer commented, we showed the interaction of ARD1 and HDAC4 with Hsp70 in Fig. 2a and Supplementary Fig. 5a. Although we loaded the same amount of protein for IP (1 mg) and input (20 μg) in each experiment, it is difficult to compare the protein binding between different experiments. Because we performed these experiments at different times, many experimental conditions, such as plasmid transfection efficiency, immunoprecipitation efficiency, antibody sensitivity and exposure time for blotting, all of which can affect the band intensity, may differ in these independent experiments. Therefore, these data are not sufficient for determining the exact component proportion ratio of HDAC4 and ARD1 in the Hsp70 complex.

However, we think this suggestion is valuable and hope to test how the bindings of ARD1 and HDAC4 to Hsp70 change over time during cellular stress. It is possible that ARD1 strongly binds to Hsp70 in the early phase after stress and then gradually detaches over time. Instead, HDAC4 binding to Hsp70 may be weak in the early phase and become stronger in the late phase after stress. We have incorporated this in the revised manuscript as follows:

"In this study, we found that HDAC4 potently deacetylates Hsp70 K77. HDAC4 directly deacetylated the K77 acetylation of Hsp70 *in vitro* and changed its co-chaperone binding. Based on the acetylation/deacetylation state, we assume that Hsp70 may bind more strongly to ARD1 in the early phase after stress, then Hsp70 may gradually convert its binding partner from ARD1 to HDAC4 over time. Therefore, further studies are needed to investigate the component proportion ratio of HDAC4 and ARD1 in the Hsp70 complex and its modulation over time after cellular stress." **[on page19_line 405–412]**

3) Figure 4D and the meaning of Hsp70-ATP needs to be better explained. In general, the figure legends could be a bit more descriptive.

As the reviewer suggested, we have incorporated more detailed information into the Fig. 4d legend. In addition, we also added some description to the Fig. 2b, Fig. 2c, Fig. 5e, Fig. 5f, Fig. 5g, Supplementary Fig. 7a, b, e and f legends.

Reviewer #4 (Remarks to the Author):

The mass spectrometric identification of the claimed acetylation site looks very solid. I carefully looked at the tandem mass spectrum of the K77 acetylated HSP70 peptide in Figure 3D and manually annotated the spectra to validate the identification. All the major backbone (b- and y-type) fragment ions are present and as expected from the peptide sequence the most abundant fragment ions are b4 & y8, which is caused by the Asp(D)-Pro(P) cleavage - the weakest peptide bond of all. I am therefore confident that the interpretation made by the authors is correct.

However, this identification of HSP70-K77 acetylation is based on in-vitro acetylation and have never been identified in-vivo,- although lots of in-vivo acetylation sites on HSP70 have been described previously from large-scale proteomics experiments (see for example: Choudhary et al, Science 2009 and Lundby et al, Cell Reports 2012). On the other hand, K77 has previously been identified as a ubiquitylation site (ref. Wagner SA, et al. Mol Cell Proteomics 2011), so the K77R and K77Q mutants would also prevent and interfere with this regulatory PTM! This should be considered by the authors.

We appreciate the reviewer's valuable comment. In our study, we attempted to identify the acetylation site of Hsp70, which is directly acetylated by ARD1; thus, we performed *in vitro* acetylation using ARD1 and Hsp70 recombinants and then subjected the samples to LC-MS/MS. Although we identified the K77 site *in vitro*, using a K77R mutant Hsp70 and K77 acetylation-specific antibody we confirmed that K77 acetylation of Hsp70 occurred in cells after stress. Although many studies have previously identified post-translational modifications of Hsp70 in cells and tissues, K77 acetylation has not been observed[13, 14]. One possible explanation for this is that in this study, we showed that K77 acetylation of Hsp70 was stimulated by cellular stress; thus, under normal conditions, the acetylation level of K77 of Hsp70 was likely too low to be detected by LC-MS/MS.

Regarding the reviewer's second comment related to K77 ubiquitination of Hsp70, we agree that the K77R and K77Q mutants would also prevent ubiquitination of this site. If the diverse cellular effects of these mutations in our study were related to K77 ubiquitination of Hsp70, these two mutations should show similar patterns following inhibition of K77 ubiquitination. However, in our study, K77R and K77Q showed opposing effects on co-chaperone binding and protein refolding/degradation because they represent opposing states of

Hsp70 acetylation; K77R and K77Q mutants mimic the deacetylated and acetylated forms, respectively. Therefore, our results obtained using the K77R and K77Q mutants likely represent the roles of K77 acetylation of Hsp70 rather than its ubiquitination.

Reviewer #5 (Remarks to the Author):

In this manuscript, the authors investigated the role and interplay of Hsp70 and ARD1 on protein folding/degradation and cell survival under stress conditions.

As I am not really an expert in this specific field, I did not review in details most of this manuscript. Nonetheless, I am impressed by the quantity of experiments and techniques used.

In regard to the in vivo part, I have however several concerns as I found this part poorly described and not really clear.

1. For example, they said in page 15 that "Hsp70 K77R mutation reduced the zebrafish survival ratio (Supplementary Fig. 7a)". This sentence does not make sense as it is presented. One can understand that injection of Hsp70 RNA presenting K77R mutation would reduce zebrafish survival in any conditions (stress or no stress). They do not present non-injected embryos survival rate, thus they cannot make this claim. I suppose they actually wanted to say that Hsp70 K77 mutation reduced the protective effect of HSP70. If it is the case they should still present survival ratio of non-injected embryos to evaluate the protective effect of HSP70 RNA injection. On top of that, no statistics are presented for those experiments. In theory, they should repeat several times this work and present some sort of statistical analysis. Without that, the authors cannot make any conclusions or claims. This is purely descriptive.

We appreciate the reviewer's valuable comments on our manuscript pointing out our poor description of experiments and missing information. We have improved those previous descriptions and clarified the missing experimental procedures.

As the reviewer commented, we stated that the K77 mutation in Hsp70 blocked the protective effect of Hsp70 under hydrogen peroxide-induced oxidative stress. We also repeated the experiment with a mock-injected control group, and triplicate results were statistically analysed (Supplementary Fig. 7a and b). These new results have been replaced in Supplementary Fig. 7a and b. We have also changed the ambiguous sentence as follows:

From "Consistent with the *in vitro* data shown in Fig. 6a, Hsp70 K77R mutation reduced the zebrafish survival ratio."

To "Consistent with the *in vitro* data shown in Fig. 6a, the K77 mutation in Hsp70 reduced the protective effect of HSP70 against hydrogen peroxide–induced oxidative stress (Supplementary Fig. 7a)." **[on page 15_line 308–310]**

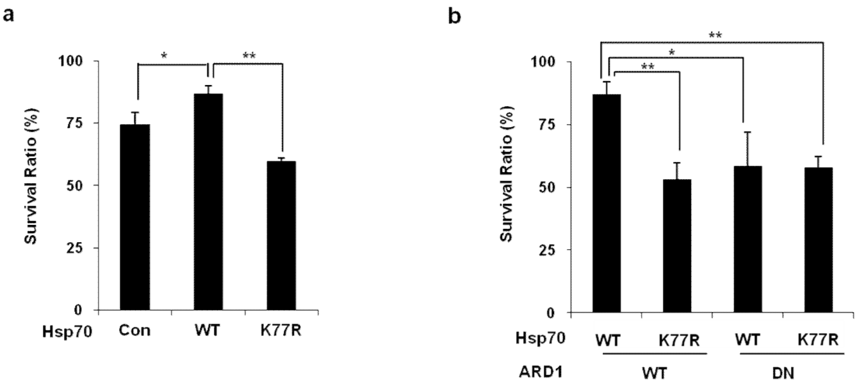

Supplemental Figure 7a and b. ARD1-mediated Hsp70 acetylation protects cells against stress conditions *in vivo*.

(a) Hsp70 acetylation rescues zebrafish lethality. Zebrafish expressing GFP-Hsp70 WT and K77R were incubated with 5 mM H_2O_2 for 1 h at 3 dpf, and the survival ratios of zebrafish were measured on the following day.

(b) ARD1 increases zebrafish survival through Hsp70 acetylation. WT or DN Myc-ARD1 were co-expressed with GFP-Hsp70 in zebrafish. At 3 dpf, embryos were treated with 5 mM H_2O_2 for 1 h, and the survival ratios were measured on the following day.

Error bars indicate S.D. (n = 3). **P* < 0.05; ***P* < 0.005, *t* test.

2. All of the in vivo experiments described in this manuscript are based on RNA injections.

a. It is not clear if they produced themselves the plasmids pEGFP-C3-Hsp70 and pCS2+-ARD1 presented in the method section; I guess so. They should clarify that and, if they did produce these plasmids, they should present the cloning steps. Moreover, which material did they use to produce the mutated RNA

forms? I may miss something here.

We have cloned the wild-type and mutant plasmids of Hsp70 and ARD1. In detail, full-length cDNAs for human Hsp70 (Genbank: NM_005355.5) and human ARD1 (Genbank: NM_003491.3) were obtained by PCR and inserted into the pEGFP-C3 (GFP-Hsp70) and pCS2+ (Myc-ARD1) vectors. Point mutations in Hsp70 K77 and ARD1 R82/Y122 (DN-ARD1) were generated using the Muta-Direct Site Directed Mutagenesis kit (Intron). Cloned plasmids were linearised and transcribed using the mMESSAGE mMACHINE Kit (Ambion). Based on the reviewer's comment, we clarified the details of the cloning steps in 'siRNA and plasmid construction' and '*In vitro* mRNA transcription' in the Methods section.

b. They did not confirm if the injected RNA was properly translated. It would be important to see some sort of confirmation of the presence of the corresponding protein. They developed specific antibodies in this study, so it might be a quick validation procedure (They need to test the antibody on non-injected fish too). Moreover, they analysed the effect of such injections in animals up to 5dpf. Injected RNA is most likely fully degraded at 5dpf. So it would be interesting to see if the protein is still present or when it starts to decline. It is interesting to see if such injection have a long lasting protective effect as they seem to show.

As pointed out by the reviewer, we first verified the expression of injected mRNA when setting up the experiments. After RNA injection at the one-cell stage, we first observed the GFP-Hsp70 expression by fluorescent microscopy at 1 dpf (Revision Letter Fig. 13a). The maintenance of and the decrease in expression at later time points was analyzed by western blotting (Revision Letter Fig. 13b). Expression of the injected mRNA was strongest at 3 dpf and subsequently began decreasing, but was maintained until 5 dpf when the experiments were completed. We also compared the expression of GFP-Hsp70 protein with the control group at 4 dpf (Revision Letter Fig. 13c). Because hydrogen peroxide and MPTP were treated at 3 dpf and 1–5 dpf, respectively, over-expressed proteins were effective over our assay time.

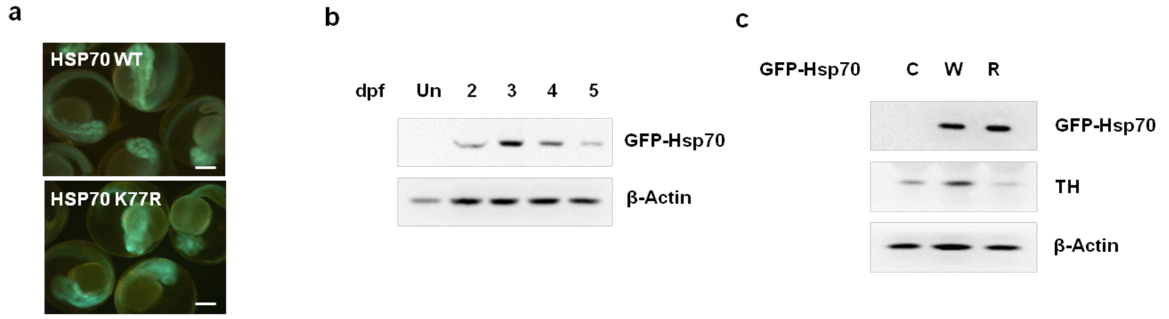

Revision Letter Figure 13. Expression of GFP-Hsp70 in zebrafish.

(a) Expression of GFP-Hsp70 in zebrafish embryos. Embryos injected with GFP-Hsp70 WT and K77R mutant mRNA at one cell stage were observed under fluorescence microscope at 1 dpf. Scale bars, 200 μm.

(b) Time-dependent expression of GFP-Hsp70 in zebrafish embryos. Embryos injected with GFP-Hsp70 mRNA at the one-cell stage were homogenised at the indicated time points (2–5 dpf) and GFP-Hsp70 expression was analyzed by western blotting.

(c) Hsp70 acetylation protects dopaminergic neurons against MPTP-induced cell death. Zebrafish expressing GFP-Hsp70 WT and K77R were treated with 10 μg/mL MPTP at 1–4dpf. Embryos were then homogenised and GFP-Hsp70 and dopaminergic neuron marker TH expression was analyzed by western blotting.

TH; tyrosine hydroxylase, Un; uninjected embryos, C; mock-injected embryos, W; wild-type, R; K77R mutant.

3. On the same page, authors said "Functionally, the K77R mutation markedly decreased zebrafish locomotor activity compared with that of wild-type Hsp70 (Supplementary Fig. 7e), and expression of dominant-negative mutant ARD1 eliminated the distinguishable Hsp70 effects (Supplementary Fig 7f)." This is not clear and they should review their statement. What the distinguishable Hsp70 effects ? They did not show any non-injected fish, so what is the Hsp70 effect compared to ?

As the reviewer suggested, we performed the experiment including a control group. Under the condition of MPTP-induced locomotor activity loss, wild-type Hsp70-expressing zebrafish showed higher locomotor activity than the control and this effect was lost in Hsp70 K77R- or DN-ARD1-expressing embryos (Supplementary Fig. 7e and f). We have changed the sentence to clarify the meaning as follows:

From "Functionally, the K77R mutation markedly decreased zebrafish locomotor activity compared with that of wild-type Hsp70 (Supplementary Fig. 7e), and expression of dominant-negative mutant ARD1 eliminated the distinguishable Hsp70 effects (Supplementary Fig 7f)"

To "Functionally, the K77R mutation markedly decreased zebrafish locomotor activity compared to that in wild-type Hsp70 (Supplementary Fig. 7e), and expression of the dominant-negative mutant ARD1 eliminated the protective effects of wild-type Hsp70 (Supplementary Fig 7f)." **[on page 15_line 316–319]**

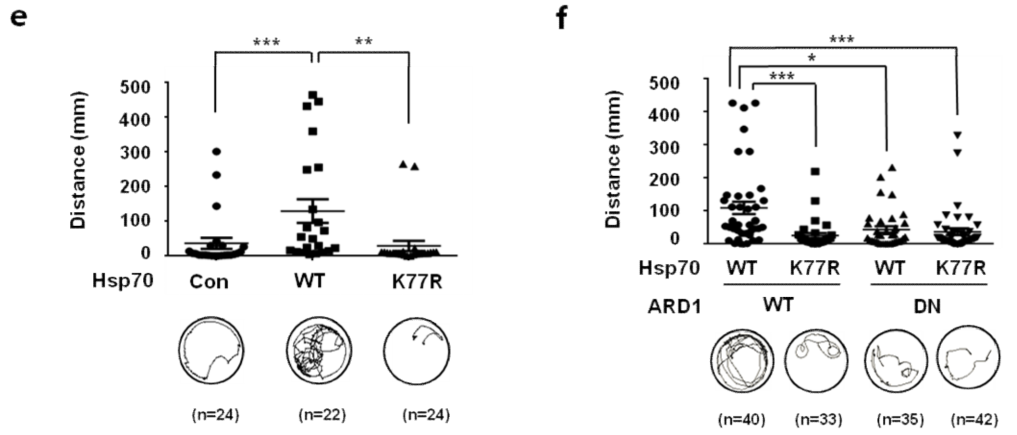

Supplementary Figure 7e and f. ARD1-mediated Hsp70 improves the locomotor activity of zebrafish.

(e) Hsp70 acetylation enhances the locomotor activity of zebrafish. Zerafish expressing GFP-Hsp70 WT and K77R were treated with MPTP 10 ug/ml at 1–5 dpf, then the locomotor activities of zebrafish were measured at 5 dpf (top). Embryos were adjusted to chamber for 5 min and tracked for 10 min. Representative swimming patterns of MPTP-treated zebrafish (bottom).

(f) ARD1 improves the locomotor activity of zebrafish through Hsp70 acetylation. Zebrafish co-expressing Myc-ARD1 and GFP-Hsp70 were treated with 10 ug/ml MPTP at 1–5 dpf, the locomotor activities of zebrafish were measured at 5 dpf (top). Embryos were adjusted to chamber for 5 min and tracked for 10 min. Representative swimming patterns of MPTP-treated zebrafish (bottom).

Error bars indicate S.E.M. The number (n) of the embryos examined for quantitative analyses is indicated at the bottom. $^{**}P < 0.005$; $^{***}P < 0.001$; $^{****}P < 0.0001$, one-way ANOVA.

4. Figures S7A and S7B. How old are those embryos/larvae and when the treatment was done? I cannot find these important informations.

We appreciate the reviewer’s comment pointing out the missing information. Several points were omitted during manuscript preparation. We incubated 3 dpf embryos with 5 mM peroxide for 1 h and observed their survival ratios on the following day. We have incorporated this information into the Supplementary Fig. 7a and b legends and the Methods section.

5. They used different chemical treatments to induce stress/death in zebrafish. They should present references to previous descriptive experiments.

We included the references in 'Immunohistochemistry', 'Zebrafish survival rate' and 'Behavioral analysis' in the Methods section.

6. Figure S7E and S7F. Authors should state in the legend how old are those fish (it is presented in the method section but it is not convenient for the reader). These experiments were performed in dark or light condition?

For the locomotor activity assay, the light intensity of the observation chamber was set to 4,400 lux and embryos were first adapted to the chamber for 5 min before recording. Next, locomotor activity was observed for a 10 min period. We have indicated this information in the Supplementary Fig. 7e and f legends and 'Behavioral analysis' in the Methods section in the revised manuscript.

7. Figure S7A legend. Authors said "the survival ratios of zebrafish were measured by western blotting." I guess they forgot to review this sentence...

We apologize for this lapse and corrected the sentence as follows:

From "the survival ratios of zebrafish were measured by western blotting."

To "the survival ratios of zebrafish were measured on the following day."

8. Figure S7(C and D). Authors said "were immunostained (left) and counted (light) (c)." Does light should be right?

We corrected the wording as follows:

From "were immunostained (left) and counted (light) (c)."

To "were immunostained (left) and counted (right) (c)."

References

1. Zeke, T., Morrice, N., Vazquez-Martin, C. & Cohen, P.T. Human protein phosphatase 5 dissociates from heat-shock proteins and is proteolytically activated in response to arachidonic acid and the microtubule-depolymerizing drug nocodazole. *Biochem. J.* **385**, 45-56 (2005).
2. Shalgi, R. *et al.* Widespread regulation of translation by elongation pausing in heat shock. *Mol. Cell* **49**, 439-452 (2013).
3. Muller, P. *et al.* C-terminal phosphorylation of Hsp70 and Hsp90 regulates alternate binding to co-chaperones CHIP and HOP to determine cellular protein folding/degradation balances. *Oncogene* **32**, 3101-3110 (2013).
4. Laskowski, R.A., Gerick, F. & Thornton, J.M. The structural basis of allosteric regulation in proteins. *FEBS Lett.* **583**, 1692-1698 (2009).
5. Barford, D., Hu, S.H. & Johnson, L.N. Structural mechanism for glycogen phosphorylase control by phosphorylation and AMP. *J. Mol.Biol.* **218**, 233-260 (1991).
6. Edreira, M.M. *et al.* Phosphorylation-induced conformational changes in Rap1b: allosteric effects on switch domains and effector loop. *J.Biol.Chem.* **284**, 27480-27486 (2009).
7. McDonald, L.R., Whitley, M.J., Boyer, J.A. & Lee, A.L. Colocalization of fast and slow timescale dynamics in the allosteric signaling protein CheY. *J.Mol.Biol.***425**, 2372-2381 (2013).
8. Assimon, V.A., Southworth, D.R. & Gestwicki, J.E. Specific Binding of Tetratricopeptide Repeat Proteins to Heat Shock Protein 70 (Hsp70) and Heat Shock Protein 90 (Hsp90) Is Regulated by Affinity and Phosphorylation. *Biochemistry* **54**, 7120-7131 (2015).
9. Rohl, A. *et al.* Hop/Sti1 phosphorylation inhibits its co-chaperone function. *EMBO Rep.* **16**, 240-249 (2015).
10. Cook, P.J. *et al.* Tyrosine dephosphorylation of H2AX modulates apoptosis and survival decisions. *Nature* **458**, 591-596 (2009).
11. Park, J. *et al.* RAS-MAPK-MSK1 pathway modulates ataxin 1 protein levels and toxicity in SCA1. *Nature* **498**, 325-331 (2013).
12. Cha, J.H. *et al.* Prompt meningeal reconstruction mediated by oxygen-sensitive AKAP12 scaffolding protein after central nervous system injury. *Nature communications* **5**, 4952 (2014).
13. Choudhary, C. *et al.* Lysine acetylation targets protein complexes and co-regulates major cellular functions.

Science **325**, 834-840 (2009).

14. Lundby, A. *et al.* Proteomic analysis of lysine acetylation sites in rat tissues reveals organ specificity and subcellular patterns. *Cell Rep.* **2**, 419-431 (2012).

Reviewers' Comments:

Reviewer #1 (Remarks to the Author)

The additions to the revised manuscript, and information in the revision letter, greatly improve the credibility. One of the major questions previously was whether the mechanism applies during the recovery from acute stress, not just in the harsh chronic stress used in most of the manuscript. The extensive rebuttal to point 1 appears to show this. The new experiments in zebrafish using milder conditions are also reassuring.

My recommendations are:

1. Include the revision letter figures 2, 4 and 5 as supplemental data.

2. Revision letter figure 9 should also be in the supplemental. This is important as the refolding procedure used here has never been used before, and its validation has to be shown.

3. It would be nice though not necessary to show revision letter figure 7 in supplemental, that NaCl and Ethanol stress also induce the mechanism. The heat shock experiment in revision letter fig. 6 is inconclusive, as the input blots are inconsistent with the established heat shock response. There is no induction of Hsp70 and Hsp90, which are known to induce around 2-fold in HEK293T cells, including in quantitative proteomics reports. Similarly, CHIP is not induced at all in previous papers, but increases more than 2-fold here. For example see Ryno et al. 2014, ACS Chem. Biol. 9, 1273-1283. Whatever stress the cells are under is not producing a classical heat shock response. That is fine, there are many types of stresses that can damage cells. But for that reason the additional information about NaCl and ethanol stress is helpful. However, I will leave this up to the authors and editor as the supplemental is already long.

Reviewer #2 (Remarks to the Author)

This is a resubmission with revisions of manuscript NOCMMS-16-05509A, entitled "ARD1-Mediated Hsp70 Acetylation Balances Stress-Induced Protein Refolding/Degradation". In my original positive review of the first submission of the manuscript, I noted that "This is a novel, important and well-conducted study that will be of substantial interest to investigators in the chaperone / protein-quality control field that reveals a compelling new mechanism for cochaperone-switching and for the functional changes that accompany cochaperone-switching." I had several minor criticisms that the authors have addressed satisfactorily. In addition, the authors have (in my opinion) substantially and fairly comprehensively addressed points and criticisms raised by the other reviewers. I feel this an improved and even better manuscript and I

support its publication in Nature Communications.

Reviewer #3 (Remarks to the Author)

At this point, all of my minor concerns have been addressed and I recommend the work for publication.

Reviewer #5 (Remarks to the Author)

The authors have answered all pending issues and modified the manuscript accordingly. Overall, I find this version greatly improved and should be well received by the readers.

Responses appear below the reviewers' comments

Reviewer #1:

The additions to the revised manuscript, and information in the revision letter, greatly improve the credibility. One of the major questions previously was whether the mechanism applies during the recovery from acute stress, not just in the harsh chronic stress used in most of the manuscript. The extensive rebuttal to point 1 appears to show this. The new experiments in zebrafish using milder conditions are also reassuring.

My recommendations are:

1. Include the revision letter figures 2, 4 and 5 as supplemental data.

We really appreciate the reviewer's helpful comments on our manuscript. As the reviewer suggested, we have incorporated the revision letter figures 2, 4 and 5 into supplementary figure 6, 8 and 9, respectively.

2. Revision letter figure 9 should also be in the supplemental. This is important as the refolding procedure used here has never been used before, and its validation has to be shown.

As commented by the reviewer, we have incorporated the revision letter figures 9 into supplementary figure 7.

3. It would be nice though not necessary to show revision letter figure 7 in supplemental, that NaCl and Ethanol stress also induce the mechanism. The heat shock experiment in revision letter fig. 6 is inconclusive, as the input blots are inconsistent with the established heat shock response. There is no induction of Hsp70 and Hsp90, which are known to induce around 2-fold in HEK293T cells, including in quantitative proteomics reports. Similarly, CHIP is not induced at all in previous papers, but increases more than 2-fold here. For example see Ryno et al. 2014, ACS Chem. Biol. 9, 1273-1283. Whatever stress the cells are under is not producing a classical heat shock response. That is fine, there are many types of

stresses that can damage cells. But for that reason the additional information about NaCl and ethanol stress is helpful. However, I will leave this up to the authors and editor as the supplemental is already long.

As the reviewer suggested, we have incorporated the revision letter figure 7 into supplementary figure 1c and d in the revised manuscript. In accordance with changes in supplementary figure 1, we have altered the sentence in the Result section as follows:

"To confirm our results under other physiological stress conditions, cellular stresses were induced by various reagents, including etoposide (a DNA-damaging reagent), 1-methyl-4-phenylpyridinium (MPP^+, a neurotoxin), sodium chloride (hyperosmotic stress) and ethanol. Following treatment, Hsp70 acetylation rapidly increased and the co-chaperone binding changed accordingly (Supplementary Fig. 1a-d)." **[on page7_line 125–129]**

Reviewer #2 (Remarks to the Author):

This is a resubmission with revisions of manuscript NOCMMS-16-05509A, entitled "ARD1-Mediated Hsp70 Acetylation Balances Stress-Induced Protein Refolding/Degradation". In my original positive review of the first submission of the manuscript, I noted that "This is a novel, important and well-conducted study that will be of substantial interest to investigators in the chaperone / protein-quality control field that reveals a compelling new mechanism for cochaperone-switching and for the functional changes that accompany cochaperone-switching." I had several minor criticisms that the authors have addressed satisfactorily. In addition, the authors have (in my opinion) substantially and fairly comprehensively addressed points and criticisms raised by the other reviewers. I feel this an improved and even better manuscript and I support its publication in Nature Communications.

Reviewer #3 (Remarks to the Author):

At this point, all of my minor concerns have been addressed and I recommend the work for publication.

Reviewer #5 (Remarks to the Author):

The authors have answered all pending issues and modified the manuscript accordingly. Overall, I find this version greatly improved and should be well received by the readers.
